# Nuclear Magnetic Resonance and Metadynamics Simulations Reveal the Atomistic Binding of l-Serine and *O*-Phospho-l-Serine at Disordered Calcium Phosphate Surfaces of Biocements

**DOI:** 10.1021/acs.chemmater.2c02112

**Published:** 2022-09-26

**Authors:** Renny Mathew, Baltzar Stevensson, Michael Pujari-Palmer, Christopher S. Wood, Phillip R. A. Chivers, Christopher D. Spicer, Hélène Autefage, Molly M. Stevens, Håkan Engqvist, Mattias Edén

**Affiliations:** † Department of Materials and Environmental Chemistry, Stockholm University, Stockholm SE-106 91, Sweden; ‡ Applied Material Science, Department of Engineering, Uppsala University, Uppsala SE-751 21, Sweden; § Department of Medical Biochemistry and Biophysics, Karolinska Institute, Stockholm SE-171 77, Sweden; ∥ Department of Chemistry, University of York, Heslington, York YO10 5DD, U.K.; ⊥ Department of Materials, Department of Bioengineering, and Institute of Biomedical Engineering, Imperial College London, London SW7 2AZ, U.K.

## Abstract

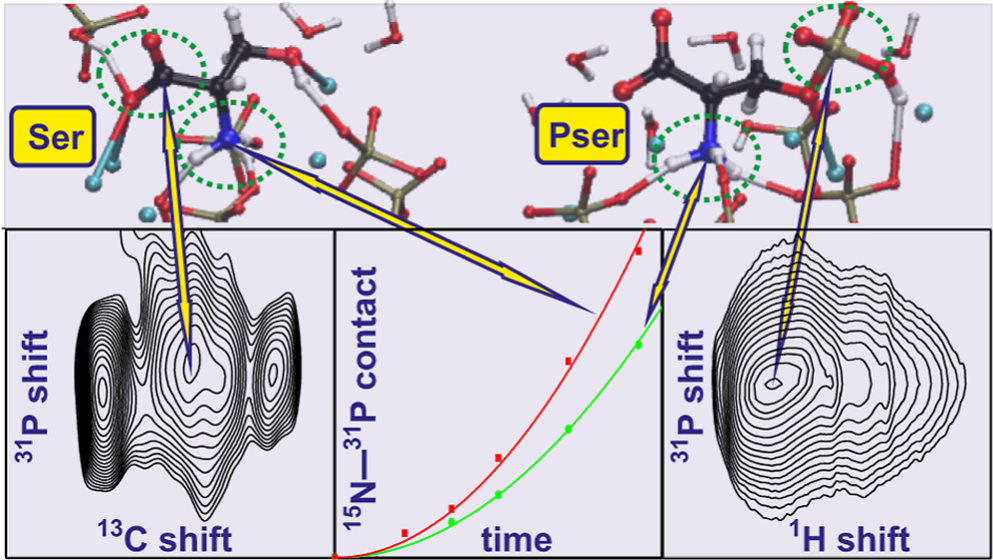

Interactions between biomolecules and structurally disordered calcium phosphate (CaP) surfaces are crucial for the regulation of bone mineralization by noncollagenous proteins, the organization of complexes of casein and amorphous calcium phosphate (ACP) in milk, as well as for structure–function relationships of hybrid organic/inorganic interfaces in biomaterials. By a combination of advanced solid-state NMR experiments and metadynamics simulations, we examine the detailed binding of *O*-phospho-l-serine (Pser) and l-serine (Ser) with ACP in bone-adhesive CaP cements, whose capacity of gluing fractured bone together stems from the close integration of the organic molecules with ACP over a subnanometer scale. The proximity of each carboxy, aliphatic, and amino group of Pser/Ser to the Ca^2+^ and phosphate species of ACP observed from the metadynamics-derived models agreed well with results from heteronuclear solid-state NMR experiments that are sensitive to the ^13^C–^31^P and ^15^N–^31^P distances. The inorganic/organic contacts in Pser-doped cements are also contrasted with experimental and modeled data on the Pser binding at nanocrystalline HA particles grown from a Pser-bearing aqueous solution. The molecular adsorption is driven mainly by electrostatic interactions between the negatively charged carboxy/phosphate groups and Ca^2+^ cations of ACP, along with H bonds to either protonated or nonprotonated inorganic phosphate groups. The Pser and Ser molecules anchor at their phosphate/amino and carboxy/amino moieties, respectively, leading to an extended molecular conformation across the surface, as opposed to an “upright standing” molecule that would result from the binding of one sole functional group.

## 1 Introduction

Bone mineral constitutes nanoparticles of a structurally disordered and carbonate-bearing form of Ca hydroxyapatite [HA; “apatite”; Ca_10_(PO_4_)_6_(OH)_2_].^1−5^ Vast research efforts have been spent to understand the mechanisms of bone mineralization,^5−10^ which remain controversial but are believed to be governed by noncollagenous proteins (NCPs) that carry a high density of negatively charged carboxy-bearing (Asp/Glu) and phosphorylated residues.^5,9−11^ For decades, mainstream models for the NCP/mineral interactions involved “charge-matching” arguments assuming that the protein adopts a secondary structure with its negatively charged sidechains matching the Ca^2+^ positions at the apatite mineral surface to provide epitaxial crystal growth.^6,7^ However, doubts thereof arose from the subsequently accumulating evidence that (i) the crystal-binding domains of most NCPs lack a well-defined secondary structure^10,11^ and (ii) both synthetic and biogenic nanocrystalline apatite particles formed from aqueous solutions—such as body fluids—consist of a “core” of an ordered HA lattice coated by a 1–2 nm thick surface layer (“shell”) of *amorphouscalcium phosphate* (ACP),^4,12−16^ often termed the “hydrated surface layer”.^2,3,17^ These recent insights have led to a paradigm shift advocating simpler electrostatic models for which the net charge of the protein and its underlying distribution control the NCP binding at biogenic apatite.^18−21^

An accurate atomistic probing of biomolecular adsorption at structurally disordered inorganic calcium phosphate (CaP) surfaces by experimental techniques is hampered by the particle fragility, coupled with their structural disorder. The most detailed current insight is provided by magic-angle-spinning (MAS) nuclear magnetic resonance (NMR) experiments, often utilizing proteins with isotopic ^13^C/^15^N labeling at specific sites in its “crystal”-binding domain. Some MAS NMR reports targeted biomolecular binding at bone mineral,^22−26^ but adsorption on model systems of HA nanoparticles is most commonly encountered, encompassing the surface binding of small biomolecules^27−32^ and various mineralization-controlling NCPs such as statherin,^33,34^ osteopontin,^35^ osteonectin,^36,37^ and osteocalcin.^38^ Here, advanced NMR techniques relying on through-space *dipolar interactions*, such as ^13^C{^31^P} and ^15^N{^31^P} rotational-echo double resonance (REDOR)^39^ NMR experiments, have been exploited for obtaining (semi-)quantitative ^13^C–^31^P and ^15^N–^31^P interatomic distance-information along with 2D ^13^C–^13^C correlation NMR experiments that offer constraints on the surface-bound molecular conformation.

Considering the difficulties in experimental probing of the surface binding at atomic resolution, computer modeling by atomistic molecular dynamics (MD) and/or metadynamics simulations offers a rich source of structural information on the organic/inorganic interface.^18−20,40−48^ They may reveal the precise binding sites of the biomolecule along with its detailed surface-immobilized conformation—henceforth referred to as the *binding mode* of the molecule. Yet, outstanding problems in computational modeling of biomolecular adsorption at an *in vitro*- or *in vivo*-generated nanocrystalline apatite surface concern how its structural disorder and potentially pH-dependent phosphate speciation are accounted for. As discussed further in the Supporting Information, these critical aspects remain essentially ignored in all but a few very recent modeling studies.^44−49^

Interfaces between phosphorylated biomolecules and disordered CaP phases are not only of pivotal importance for understanding bone mineralization but also underpin casein–ACP complex-formation in milk^50−53^ and structure–function relationships of hybrid organic/inorganic (bio)materials, such as the bone-tissue-adhesive properties of CaP cements incorporating *O*-phospho-l-serine (Pser),^54−59^ which by their capacity to glue bone tissues together makes them promising for accelerating bone-fracture healing. As unveiled by an array of advanced MAS NMR techniques, we recently demonstrated that the “bone-gluing” strength of such a cement correlates well with its content of an amorphous “ACP/Pser” phase of Pser molecules intimately integrated with ACP across a sub-nm scale.^60^ An analogous “ACP/Ser” component forms in l-serine (Ser) doped cements which, however, exhibit poor bone-adhesive properties,^60^ as discussed and rationalized further herein.

Herein, we advance the atomic-scale insight into the organic/inorganic interface further by examining cements prepared from uniformly ^13^C/^15^N–enriched l-serine and *O*-phospho-l-serine (the latter synthesized herein for the first time), thereby enabling direct experimental probing of the proximity of each carboxy/aliphatic and amino group to the inorganic phosphate moieties of ACP by ^13^C{^31^P} and ^15^N{^31^P} REDOR NMR experimentation, respectively. These results are contrasted with information from heteronuclear ^13^C{^31^P} and ^1^H{^31^P} correlation 2D NMR experiments that reveal the contacts between the organic functional groups of Pser/Ser and the inorganic / phosphate moieties of ACP. We also discuss the similarities and differences between the inorganic/organic contacts in the amorphous ACP/Pser and ACP/Ser biocement components with that of nanocrystalline HA particles grown from a Pser-bearing aqueous solution, henceforth referred to as “Pser@HA”, and constituting a simplified model of a carboxy-bearing or/and phosphorylated protein residue interacting with bone mineral. Moreover, the NMR-derived ^13^C–^31^P and ^15^N–^31^P interatomic-distance constraints were contrasted with results from state-of-the-art well-tempered metadynamics MD simulations^61,62^ employing the INTERFACE force field and the HA-surface preparation protocol of refs (45) and (49) to faithfully model the adsorption of Ser and Pser molecules at the amorphous surface of apatite nanoparticles. These structural models offer a wealth of structural details about the Ser/Pser binding modes (encompassing the surface-bound molecular conformation), as well as a quantification of the relative contribution of each functional group toward the binding *via* a recently introduced analysis protocol.^48^

## 2 Materials and Methods

### 2.1 Preparation of ^13^C/^15^N-Enriched Samples

l-serine (Aldrich;  99.5%) and *O*-phospho-l-serine (Flamma SpA;  95%) with all isotopes at their natural abundance were used as received. [U–^13^C/^15^N]-l-serine enriched to 98% with respect to both ^13^C/^15^N was purchased from CortecNet (France). It was used to prepare [U–^13^C/^15^N]-*O*-phospho-l-serine, which was obtained as its HCl salt (Figure S1) from the corresponding BocNH-Ser-CO_2_-*tert*-Bu analogues *via* phosphoramidation with *tert*-butyl *N*, *N*′-diisopropylcarbamimidate followed by peroxide oxidation to yield the protected phosphate ester, which was converted into the desired product by global deprotection under acidic conditions; see Section S3 for details. These isotopically labeled Ser and Pser powders are for simplicity abbreviated as Ser* and Pser*, respectively. They were used for producing a nanocrystalline HA powder with surface-immobilized Pser* molecules, as well as cements comprising *N* mol % of Ser* or Pser*, which are henceforth denoted by Ser*N* and Pser*N*, respectively.

#### 2.1.1 Pser@HA Specimen

5.0 mL of a 500 mM aqueous solution of CaCl_2_ in deionized water was pipetted in a 20 mL flask placed in a water bath at 37 ± 2 °C, whereupon Pser*·HCl was added to the solution to yield a concentration of 10 mM. 5 mL of 300 mM (NH_4_)_3_PO_4_(aq) was added at  mL/min with continuous magnetic stirring. The pH of the solution was subsequently raised to 7.5 by dropwise addition of 1 M NaOH(aq), leading to precipitation of floccular ACP particles. Deionized water was added to a final volume of 20 mL, and the flask was sealed. The suspension was aged for one week at 37 ± 0.2 °C, leading to a final pH value of 5.3. The as-formed Pser@HA particles were isolated by centrifugation, cleaned twice with deionized water, and then dried in a desiccator at room temperature for 4 days.

The Pser content was estimated as ≈ 5 wt % by contrasting the integrated ^15^N NMR intensity from the Pser@HA specimen with that of the Pser16 cement with known Pser content. The transmission electron microscopy images of Figure S2 reveal agglomerates of crystalline domains with variable sizes (from a few nm to 10–20 nm) that are fused together by ACP. The surface area was estimated to be 159 m^2^/g by the Brunauer–Emmett–Teller model^63^ (N_2_ uptake at relative pressures (*P*/*P*_0_) of 0.05–0.15, using a Micrometrics ASAP2020 volumetric adsorption analyzer).

The Pser@HA synthesis protocol was preceded by extensive testing of optimal preparation conditions by using Pser (Flamma SpA) with concentrations in the 5–200 mM range. As verified by Figure S3, [Pser] ≥ 20 mM yielded products with little/no surface immobilization but predominantly comprised the Ca salt of Pser, Ca[*O*-phospho-l-serine]·H_2_O,^64,65^ henceforth denoted CaPser.

#### 2.1.2 Ser/Pser-Doped Cements

The Ser*N* and Pser*N* cements with *N* = {8, 16, 30} were prepared in batches of 0.25 g by first dissolving/suspending each organic Ser* and Pser* additive in 60 μL of deionized water (see Table S1), whereupon a powder of α-Ca_3_(PO_4_)_2_ (α-TCP) (see refs (54) and (60) for preparation details) was added at liquid-to-powder (L/P) ratio of 0.24 mL/g. The powder and liquid were mixed with a spatula for ≈30 s. The cement paste was cured at 21 °C and 30% relative humidity for 15–30 min and then transferred to a sealed plastic bag that was stored at 37 °C for 7 days at 100% humidity. All cement specimens were thereafter stored under dry conditions in a desiccator. For practical reasons, pH measurements of the cement pastes prior to their setting were performed on larger Ser*N*/Pser*N* batches of 3.0 g and a slightly higher L/P = 0.4 mL/g ratio. Table S1 presents the pH value associated with each cement preparation, obtained as an average over three independent preparations.

### 2.2 Solid-State NMR Experiments

The solid-state NMR experimentation was performed with Bruker Avance-III spectrometers operating at the magnetic fields (*B*_0_) of 9.4 T or 14.1 T, which provided {^1^H, ^13^C, ^31^P} Larmor frequencies of {−400.1, −100.6, −162.0} MHz and {−600.1, −150.9, −242.9} MHz, respectively, along with 60.4 MHz for ^15^N at 14.1 T. A fine powder of each specimen was packed in a ZrO_2_ rotor of outer diameters of either 2.5 mm (thin-wall), 3.2, or 4 mm, as specified along with each MAS rate (ν_r_) in the figure captions and in Section S1, which provides all details of each NMR experiment. Full rotors were employed throughout, except for the REDOR NMR^39^ experiments, for which each sample was restricted to the center 1/3 volume of a 4 mm rotor. ^1^H/^13^C, ^31^P, and ^15^N chemical shifts are quoted relative to neat tetramethylsilane (TMS), 85% H_3_PO_4_(aq), and solid ^15^NH_4_Cl, respectively.

### 2.3 Metadynamics Simulations

**Figure 1:**
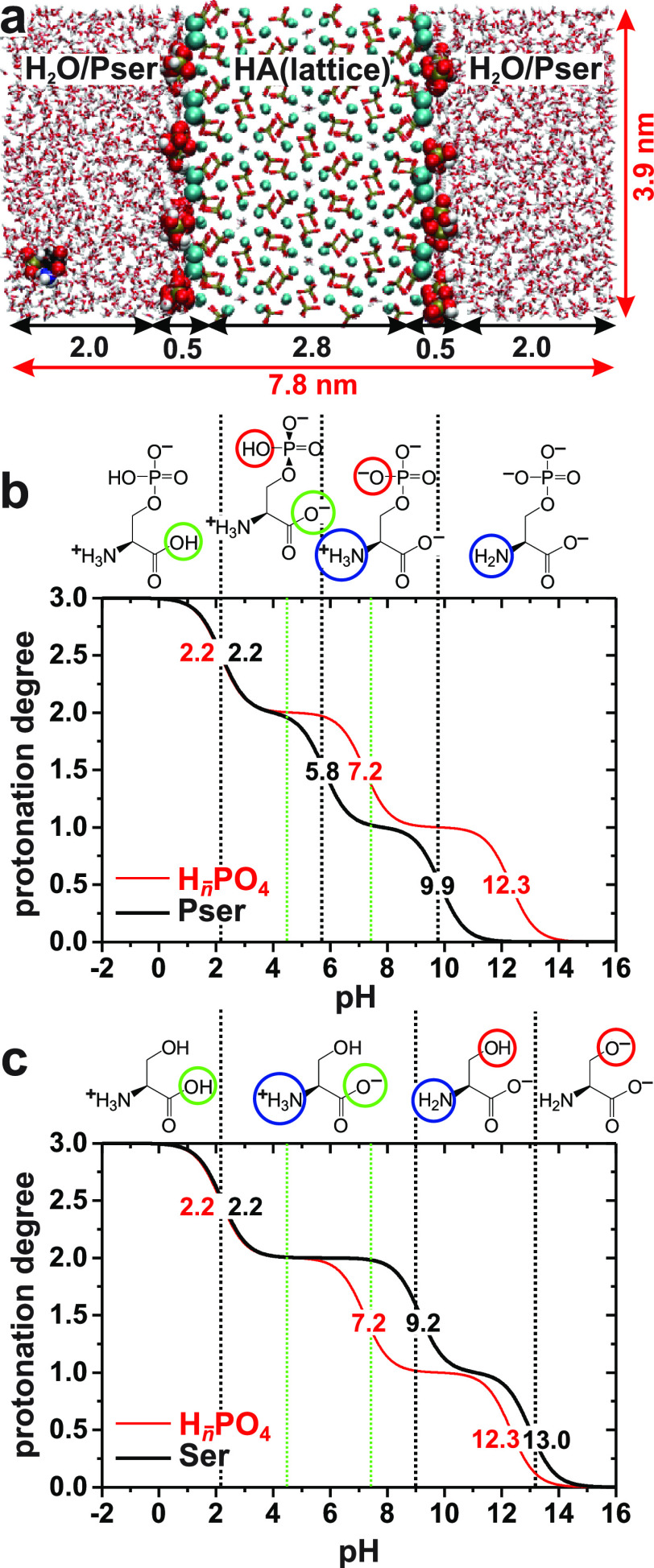
(a) Simulated three-component system, consisting of a “HA slab” with a lattice of crystalline HA and a disordered surface that interface a water phase that contains one Pser molecule (or Ser; not shown). The surface atoms are enlarged for better visualization. The box lengths along the *z* and *x* directions are indicated, along with the (approximate) lengths of the water and lattice/surface domains. (b,c) Dependence of the average number  of protons per phosphate group at the as-prepared HA surface (red curve) along with the net number of protons of the molecular forms of (b) Pser and (c) Ser (black curves) plotted against the pH value of the solution. The most stable molecular form of (b) Pser and (c) Ser at a given pH value is shown on top of each graph, where the colored circles enclose the functional group(s) with altering protonation states between the pH domains marked by black vertical lines. The vertical green lines mark the simulated pH values of 4.5 and 7.4. Each number inside the graph specifies the respective p*K*_a_ values of the organic functional groups, as well as the inorganic {H_3_PO_4_, H_2_PO_4_^−^, and } surface species (the phosphate speciation at the surface matches that of the surrounding solution^45^).

The preparation of the simulated systems and the well-tempered metadynamics simulations were performed as described in ref (48). Here, we only recapitulate the most essential information and refer to Section S2 and ref (48) for details. A box with side-lengths {3.9, 4.3, 7.8} nm (periodic boundary conditions) contained the simulated system of three components (Figure 1a): one Ser or Pser molecule in a water phase (2600 H_2_O molecules) that interfaced a “HA slab”, whose interior consisted of an HA lattice of the biologically relevant *P*6_3_/*m* modification.^66^ The “surface” segment was generated according to ref (45), which mimics a disordered surface at a nanocrystalline HA-particle by introducing acidic protons at randomly selected phosphate groups accompanied by Ca^2+^-cation removal until a charge-neutral surface is obtained, whose {, , } speciation matches that of phosphate ions in an aqueous solution at a given pH value; see Figure 1. The HA surface and the Pser/Ser protonation states were implemented for the experimentally relevant pH values of 4.5 for Pser and 7.4 for Ser, as explained further in Section S2.

The atomistic MD simulations involved *NVT* ensembles at the temperature *T* = 37 °C, utilizing the GROMACS v2018.1 platform^67^ and the following force-fields: INTERFACE^45,49^ for the HA slab, CHARMM36 (July 2017)^68^ for the Pser/Ser molecules, and TIP3P^69^ (without Lennard-Jones terms for H^70^) for water. Well-tempered metadynamics^61,62^ with a bias factor of γ = 5 and 32 independent “walkers”^62,71^ were employed to ensure an efficient sampling of the molecular conformations and accelerate the convergence, using the variational enhanced sampling (VES) protocol^72^ implemented in the PLUMED2.4 software.^73^ Two collective variables were exploited for locating the most stable surface-bound molecular conformation, involving the distance between the center of the HA slab and the **C**OO^–^/ atom of the respective Ser/Pser molecule along with an interaction-energy dependent function defined in Section S2. The reported modeled parameters and their uncertainties are averages over 4 independent simulations.

## 3 Results and Discussion

### 3.1 Local ^31^P Environments Probed by MAS NMR

**Figure 2:**
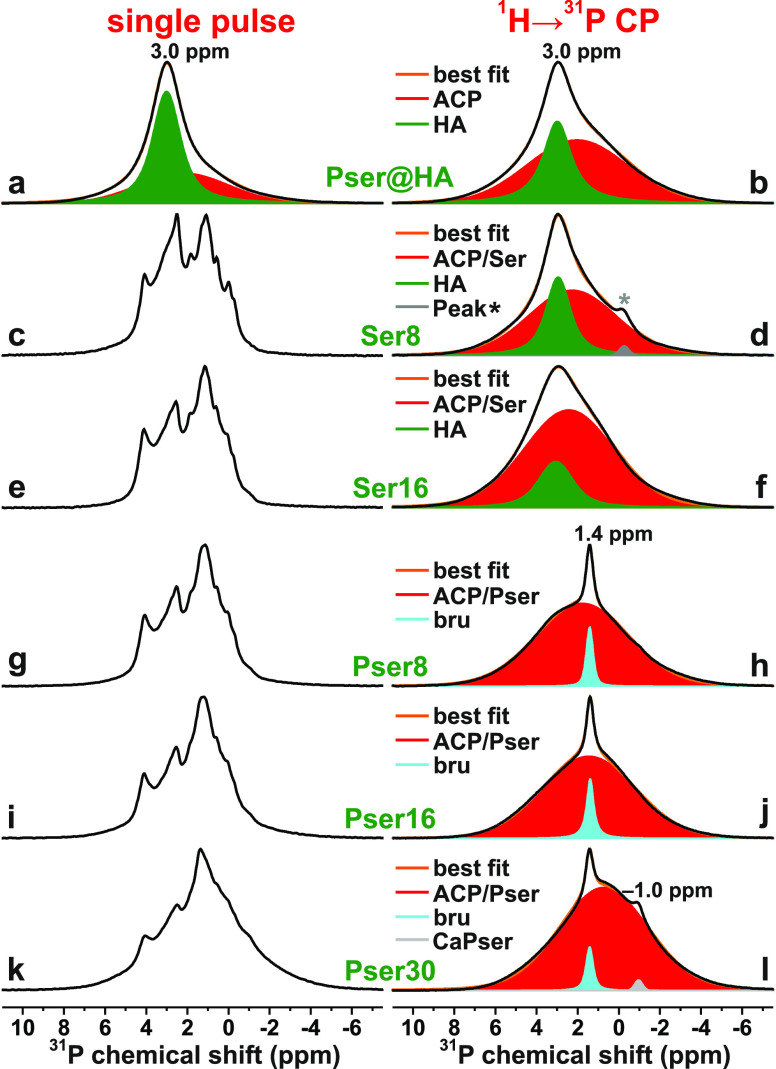
^31^P MAS NMR spectra recorded at 14.00 kHz MAS either directly by single pulses (left panel) or by ^1^H → ^31^P CP (right panel) from (a,b) Pser@HA, or from the as-indicated (c–f) Ser*N* and (g–l) Pser*N* cements. Note that no NMR peak from remnants of the proton-free α-TCP precursor (Figure S4) appear in the CPMAS-derived spectra; the latter are shown together with the best-fit spectra (orange traces) and the deconvolutions into the as-indicated peak components given in each legend. The narrow NMR peaks at 1.4 ppm and −1.0 ppm stem from minor impurities of brushite (CaHPO_4_·2H_2_O) and CaPser (Ca[*O*-phospho-l-serine]·H_2_O), respectively, while that marked by an asterisk in (d) derives from an unknown impurity. Table S6 lists the best-fit NMR parameters.

Figure 2 displays ^31^P MAS NMR spectra recorded from the nanocrystalline Pser@HA sample and the Pser*N*/Ser*N* cements with *N* = {8, 16, 30}. The peak intensities of the single-pulse-acquired NMR spectra of the left panel quantitatively reflect the relative phase constituents of each sample, whereas those of the right panel of Figure 2 were obtained by ^1^H → ^31^P CP, thereby only revealing ^31^P NMR signals from phosphate groups nearby protons. The directly excited ^31^P MAS NMR peak shapes from the cements are complex due to the contributing signals from unreacted α-TCP (Figure S4). The absence of protons in the α-TCP structure, however, renders the ^1^H → ^31^P CPMAS spectra considerably simpler because they only comprise ^31^P resonances from ^1^H-bearing phases, which predominantly involve the amorphous ACP/Pser or ACP/Ser components.^60^ We henceforth focus on the ^1^H → ^31^P CPMAS NMR spectra along with the deconvolutions into their underlying NMR-peak components and the associated best-fit parameters presented in Table S6. The NMR spectra from the cements in Figure 2 overall match those presented in ref (60) from near-identical preparations with ^13^C/^15^N isotopes at natural abundance.

Both the directly excited and CP-derived ^31^P MAS NMR spectra of the Pser@HA specimen (Figure 2a,b) are typical for nanocrystalline HA particles, which involve a crystalline HA “core” coated by a surface layer (“shell”) of ACP.^2−4,12−17^ The “core” and “shell” components produce a narrow and broad resonance, respectively,^4,12−14^ centered at the corresponding ^31^P chemical shifts δ_P_ ≈ 3.0 ppm and δ_P_ ≈ 2.2 ppm (Figure 2a,b and Table S6). The lower shift of the ACP component reflects the acidic solution (pH = 5.3) surrounding the Pser@HA particles before their isolation, which yields a surface layer of ACP enriched in protonated phosphate moieties^4,13,14,16,74^ resonating at lower ^31^P shifts than nonprotonated  groups.^75−77^ Moreover, the close ^1^H–^31^P distances of /H_2_PO_4_^−^ groups emphasize the “ACP” contribution in the ^1^H → ^31^P CPMAS NMR spectrum (Figure 2b) relative to the directly excited counterpart (Figure 2a); also see Table S6.

Cements prepared from α-TCP and water in the absence of Pser consist of disordered HA.^60,78−80^ Although ACP and HA particles are unstable in acidic aqueous solutions, they become stabilized by negatively charged organic additives in *weakly* acidic solutions.^4^ The pH value of the precursor paste before the cement setting is reduced for increasing Pser/Ser content, while at a fixed doping level *N*, Pser yields a markedly lower pH value than Ser (Table S1). Hence, the highly acidic Pser*N* cement pastes preclude HA formation, whereas the Ser8 and Ser16 samples comprise significant HA contributions (Figure 2d,f and Table S6), as proved unambiguously by the heteronuclear correlation NMR results of Section 3.2. The progressively reduced pH values for the Pser-bearing cements also manifest as ^31^P resonance displacements of the ACP/Pser phase toward lower chemical shifts for increasing *N*. These acidic conditions also induced a minor brushite (CaHPO_4_·2H_2_O) formation, as signified by the narrow resonance at δ_P_ = 1.4 ppm^60,81,82^ (Figure 2h,j,l). It is instructive to contrast the ^31^P shifts among the ACP (≈2.2 ppm; Figure 2a,b), ACP/Ser (≈2.3 ppm; Figure 2d,f), and ACP/Pser (0.7–1.8 ppm; Figure 2h,j,l) phases with that from amorphous tricalcium phosphate of approximate stoichiometry Ca_3_(PO_4_)_2_·*n*H_2_O (δ_P_ ≈ 3 ppm)^81,83,84^ and the H^31^ environments of amorphous CaHPO_4_·*n*H_2_O (δ_P_ ≈ 1.5 ppm).^4,77^

### 3.2 Organic/Inorganic Interface Probed by ^1^H–^31^P Correlation NMR

**Figure 3:**
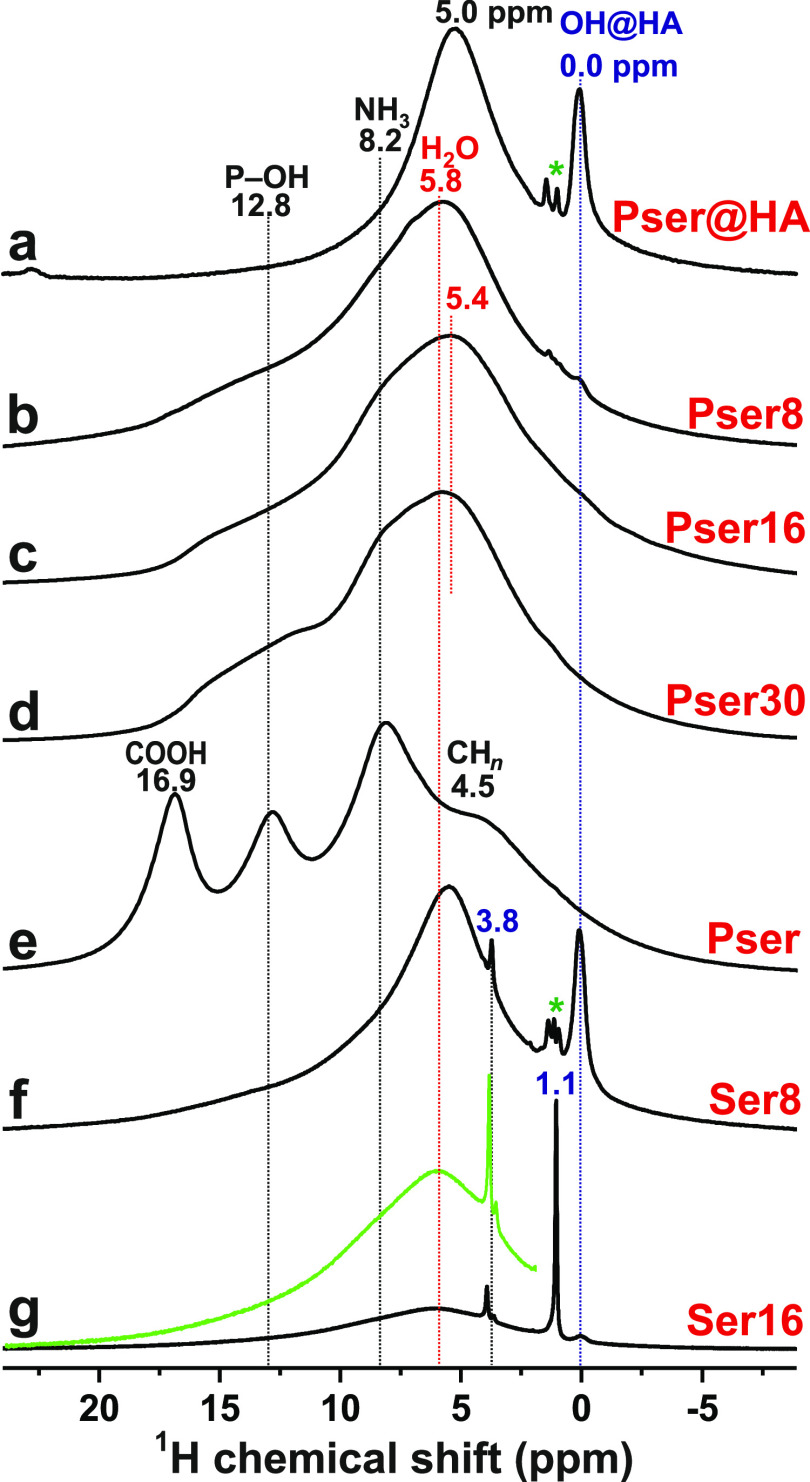
^1^H NMR spectra recorded at 9.4 T and 14.00 kHz MAS from the as-indicated Ser*N* and Pser*N* cements, as well as the Pser@HA and Pser samples. The green trace in (g) is a vertical expansion. The sharp peaks at 3.8/1.1 ppm in (f,g) stem from a minor isopropanol impurity, whereas those marked by asterisks in the NMR spectra from Pser@HA and Ser8 derive from HA-associated surface water molecules, as discussed in detail in refs (60) and (74)

Figure 3 presents the ^1^H MAS NMR spectra recorded from the Pser@HA sample along with the Ser*N* and Pser*N* cements. The various ^1^H sites of the present specimens may be of organic {COOH, POH, NH_3_, CH_*n*_} or inorganic {OH, H_*n*_} origin, along with H_2_O molecules that may either constitute an integral component of the inorganic ACP and HA structures (“structure-bound”), associated with the inorganic/organic ACP/Pser and ACP/Ser components or being mobile physisorbed species. The latter ^1^**H**_2_O molecules typically resonate in the 4.5–5.5 ppm spectral region,^13,14,16,74,85,86^ where Figure 3 suggests that they constitute a significant fraction of the entire proton reservoir in the Pser@HA and Pser*N*/Ser*N* samples. Moreover, the characteristic ^1^H NMR peak at δ_H_ ≈ 0 ppm^12−14,16,74,85,86^ from OH groups in the HA lattice is observed from several specimens. We refer to Mathew *et al.*(60) for discussions on the various ^1^H resonance assignments made from nominally identical Pser*N* and Ser*N* cement compositions.

**Figure 4:**
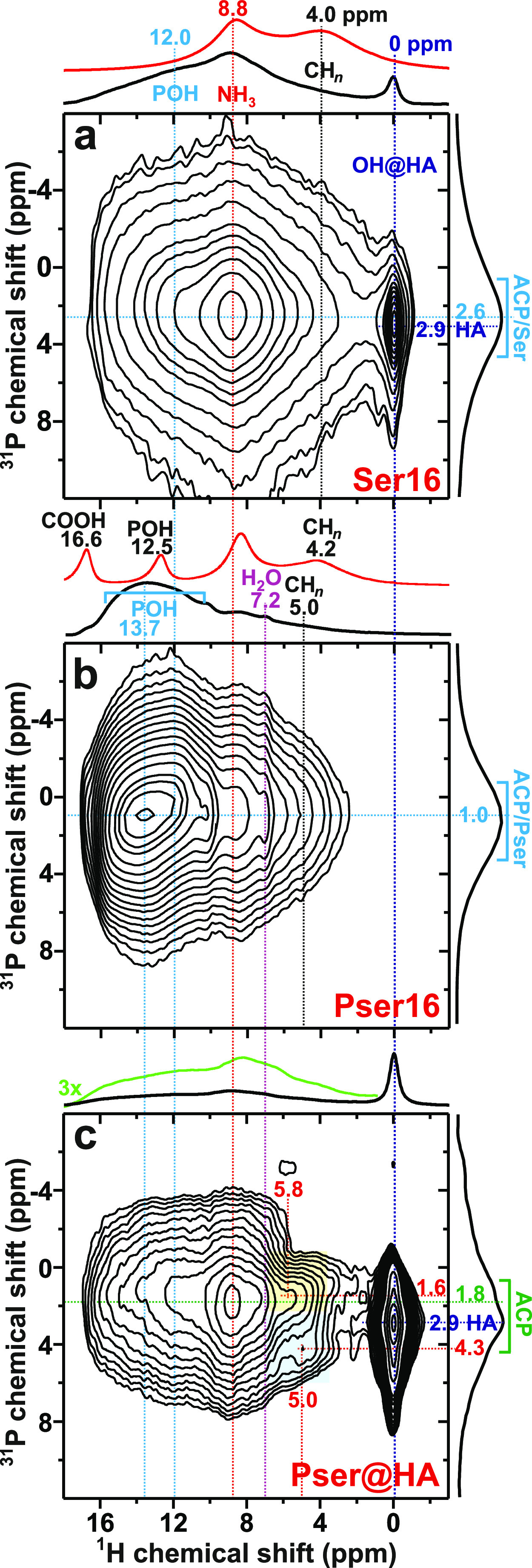
^1^H{^31^P} D-HMQC correlation NMR spectra acquired at 34.00 kHz MAS from the (a) Ser16, (b) Pser16, and (c) Pser@HA specimens using a short HMQC excitation period of 176 μs. The 2D NMR spectra are shown together with projections along the ^1^H (horizontal) and ^31^P (vertical) dimensions at the top and to the right, respectively; the green trace in (c) is a three-fold vertical expansion of the ^1^H projection. Directly excited (“single-pulse”) ^1^H MAS NMR from crystalline Pser and Ser are displayed by red traces in (a,b). Most of the ^1^H resonances marked along the horizontal dimension are of organic origin, whereas the peaks assigned along the ^31^P dimension originate either from HA (blue color) or the ACP/Pser or ACP/Ser cement constituents (cyan color). The 2D NMR regions {δ_P_, δ_H_} ≈ {1.6, 5.8} ppm and {δ_P_, δ_H_} ≈ {4.3, 5.0} ppm marked by the respective yellow and blue rectangles in (c) are discussed in Section 3.2).

The remainder of the article focuses on the probing of the organic/inorganic interface in the Pser@HA, Pser16, and Ser16 specimens, which were examined by dipolar-based MAS NMR experimentation, such as the dipolar-mediated heteronuclear multiple-quantum coherence (D-HMQC) ^1^H{^31^P} NMR spectra^60,82,87,88^ shown in Figure 4. They were recorded from the Pser@HA, Pser16, and Ser16 specimens using a short HMQC excitation period (τ_exc_ = 176 μs) to ensure detection predominantly of ^1^H and ^31^P sites separated by at most a few hundreds of pm. In each D-HMQC spectrum, a 2D NMR correlation peak centered at the chemical-shift pair {δ_1_, δ_2_} ≡ {δ_P_, δ_H_} evidences close spatial proximity between ^1^H and ^31^P sites resonating at the (average) chemical shifts δ_H_ and δ_P_, respectively.

Figure 4a offers direct and unambiguous evidence for an intimate ACP/Ser integration across a sub-nm scale, whereas the Pser16 counterpart reveals HMQC correlations stemming from both intramolecular ^1^H–^31^P dipolar interactions of Pser and Pser···ACP contacts of the ACP/Pser phase (Figure 4b). The ^1^H{^31^P} D-HMQC spectral signatures observed from a Pser*N* cement in the high-δ_H_ region reflect correlations between acidic protons and (in)organic phosphate groups. Hence, it depends strongly on the pH value before cement setting (*i.e.*, on the Pser content; Table S1). Indeed, the higher pH = 5.3 of the solution that immersed the Pser@HA particles renders the 2D NMR spectrum in Figure 4c closer to that reported previously from a Pser4 specimen^60^ than that from Pser16. As expected from the results of Figures 2 and 3, the D-HMQC NMR spectra recorded from the Pser@HA powder (see Figure 4c) and the Ser16 cement (Figure 4a) manifest the HA-characteristic correlation at {δ_P_, δ_H_} = {2.9, 0} ppm.^13,14,16,74,86^

Correlations between the organic protons of the {N**H**_3_, C**H**_*n*_} moieties and / groups of ACP dominate the ^1^H NMR spectral range of δ_H_ ≲ 10 ppm, as becomes evident by contrasting the projection of the 2D NMR spectra of Figure 4a,b along the ^1^H dimension with the single-pulse-acquired ^1^H MAS NMR spectra from the crystalline Pser and Ser samples. The comparatively more intense 2D NMR-peak intensities of the **P**O_4_ contacts with ^1^H shifts ≈9 ppm relative to those of C**H**_*n*_···**P**O_4_ (4–5 ppm) ppm of either cement suggest much shorter distances between the inorganic phosphate species and the positively charged  moiety than the aliphatic groups that do not bond directly to the surface, as indeed predicted by the metadynamics simulations (Section 3.3) and discussed further in Section 3.6.

Moreover, the HMQC NMR spectrum of Pser@HA reveals two broad but resolved 2D NMR peaks (marked by blue/yellow rectangles in Figure 4c) not observed from the cements. They are tentatively attributed to either aliphatic protons or water molecules nearby inorganic phosphate groups, where the 2D correlation centered at {δ_P_, δ_H_} ≈ {1.6, 5.8} ppm and extending toward lower ^31^P and ^1^H chemical shifts likely reflects C**H**_*n*_/**H**_2_O··· proximities. Further work is required to reach firmer assignments, particularly for the {δ_P_, δ_H_} ≈ {4.3, 5.0} ppm correlation, whose high ^31^P shift is atypical of  (and let alone ) groups in CaP phases, although ^1^H–^31^P correlation NMR peaks at similar shifts have been ascribed to  species in the HA lattice.^74^ As discussed further in Section S4, the signal at δ_H_ ≈ 7 ppm in the ^1^H{^31^P} D-HMQC NMR spectra from Pser and Pser@HA is characteristic of structure-bound H_2_O molecules of “ACP”, encompassing the amorphous surface of nanocrystalline HA.^16,60,89^

### 3.3 Surface Binding of Pser and Ser Modeled by Metadynamics

#### 3.3.1 Overview and General Considerations

Metadynamics simulations were utilized to locate the energetically most favorable molecular adsorption at a structurally disordered HA surface representative for the nanocrystalline Pser@HA particles as well as the ACP component of the Pser16/Ser16 cements. The simulations were performed for a pH-dependent HA surface representative of structurally disordered forms of its crystallographic (100) and (001) faces interfacing a water phase comprising one Ser or Pser molecule with protonation states for each of pH = {4.5, 7.4}; see Figure 1 and Section S2.

**Figure 5:**
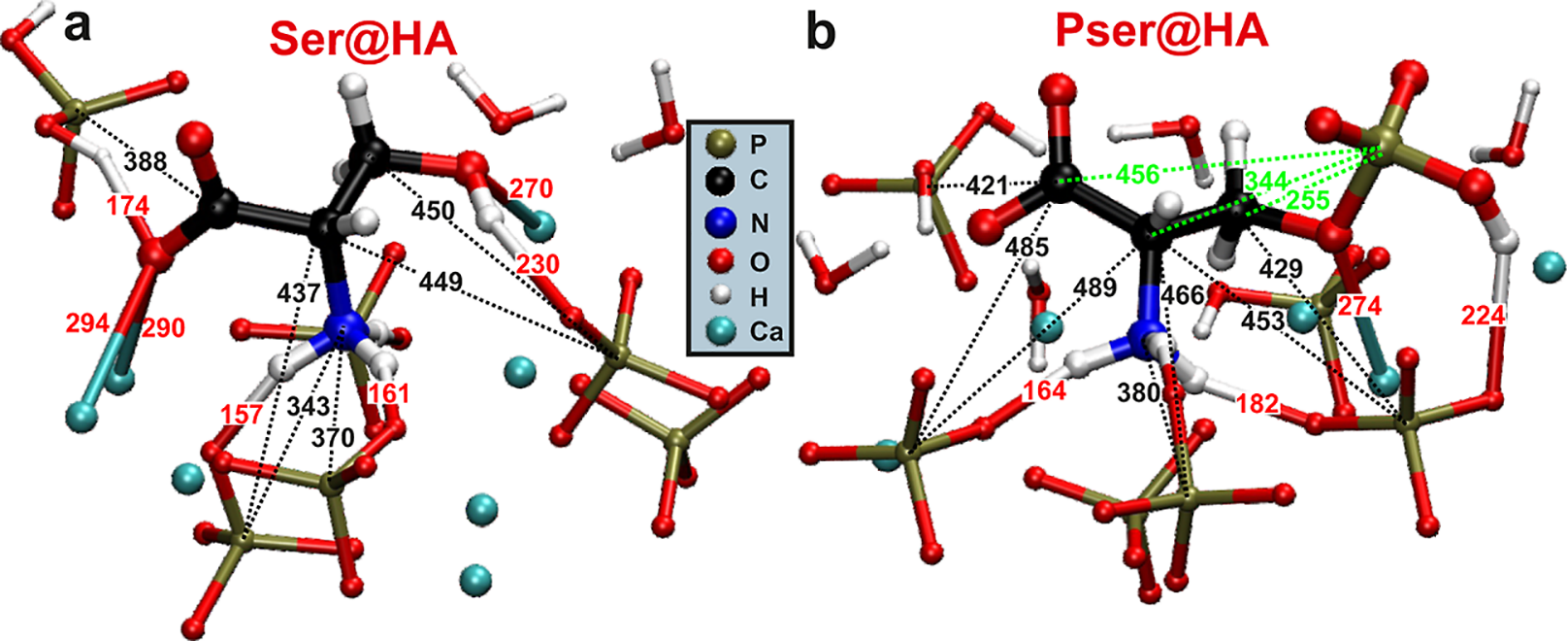
Representative examples of the most stable/probable metadynamics-derived molecular binding modes at a “disordered (100) HA surface” of (a) Ser at pH = 7.4 and (b) Pser at pH = 4.5. All red numbers mark selected distances (in pm) between directly bonded Ser/Pser···HA atoms, while those in black indicate C–P and N–P distances relating to the experimental constraints from NMR. The green numbers in (b) represent intramolecular C–P distances of Pser. For visualization purposes, only a few surface contacts are shown for each organic moiety. Notably, because none of the surface-binding modes of either Ser or Pser involve the sole anchoring of one functional group (Table 1), both molecules assume an extended conformation that “caps” the HA surface. We underscore that owing to the distributions of stable binding modes, no single graphical picture can capture all details of the molecular adsorption (Tables 1, S3, and S4).

Numerous experimental studies on HA nucleation/growth in the presence of both small and large biomolecules suggest a preference for molecular adsorption at the larger (100) or (101) HA surfaces relative to their smaller (001) counterpart,^28,90−93^ as also corroborated by previous modeling.^18,40,43−45,94,95^ Indeed, the consistently stronger adsorption at the “disordered” (100) surface was recently deduced by metadynamics simulations of the Pser binding at HA across a wide pH range of 4.5–14 (ref (48)): Table S3 reproduces the results for the Pser adsorption at each pH = {4.5, 7.4} value and (100)/(001) surface,^48^ along with those obtained herein from Ser. Hence, we focus on the results for (100) at each experimentally relevant pH = 7.4 for Ser16 and pH = 4.5 for Pser16/Pser@HA, whose most probable/representative stable binding modes are exemplified in Figure 5. No aliphatic CH/CH_2_ groups of either molecule are discussed because they do not bind directly at the HA surface.

There are two main groups of *A*···*Bcontact modes* (*i.e.*, “bonding types”) between an organic atom site *A* of Ser/Pser and an inorganic surface-atom *B*: (i) electrostatic (“ion–ion”) interactions among negatively charged O sites of carboxy/phosphate groups and positive Ca^2+^ cations of the ACP layer at nanosized HA particles, and (ii) H bonds between an organic (inorganic) proton and an inorganic (organic) O site. Notably, although carboxy/phosphate groups of biomolecules adsorb *via* both interaction types, each electrostatic bond (*e.g.*, C**O**···**Ca**^2+^) is around 2.5 times stronger than its H-bond counterpart (*e.g.*, C**O**···).^48^ These relative interaction strengths underlie the current consensus that ion–ion interactions dominate the energy landscape of biomolecular adsorption,^18−21^ notwithstanding that the H bonds (140–220 pm) are shorter than the **O**···**Ca**^2+^ distances (240–330 pm); see Figure 5.

#### 3.3.2 Distinct Biomolecular Binding at (100) and (001) HA Faces

Our comments made in Section S2.5 about biomolecules interacting with inorganic species at an essentially *amorphous* HA surface might seem to preclude any preferential adsorption at a specific HA surface type. This apparent contradiction, however, may be reconciled by noting that the consistently stronger adsorption at the “(100)” surface does not stem primarily from crystallographic/structural features—where the (*hkl*) notation rather specifies the *crystallographic origin* of the disordered (100) and (001) surfaces—but merely from its higher molar ratio *n*_Ca_/*n*_P_ ≈ 1.55 relative to that of *n*_Ca_/*n*_P_ ≈ 1.15 for (001) across the pH-range of 3.8–7.4. Consequently, the comparatively higher Ca^2+^ abundance (and larger *n*_Ca_/*n*_P_ ratio) at the disordered (100) surface relative to its (001) counterpart emphasizes the electrostatic C**O/**P**O**···**Ca**^2+^ interactions that predominantly govern the net biomolecular adsorption energy,^18−21,48^ as confirmed by Table S3.

The above-quoted *n*_Ca_/*n*_P_ values were estimated from the chemical speciations of the *two outermost* layers at each (100) and (001) surface at the end of the metadynamics MD simulation (≈40 ns); see Table 1 of ref (48) for the corresponding precise speciations at the *outermost* surface layer relevant for the pH values of the present study. Notably, due to minor ion-dissolution processes, the exact Ca^2+^/phosphate surface speciations vary slightly throughout each metadynamics simulation, thereby not necessarily being identical to those plotted in Figure 1b,c that represent the initially prepared outermost surface layer; see ref (48) for details.

**Table 1:** MD-Derived Surface-Adsorption Data^a^

	Pser		Ser	
pH	**4.5**	7.4	4.5	**7.4**
Δ*F*_ads_	–**40.7**	–66.4	–36.6	–**37.7**
Contributions to Binding Energy^b^				
phosphate	**0.72**	0.70		
carboxy	**0.20**	0.26	0.57	**0.64**
amino	**0.08**	0.04	0.30	**0.16**
hydroxyl			0.13	**0.10**
Binding Mode Statistics^c^				
PN	**0.51**	0.15		
PC	**0.23**			
PCN	**0.15**	0.74		
CN			0.41	**0.30**
CNO			0.55	**0.59**

a Metadynamics-derived Helmholtz free energy of adsorption (Δ*F*_ads_) for the Pser and Ser binding at the disordered (100) surface for pH = {4.5, 7.4}; see Section S2. A more negative Δ*F*_ads_ value implies a stronger surface binding. The results representative for the Pser@HA/Pser16 (pH = 4.5) and Ser16 samples (pH = 7.4) are typeset in boldface. The data for Pser are reproduced from ref (48).

b Fractional contribution of each functional group to the net adsorption energy, as defined in Section S3. No aliphatic group is surface bound.

c Distribution of stable binding modes, where each number reflects the probability (relative fraction/contribution) out of all stable binding modes. The capital letters P, C, N, and O represent the phosphate (Pser), carboxy, amino, and hydroxyl (Ser) group contributions, respectively. Only binding modes with probability ≥ 0.10 are listed.

#### 3.3.3 Ser and Pser Binding Modes at HA/ACP

Figure 5a exemplifies one of the most probable/representative Ser binding modes at ACP in solutions at physiological pH = 7.4, relevant both for the ACP/Ser component of the Ser*N* cements and the amorphous surface at HA nanoparticles, all of which involve simultaneous {COO^–^, , OH} anchoring; they account for ≈60% of all stable Ser binding modes, with the remaining constituting a dual carboxy/amino binding (Table 1). The absence of any significant binding mode by one functional group alone leads to a near-parallel “capping” of the Ser molecule along the ACP surface, as illustrated in Figure 5a. Although typically all three {COO^–^, , OH} moieties bind at the HA surface, Table 1 reveals strikingly different relative contributions of {64, 16, 10}% among the respective groups toward stabilizing/driving the Ser adsorption. The overwhelming carboxy-group contribution stems from its prevalent contact mode of strong electrostatic C**O**···**Ca**^2+^ interactions (Table S3), whereas the modest net contribution of ≈26% to the total adsorption energy from the  and OH groups together reflect their primary (for the amino group exclusive) contact mode of weaker H bonds.

In neutral and alkaline solutions, Pser preferentially anchors at the ACP surface by all three {, COO^–^, } moieties,^48^ leading to a significantly more negative adsorption energy than that for Ser (Table 1). The stronger binding originates primarily from the organic phosphate group of Pser, which is the main adsorption promoter and involved in all stable binding modes regardless of the precise pH and (100)/(001) surface type;^48^ see Table S3. However, the Pser–ACP binding weakens significantly in the acidic solutions relevant for the Pser16 and Pser@HA sample preparation conditions (3.9 ≤ pH ≤ 5.3). This feature may be traced to a lower amount of Ca^2+^ cations at the CaP surface and thereby fewer C**O**···**Ca**^2+^ and P**O**···**Ca**^2+^ electrostatic contacts, where the latter are diminished further because they are superseded by weaker PO**H**···H_*n*_P**O**_4_ bonds (Table S4) accompanying the onset of protonation of the organic phosphate group for pH  6.8 (Figure 1). These effects combine into a significantly weakened Pser adsorption at pH = 4.5 (Table 1), which incidentally nearly matches that of Ser at the comparatively Ca-richer HA surface at pH = 7.4 that promotes electrostatic interactions.

Relative to the Pser surface immobilization at pH = 7.4, the weaker Pser···ACP contacts in acidic solutions are reflected in a larger distribution of distinct binding modes at pH = 4.5 (Table 1), along with an overall more modest COO^–^ participation: roughly half of all surface-bound Pser molecules anchor by a dual binding of their phosphate and amino groups, as depicted by Figure 5b, whereas all other binding constellations occur either by the phosphate/carboxy moieties (≈23%) or by all three groups together (≈15%); see Table 1. (We remind that all binding modes not listed in Table 1 are insignificant, such as the anchoring of one functional group alone of either molecule). Yet, although the  and COO^–^ moieties participate in ≈90% and ≈40% of all binding modes, respectively, Table S3 reveals that their corresponding *net energy* contributions to stabilizing the adsorption only amount to ≈8% () and ≈20% (COO^–^) due to the higher C**O**···**Ca**^2+^ interaction-energy per bond relative to that of N**H**···P**O** and the overall dominant P**O**···**Ca**^2+^ interactions.

Out of all functional groups of Pser, the simulations predict the strongest binding at the ACP surface by the phosphate group. However, the broad ^31^P NMR peak from the ACP/Pser phase (Figures 2 and 4) cannot discriminate between the organic and inorganic phosphate contributions, which underscores the very intimate Pser···ACP contacts in the Pser/ACP phase across a sub-nm scale. Indeed, NMR experiments sensitive to the ^31^P–^31^P distance distributions revealed essentially equal average distances in synthetic/pristine ACP and the ACP/Pser components in Pser*N* cements.^60^

### 3.4 ^13^C–^31^P Correlation NMR Reveals Ser/Pser–ACP Contacts

**Figure 6:**
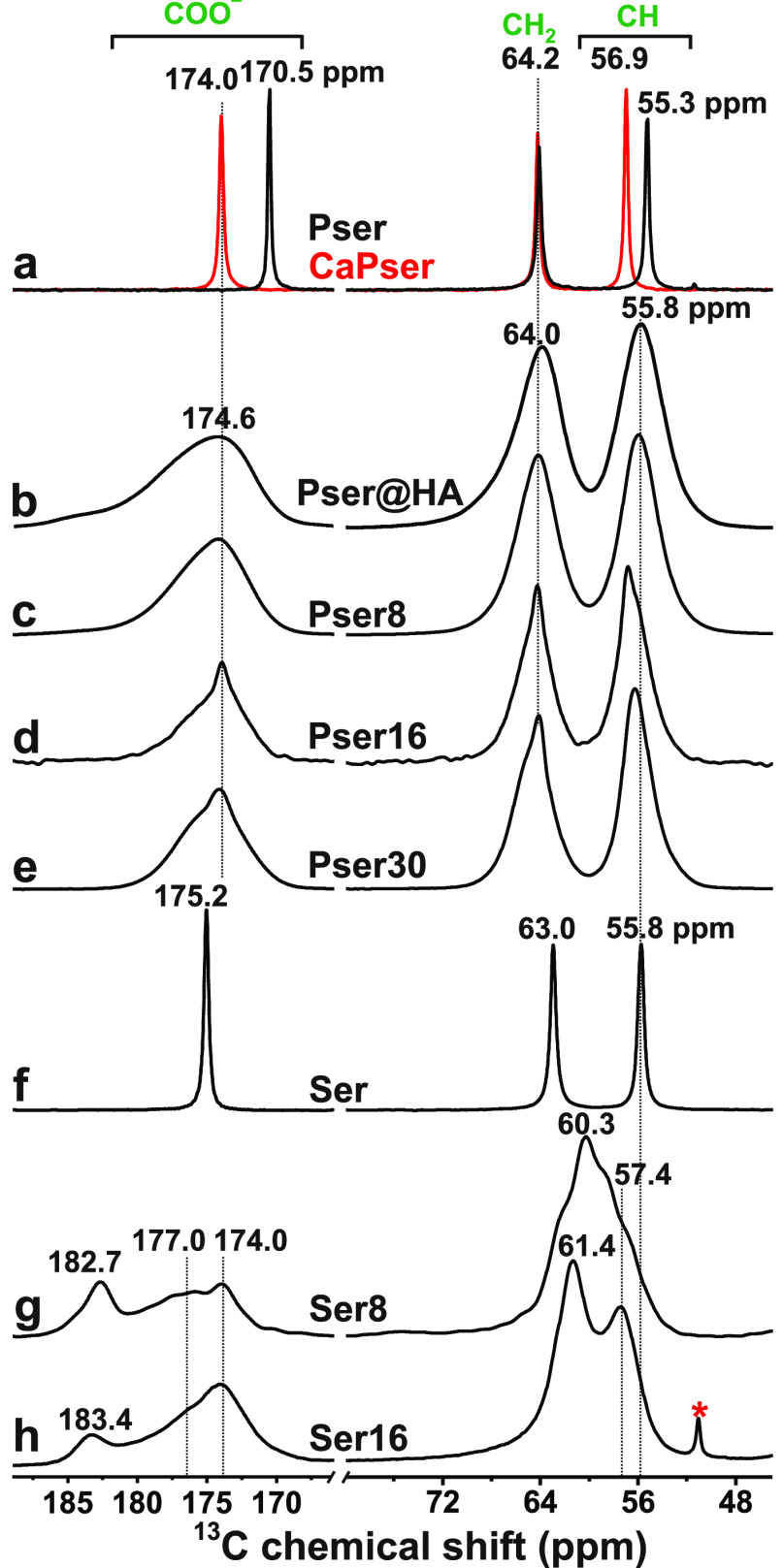
^13^C CPMAS NMR spectra obtained at 9.4 T and 9.00 kHz MAS from (a) polycrystalline Pser (black trace) and CaPser (red trace), (b) Pser@HA, and as-indicated (c–e) Pser*N* cements, as well as (f) polycrystalline Ser along with (g,h) Ser*N* cements. All NMR spectra were recorded from specimens prepared from ^13^C-enriched precursors, except for those shown in (a,f). The asterisk in (h) marks a minor peak from an unknown impurity.

Figure 6 presents ^1^H → ^13^C CPMAS spectra observed from the polycrystalline Ser, Pser, and CaPser powders along with that of the nanocrystalline Pser@HA specimen and the Pser*N*/Ser*N* cements with *N* = {8, 16, 30} prepared from the Pser*/Ser* precursors. The strikingly different ^13^C NMR peak widths observed from the precursors relative to Pser@HA or any cement reflect the distinctly different local order of the ^13^C environments. Hence, even routine ^13^C CPMAS NMR experiments give qualitative evidence for organic/inorganic contacts. Although heteronuclear ^17^O–^43^Ca NMR experiments would enable the most direct probing of the electrostatic C**O**/P**O**···**Ca**^2+^ interactions that dominate the molecular adsorption, such experiments are precluded for NMR-signal sensitivity reasons along with further sample preparation obstacles which require isotopic ^17^O and ^43^Ca labeling.^96−98^ Consequently, we resorted to less challenging ^13^C–^31^P NMR experiments, which merely probe the Pser/Ser···ACP contacts *via* their ^13^C–^31^P distances in ^13^**C**–O···Ca^2+^···O–^31^**P** motifs.

**Figure 7:**
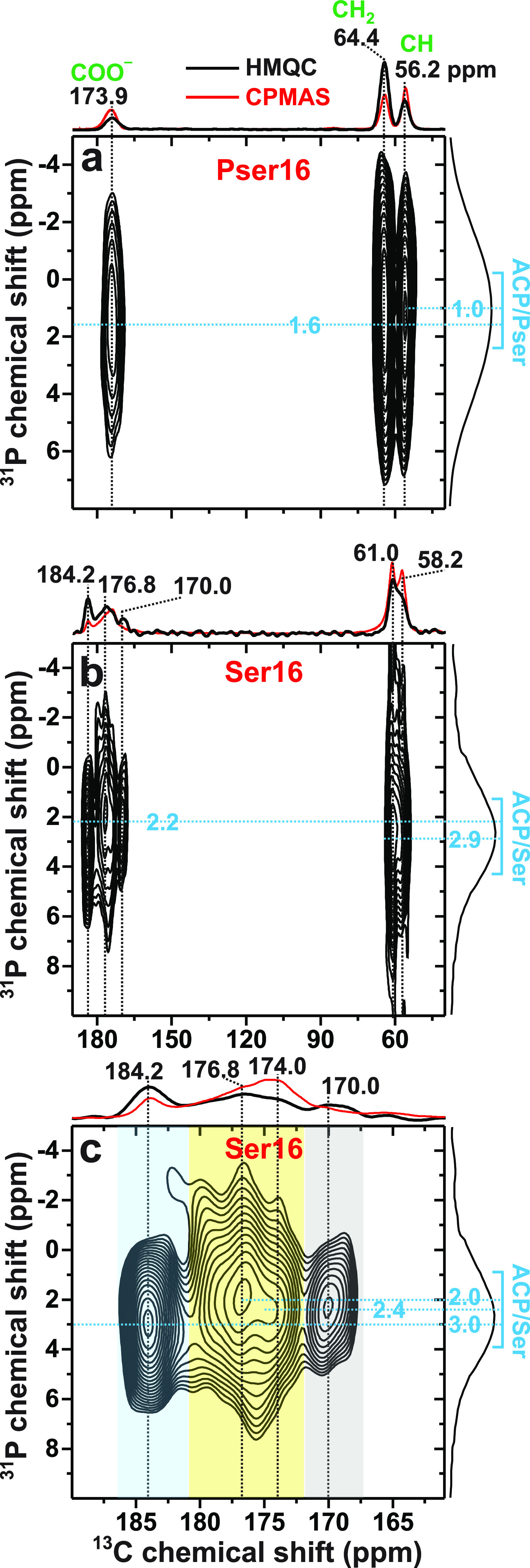
^13^C{^31^P} D-HMQC correlation NMR spectra obtained at *B*_0_ = 14.1 T and ν_r_ = 24 kHz from (a) Pser16 and (b) Ser16 cements by using a HMQC excitation period of (a) 1.75 ms and (b) 2.0 ms. The 2D NMR spectra are shown together with projections along the ^13^C (horizontal) and ^31^P (vertical) dimensions at the top and to the right, respectively, along with their ^1^H → ^13^C CPMAS NMR spectra acquired under the same conditions. (c) Zoom around the carboxy-resonance region of the 2D NMR spectrum in (b). The rectangles indicate ^13^C–^31^P correlations resolved at the ^13^C chemical shifts of 184 ppm (blue), 177/174 ppm (yellow), and 170 ppm (grey).

Figure 7 displays ^13^C{^31^P} D-HMQC NMR^87,88^ spectra recorded from the Pser16 and Ser16 cements, where each ^13^C–^31^P proximity is revealed by a 2D NMR cross peak centered at the shift-pair {δ_P_, δ_C_}. Owing to the phosphate group of Pser, however, the ^13^C{^31^P} D-HMQC spectrum observed from Pser16 primarily reflects the *intra*molecular ^13^C–^31^P distances, which also account for the observed NMR-intensity increase along ^13^CO ^13^CH ^13^CH_2_ in Figure 7a; this is particularly evident when contrasting the various peak intensities of the HMQC projection with their counterparts of the corresponding ^1^H → ^13^C CPMAS NMR spectrum (Figure S5). The 2D NMR-peak intensities reflect semiquantitatively the relative through-space ^13^C–^31^P distances. The significantly emphasized intensity and the higher ^13^C chemical-shift dispersion of the ^13^**C**OO^–^···^31^**P** correlations from the Ser16 cement relative to the Pser16 counterpart (as is also evident from the ^13^C CPMAS NMR spectra of Figure 6) corroborates the metadynamics predictions (Section 3.3): the carboxy group of Ser anchors directly at the ACP surface, in contrast with (a majority of) the Pser molecules. However, owing to the HMQC-signal buildup across longer distances, cross-peaks associated with *all*^13^C sites are detected for the present τ_exc_ values (Figure 7), despite that none of the CH/CH_2_ groups of either Pser/Ser molecule bind directly to any species of ACP.

The close COO^–^···ACP contacts of the Ser molecules are reflected in emphasized 2D NMR-signal intensities in the zoom around the ^13^COO^–^ region of the 2D NMR spectrum of the Ser16 cement (Figure 7c): four 2D NMR ridges are resolved at the ^13^C chemical shifts {184.2, 176.8, 174.0, 170.0} ppm. Notably, none of them coincides with that of δ_C_ = 175.2 ppm from polycrystalline Ser (Figure 6f). As is most transparent from Figure S5, the NMR peaks around δ_C_ = {184, 170} ppm are markedly emphasized (the latter peak is barely discernible in the CPMAS NMR spectrum), whereas the comparatively reduced intensities between 181 ppm and 172 ppm stem from Ser molecules further away from the inorganic phosphate groups. Its significant ^13^C shift-dispersion reflects a range of similar yet distinct COO^–^··· proximities of weakly bound Ser molecular configurations at the ACP surface (along with some resonances from nonbonded molecules; see Section 3.5.3 and the comments mentioned above).

We onwards focus on the two D-HMQC NMR signals centered at the {δ_P_, δ_C_} shift-pairs of {3.0, 184.2} ppm and {2.4, 170.0} ppm, which are tentatively assigned to COO^–^ moieties that are strongly surface bound predominantly/solely *via* electrostatic C**O**···**Ca**^2+^ interactions and C**O**··· H bonds, respectively. While noting that those two contact modes were predicted by modeling (Section 3.3), the NMR-peak assignments were based on the distinctly different ^13^C and ^31^P chemical shifts involved in each correlation: the high shifts of the {δ_P_, δ_C_} = {3.0, 184.2} ppm signal are well-aligned with COO^–^ moieties nearby  groups (see Sections 3.1 and 3.2), whereas the lower chemical shifts of the {2.4, 170.0} ppm correlation are commensurate with H-bond-mediated ^13^**C**OO^–^··· contacts, whose ^13^C chemical shift incidentally matches that of the ^13^**C**OOH group of polycrystalline Pser that manifests analogous inter molecular COOH··· motifs.^65,99,100^ Notably, Figure 6 reveals a lower NMR-signal intensity at 184 ppm from the Ser16 cement relative to its Ser8 counterpart, suggesting a concurrently reduced number of C**O**···**Ca**^2+^ contacts for increasing batched Ser content, along with earlier and more qualitative findings by Mathew *et al.*(60) and our inferences in Section 3.7.

To our knowledge, the present study provides the first ^1^H/^31^P/^13^C correlation NMR signatures of surface-bound Pser molecules at nanocrystalline HA, noting that the previous ^31^P{^1^H} and ^13^C{^1^H} heteronuclear correlation spectra presented by Wang *et al.*(32) from similar nanocrystalline HA preparations in Pser-bearing solutions gave no convincing evidence for Pser···HA contacts. For instance, the ^13^C NMR features merely suggested a prevalence of Pser in crystalline environments than surface-bound ones, which likely resulted from using a very high [Pser] = 200 mM in the solution.^32^ Likewise, our ^13^C CPMAS spectra recorded from various Pser@HA syntheses with increasing Pser concentrations revealed a dominance of crystalline CaPser in all preparations with [Pser] ≥ 20 mM (Figure S3).

### 3.5 Quantitative Probing of Ser/Pser–ACP Contacts by ^13^C{^31^P} REDOR NMR

#### 3.5.1 NMR-Derived Dipolar Second Moments

**Figure 8:**
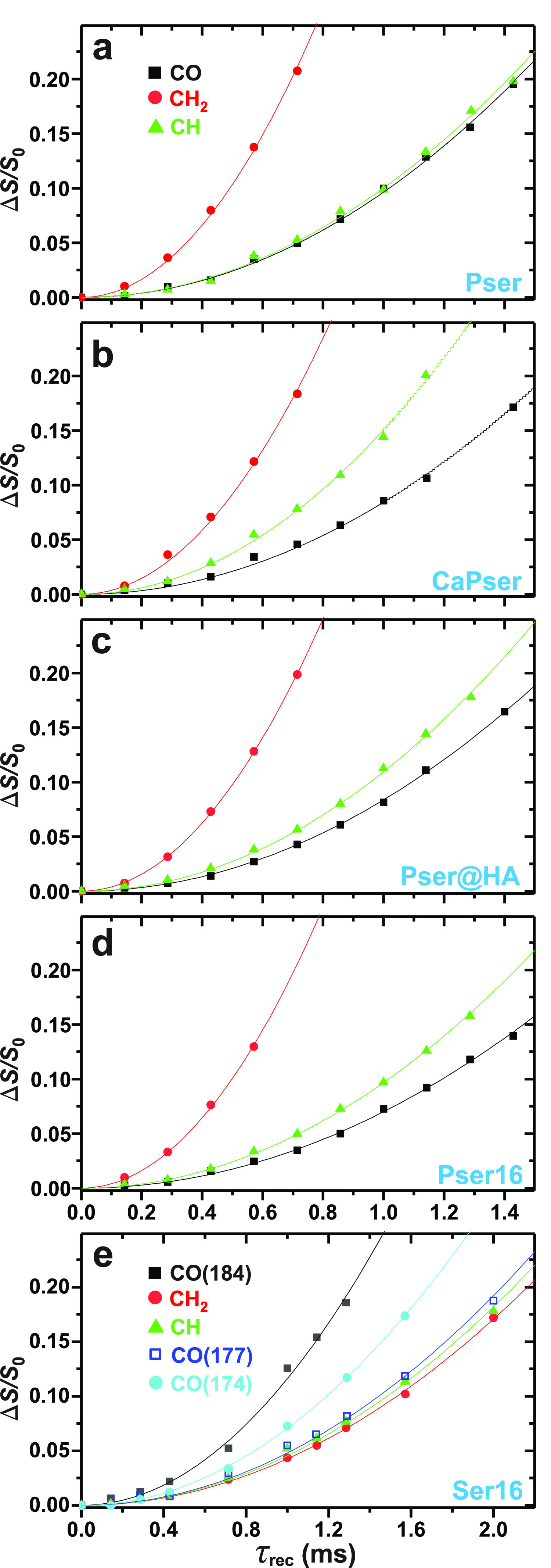
^13^C{^31^P} REDOR NMR dephasing curves (Δ*S*/*S*_0_) recorded at *B*_0_ = 14.1 T and ν_r_ = 10 kHz and plotted for increasing dipolar recoupling/dephasing periods (τ_rec_) for the ^13^CO, ^13^CH, and ^13^CH_2_ functional groups of the (a,b) polycrystalline Pser and CaPser powders (^13^C at natural abundance) along with the ^13^C-enriched (c) Pser@HA, (d) Pser16, and (e) Ser16 specimens. Each curve in (a–e) corresponds to the best fit of the {τ_rec_, Δ*S*/*S*_0_} data to eq S2, which yielded the dipolar second moments {*M*_2_(C_*j*_–P)} listed in Table 2. The ^13^COO^–^ NMR spectral region of the Ser16 sample comprises three distinct resonances at δ_C_ = {184, 177, 174.5} ppm, whose associated dephasing curves are labeled by their chemical shifts in the legend in (e). Note the different horizontal scale in (e) relative to (a–d).

For a quantitative probing of the relative proximities among the {^13^**C**O, ^13^**C**H, ^13^**C**H_2_} sites of Ser/Pser and the inorganic phosphate groups at the HA/ACP surface, we collected the ^13^C{^31^P} REDOR NMR dephasing curves presented in Figure 8. Here, a rapid (slow) dephasing reflects short (long) distances of a given ^13^C_*j*_ site to the inorganic phosphate groups of ACP (with the caveat of *intra*molecular ^13^C_*j*_–^31^P interactions in Pser). Table 2 collects the set of REDOR-derived dipolar second moments^101−106^ {*M*_2_(CO–P), *M*_2_(CH–P), *M*_2_(CH_2_–P)}, extracted by fitting the respective dephasing curves of each sample (Section S1.3). The precise *M*_2_(C_*j*_–P) value depends on the underlying set of interatomic distances {*r*(C_*j*_–P_*k*_)} between a given ^13^C site and its nearby P atoms *via* a sum over [*r*(C_*j*_–P_*k*_)]^−6^ terms. This renders the *shortest* distance contributions most influential for the net *M*_2_(C_*j*_–P) value.

**Table 2:** Experimental and Calculated Dipolar Second Moments^a^

	^15^N–^31^P		^13^C–^31^P					
	*M*_2_(NH_3_–P)/kHz^2^		*M*_2_(CO–P)/kHz^2^		*M*_2_(CH–P)/kHz^2^		*M*_2_(CH_2_–P)/kHz^2^	
sample	exp.	calc.	exp.	calc.	exp.	calc.	exp.	calc.
Ser16	2.7 ± 0.4	4.9 ± 0.7	11.1^b^	14.0^b^	8.9	13.0	7.9	9.6
Pser16^c^	2.8 ± 0.4	5.2 (4.0)±0.7	13.1	13.8 (8.3)	18.2	25.8 (8.9)	75.4	96.8 (5.7)
Pser@HA^c^	4.0 ± 0.4	5.2 (4.0)±0.7	15.7	13.8 (8.3)	20.9	25.8 (8.9)	73.7	96.8 (5.7)
Pser^d^	3.2 ± 0.4	4.5	18.6	24.5	18.9	24.8	79.9	102.5
CaPser		4.6	15.9	20.8	27.8	38.2	71.7	93.4

a ^15^N–^31^P and ^13^C–^31^P dipolar second moments obtained for the as-indicated functional groups from ^15^N{^31^P} and ^13^C{^31^P} REDOR NMR experiments, respectively, and compared with data calculated either from metadynamics-derived structural models (for Ser16, Pser16, and Pser@HA) or from the crystal structures of Pser and CaPser.^65^ The *M*_2_(C_*j*_–P) data uncertainties are ±1.7 kHz^2^ (NMR) and ±2.2 kHz^2^ (metadynamics model), with kHz^2^ ≡ 1000 s^–2^.

b Net experimental/calculated values; the NMR analysis afforded the extraction of three *M*_2_(CO–P) values of {23.6, 9.3, 13.3} kHz^2^ for the ^13^**C**O shifts at {184, 177, 174.5} ppm, respectively.

c Values within parentheses represent the contribution to the net *M*_2_(N–P) and {*M*_2_(C_*j*_–P)} values from the Pser···HA contacts alone; the contribution from the *intra*molecular ^15^N–^31^P and ^13^C_*j*_–^31^P dipolar interactions account for the difference.

d The {*M*_2_(C_*j*_–P)} and *M*_2_(N–P) values were obtained from the Pser (Flamma) and isotopically enriched Pser* samples, respectively.

The NMR-derived {*M*_2_(C_*j*_–P)} data from the polycrystalline Pser/CaPser powders were validated against those calculated from their crystal structures.^64,65,99^ As expected^103−106^ and discussed further in Section S1.3, the NMR-derived dipolar second moments are consistently lower than their theoretical counterparts (). Yet, the  ratios are essentially constant (Table S5): a very good agreement is observed for all *relativeM*_2_(C_*j*_–P) values within each Pser or CaPser structure, which remain well within the experimental uncertainties. For Pser, the experimental and calculated *M*_2_(CO–P):*M*_2_(CH–P):*M*_2_(CH_2_–P) ratios relate as 1.0:1.0:4.3 and 1.0:1.0:4.2, respectively, whereas both data sets for CaPser are 1.0:1.8:4.5.

The markedly different relative *M*_2_(CO–P):*M*_2_(CH–P) values among the Pser and CaPser crystal structures are noteworthy: Despite the long *intra*molecular ^13^**C**O···^31^**P**O_4_ distances in both structures, the comparatively high *M*_2_(CO–P) values (particularly for Pser) originate mainly from multiple *inter*molecular ^13^**C**OOH···^31^**P**O_4_ contacts *via* H bonds.^65,99,100^ Moreover, the markedly different *M*_2_(CH–P) values in Table 2 reflect distinctly different molecular conformations in the CaPser and Pser crystal structures, whose corresponding θ(P–O–C^β^–C^α^) dihedral angles of 280° and 153° translate into intramolecular ^13^**C**H···**P** distances of 341 pm and 384 pm, respectively. Notably, such widely differing Pser conformations are readily discriminated by the ^13^C{^31^P} REDOR NMR experiments. Hence, notwithstanding that each *M*_2_(C_*j*_–P) value is dominated by the intramolecular ^13^C_*j*_–^31^P distance for all scenarios where intermolecular Pser···Pser contacts are negligible (thereby obscuring the herein targeted C_*j*_···ACP contacts), the *M*_2_(CH–P) parameter offers a valuable constraint on the conformation of the surface-bound Pser molecules, such as in the Pser@HA and Pser16 systems.

#### 3.5.2 Validation of the Metadynamics-Derived Dipolar Second Moments

The dipolar second moment *M*_2_(C_*j*_–P) reflects all {^13^C_*j*_–^31^P_*k*_} distances of the adsorbed molecule to its adjacent inorganic phosphate groups (Section S1.3), but cannot unveil the precise underlying distance distribution. However, because the metadynamics simulations do offer such atomistic details (Figure 5), we first validated the modeled *M*_2_(C_*j*_–P) data against the experimental counterparts.

Considering that all REDOR-derived *M*_2_(C_*j*_–P) values are underestimated by  (Table S5 and Section S1.3), a “perfect match” between the metadynamics-generated models and the experiments should result in  for each ^13^C_*j*_–^31^P contact. Notwithstanding a somewhat larger scatter in the ratios of Table S5 (which stems from higher data uncertainties of the simulated systems than the very accurate C/P atom coordinates of the Pser/CaPser crystal structures^64,99^), it is gratifying that the data in Tables 2 and S5 confirmed our expectations for all ^13^C_*j*_–^31^P pairs of *both* Ser/Pser molecules, except for the modeled *M*_2_(CO–P) values relative to the experimental counterparts of Pser@HA and Pser16 that yielded . The somewhat stronger COO^–^···ACP contacts in both Pser@HA and Pser16 specimens than those predicted by the metadynamics simulations imply that the carboxy group contributes more to the Pser binding than that suggested by the binding mode statistics of Table 1. However, besides noting that the phosphate/amino binding-mode population is presumably slightly overestimated at the expense of (primarily) the phosphate/carboxy/amino counterpart, more quantitative corrections cannot be made. We remind that the ^13^C{^31^P} D-HMQC NMR spectra also suggested weaker COO^–^···ACP interactions of the Pser molecules than those of Ser (Section 3.4).

#### 3.5.3 Discussion

The NMR-derived dipolar second moments of the aliphatic groups of Ser (Table 2) suggest *M*_2_(C_*j*_–P)  kHz^2^ as the marker of an *absence* of direct surface binding of any atom of a given ^13^C_*j*_ functional group at the ACP surface. We remind that regardless of the (non)adsorption of Pser at ACP, the intramolecular ^13^C_*j*_–^31^P dipolar interactions render all *M*_2_(C_*j*_–P) values of the Pser@HA and Pser16 specimens markedly higher than those from Ser16, as is evident from the dephasing curves of Figure 8. However, the simulation-derived model enables a separation of the intra/intermolecular contributions to the net *M*_2_(C_*j*_–P) value: indeed, Table 2 confirms the expectation that both CH/CH_2_ groups of Pser exhibit very low dipolar second moments once their intramolecular contributions are excluded (particularly that of *M*_2_(CH_2_–P), for which the organic phosphate group accounts for ≈95% of the net value).

It is gratifying that each of the three {*M*_2_(CO–P), *M*_2_(CH–P), *M*_2_(CH_2_–P)} NMR-derived data agrees mutually very well among the Pser@HA and Pser16 samples, which suggests overall (very) similar Pser contacts/distances to the inorganic phosphate moieties. Hence, despite the *formally* distinct nature of the ACP/Pser phase of the biomedical Pser*N* cements and the Pser adsorption at a nanocrystalline “HA” surface (Pser@HA), the local structure of their organic/inorganic interfaces must be similar in both specimens, thereby consolidating the current consensus that nanocrystalline HA is coated by a layer of “ACP”^4,12−16^ (although its precise chemical/structural nature remains unknown). This aspect also justifies that the single metadynamics simulation at pH = 4.5 mimics well the Pser···ACP interactions in both Pser@HA and Pser16 specimens, as well as supporting the physical relevance of the herein employed HA-surface preparation procedure.^45^ These important issues are discussed further in Section S2.5.

We now return to the partially overlapping ^13^C NMR peaks in the carboxy domain of the NMR spectrum from the Ser16 cement (Figure 6h), which, according to their distinct ^13^**C**OO^–^···ACP contacts, were grouped into three regions in the ^13^C{^31^P} D-HMQC spectrum of Figure 7c. As expected from the δ_C_ = 184 ppm resonance attributed to C**O**···**Ca**^2+^ motifs (Section 3.4), its very rapid dipolar dephasing is consistent with a sizable dipolar second moment of 23.6 kHz^2^ (Table 2). In contrast, the spectral region marked by a yellow rectangle in Figure 7c and deconvoluted into two peak components around 177/174.5 ppm reflects more weakly surface-bound COO^–^ groups with lower dipolar second moments of 9.3/13.3 kHz^2^ (Figure 8 and Table 2). Although a large *M*_2_(CO–P) value is also anticipated from COO^–^ moieties surface bound *via* H bonds and giving an NMR peak at 170 ppm, its minor NMR intensity (and thereby very small population) did not permit reliable analyses of the REDOR NMR data (*e.g.*, see Figure S6). Altogether, the varying dipolar second moments of these COO^–^···ACP contact modes/distances underlie the average value of *M*_2_(CO–P) ≈ 11.1 kHz^2^ (Table 2), which qualitatively corroborates the metadynamics predictions of direct COO^–^···ACP bonds.

We contrasted the D-HMQC NMR-derived and modeled relative populations of the three types of surface-bound COO^–^ sites that produce the three spectral regions marked in Figure 7c and attributed to COO^–^ sites bound solely by Ca^2+^ cations (≈184 ppm), sites weakly bound by both electrostatic and H-bond interactions (181–172 ppm), and solely by H bonds (≈170 ppm). Quantitative agreements are not expected because the 2D NMR intensities depend strongly on the precise HMQC excitation/reconversion periods. Deconvolution of the ^13^C projection of the D-HMQC spectrum (results not shown) yielded fractional populations of {0.27, 0.59, 0.14} for the respective {184, 181–172, 170} ppm resonance regions, whereas the corresponding metadynamics-simulation-derived fractions are {0.68, 0.27, 0.05}. While both experimental and modeled results accorded qualitatively well for the overall most sparse contact mode of solely C**O**···**H**PO_4_^2−^ interactions, the main discrepancy concerns the strong dominance of C**O**^–^···**Ca**^2+^ interactions predicted by the model (68% of all direct surface contacts and consistent with the results of Table S4) compared to the much lower estimate by NMR (27%). The differences presumably stem from the difficulties by NMR to accurately quantify the contributions from the 181–172 ppm resonance-region stemming from “weakly bound” carboxy groups, which may involve non-negligible ^13^**C**OO^–^ NMR signals from more distant nonbonded molecules that are not accounted for in the simulation analysis.

### 3.6 ^15^N{^31^P} REDOR NMR Reveals the Amino-Group Binding

**Figure 9:**
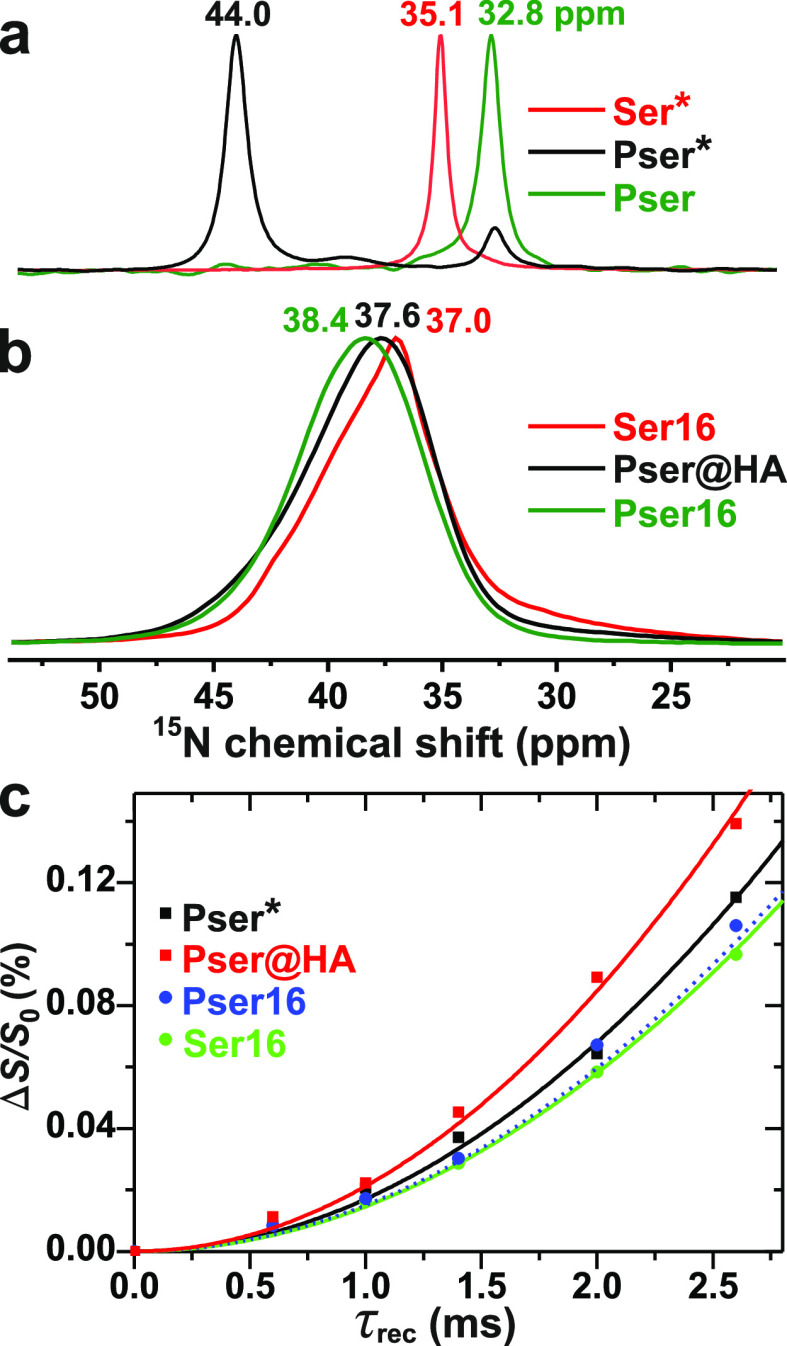
^1^H → ^15^N CPMAS NMR spectra recorded from powders of (a) Pser (^15^N at natural abundance) along with the ^13^C/^15^N-enriched Ser* and Pser* precursors, and their (b) Pser@HA, Pser16, and Ser16 products. The NMR peaks at 32.8 ppm and 44.0 ppm observed from the Pser* specimen are attributed to the ^15^N sites of ^13^C/^15^N-enriched *O*-phospho-l-serine and its HCl salt, respectively. (c) ^15^N{^31^P} REDOR NMR dephasing curves recorded from the as-indicated samples. All NMR results were obtained at *B*_0_ = 14.1 T and ν_r_ = 10 kHz.

Figure 9a,b displays the ^1^H → ^15^N CPMAS NMR spectra of Pser, the ^15^N-enriched Ser* and Pser* precursor powders, together with those of Pser@HA, Pser16, and Ser16. As for the ^13^C NMR spectra (Figure 6), the Ser/Pser adsorption is mirrored by a significant ^15^N resonance broadening along with a minor increase in the average chemical shift of ≈2 ppm and ≈5 ppm for the Ser and Pser bearing specimens, respectively. The amino-group binding at ACP was probed by ^15^N{^31^P} REDOR NMR experiments on the Pser@HA, Pser16, and Ser16 cements, whose dipolar dephasing curves are displayed in Figure 9c along with that from the Pser* powder. For the latter, Table S5 reveals a ratio of 0.71 between the NMR-derived *M*_2_(N–P) value and that calculated from the Pser crystal structure,^99^ which is close to the expected ratio of 0.76 (Section 3.5.2). Moreover, the ratio of 0.77 between the experimental and modeled *M*_2_(P–N) data for the Pser@HA sample suggests a very faithful metadynamics modeling of its ACP/HA contacts (Figure 5b).

Particularly, when recalling the very good mutual match between the {*M*_2_(C_*j*_–P)} sets among the Pser@HA and Pser16 specimens (Section 3.5), the significantly lower *M*_2_(N–P) value of the Pser16 cement (as well as for Ser16) relative to Pser@HA is surprising (Table 2). It suggests much weaker ACP contacts in both Pser/ACP and Ser/ACP cement components than the counterparts of the Pser@HA particles (also see Section S1.3). Although the NMR-derived dipolar second moments from the cements also suggest longer ACP distances than the metadynamics predictions, the qualitative feature of near-equal *M*_2_(N–P) values of the models for both Ser/Pser molecules is supported by the REDOR NMR experiments on the Pser16 and Ser16 samples.

The amino group binds to the ACP surface exclusively *via* H bonds to the negatively charged O atoms of the phosphate groups. Table S4 reveals that the N**H**··· contact mode dominates for Pser (pH = 4.5), whereas the amino group of Ser (pH = 7.4) forms an equal number of bonds to  and  moieties. For the pH conditions of the Pser16 and Ser16 cements, the binding-energy contribution from the amino group is higher for Ser than Pser (Table S3), which also accords with the dipolar second moments of Table 2: although both the experimental and modeled *M*_2_(N–P) values for Pser16 are slightly higher than their Ser counterparts, once excluding the intramolecular ^15^N–^31^P Pser contribution, the expectation of a stronger surface binding of the  group of Ser is confirmed by its  higher modeled *M*_2_(N–P) value than that of Pser.

### 3.7 Implications for Bone-Adhesive Properties

We recently reported a strong correlation between the amount of the amorphous ACP/Pser component and the measured shear strength of Pser*N* cements used for gluing two cubes of either cortical bone^54^ or steel^60^ together across a wide composition range up to *N* = 87 mol %.^60^ The shear strength reflects the bone-adhesive properties.^54^ Notably, both parameters exhibit a nonmonotonic trend against the batched Pser content, with an initial increase up to a plateau of near-constant shear-strengths and ACP/Pser contents in cements incorporating 40–60 mol % Pser, followed by their concurrent decrease for increasing *N* because the bone-adhesive-promoting ACP/Pser component becomes gradually replaced by crystalline CaPser and unreacted Pser.^60^ In contrast, Ser*N* cements form an amorphous ACP/Ser phase that only develops with the batched Ser content up to *N* ≲ 16 mol % (which is insufficient for giving a high shear strength), whereas all remaining Ser remains unreacted.^60^

Consequently, the formation of an amorphous component featuring *both* significant organic–organic and organic–inorganic interactions appears to be a prerequisite for favorable bone-adhesive properties of an α-TCP-derived cement. Hence, the results herein combined with those of ref (60) suggest that a significant bone-adhesion *cannot* stem from the adsorption strength of a given biomolecule at ACP *alone*, because the Pser···ACP binding energy at pH = 4.5 essentially matches with that of Ser···ACP at pH = 7.4 (Table 1), implying that both Pser16/Ser16 cements feature similar Pser···ACP and Ser···ACP net interaction strengths. Nonetheless, while both Pser and Ser molecules may enter ACP/Pser and ACP/Ser phases in the respective Pser*N* and Ser*N* specimens with *N* ≲ 16 mol %, their amounts develop very differently upon increasing organic content (*vide supra*). Consequently, the main distinction between the Pser*N* and Ser*N* cements concerns their respective degrees of Pser···Pser and Ser···Ser contacts.

Due to its low Pser content ( wt %), the Pser@HA sample is expected to involve essentially “isolated” surface-bound Pser molecules with long Pser···Pser distances. Likewise, the metadynamics modeling involved one sole Pser (or Ser) molecule. Hence, the good agreement between the *M*_2_(C_*j*_–P) NMR results of the Pser@HA and Pser16 samples, as well as with the modeled data, suggest that 16 mol % of either Pser or Ser may be sufficiently low to be dispersed within the ACP matrix without any significant Pser···Pser or Ser···Ser aggregation (*i.e.*, analogously with a “monolayer” adsorption scenario). However, the substantial Pser contribution to the ACP/Pser cement component in all Pser-rich Pser*N* cements—for which the shear strength is maximal—cannot be reconciled with a monolayer Pser adsorption at ACP, but must involve a significant Pser···Pser aggregation, yet with the molecules remaining intimately integrated also with the inorganic species of ACP (see comments in Section 3.3.3 and ref (60)). Although further work is required for a definite proof, we propose that the high bone-adhesion/shear-strength of the Pser*N* cements with 40 ≲ *N* ≲ 60 mol % stems from a “stickiness” accompanying the high organic content of their ACP/Pser component, in conjunction with its dominance of the entire cement constitution. Naturally, the “stickiness” is low in cements with batched Pser contents ≲ 20 mol %, as well as for all Ser*N* specimens^60^ due to their insignificant tendency of Ser···Ser aggregation.

## 4 Conclusions

We have presented the first atomistic probing of the Pser and Ser binding at structurally disordered CaP surfaces by a synergistic combination of advanced solid-state NMR experimentation and metadynamics MD simulations, revealing the relative proximities of each molecular functional group and their respective underlying types of bonds, as well as the conformation of the adsorbed molecules. Our study encompassed the organic/inorganic interactions in a sample of surface-bound Pser at nanocrystalline HA particles (Pser@HA) along with two biocements prepared from α-Ca_3_(PO_4_)_2_ doped with 16 mol % of either Pser or Ser. Notably, the very close sets of ^13^C–^31^P dipolar second moments observed from the cement and the Pser@HA sample highlight the similarities of the Pser binding at ACP and the structurally disordered surface layer of nanocrystalline HA, thereby corroborating the current consensus that it is faithfully described as “ACP”.^4,13−16^

The Pser and Ser adsorption is primarily mediated by electrostatic interactions between Ca^2+^ cations and the negatively charged organic COO^–^/ groups and, to a lesser extent, by H bonds to the inorganic phosphate groups, which involves N**H**···P**O**_4_ and C**O**/P**O**···**H**PO_4_ contacts for the amino and carboxy/phosphate groups, respectively. The dominance of electrostatic interactions for driving the adsorption implies that the phosphate group of Pser and the carboxy group of Ser are mainly responsible for stabilizing their surface binding, fully consistent with earlier inferences that biomolecules bind at bone minerals mainly *via* ion–ion interactions.^18−21^

All detailed information about the number of electrostatic/H-bond interactions and the accompanying interatomic distances were extracted from the metadynamics models. They were validated against the NMR-derived interatomic-distance constraints encoded by the dipolar second moments {*M*_2_(CO–P), *M*_2_(CH–P), *M*_2_(CH_2_–P)} that convey the relative proximities between the ^13^C atoms of the respective {COO^–^, CH, CH_2_} group and the inorganic phosphate moieties of ACP, as well as the *M*_2_(N–P) counterpart informing about the ACP contacts. The overall good agreement confirmed the accuracy of our metadynamics simulations, notably the validity of the herein employed HA-surface preparation protocol of ref (45), which produces a disordered apatite surface with pH-dependent phosphate speciation. For our experimental conditions of 3.8 ≤ pH ≤ 5.3 (Pser@HA and Pser16) and pH = 7.4 (Ser16), both Pser and Ser molecules anchor at their amino groups, together with the HPO_4_^−^ group of Pser and the COO^–^ group of Ser, which leads to an extended conformation of both surface-immobilized molecules. The OH group of Ser also participates in the binding at ACP *via* both electrostatic **O**H···**Ca**^2+^ interactions and H bonds to the inorganic phosphate groups, but they contribute overall little to the net adsorption energy (≈10%). The only discrepancy between the experiments and models concerned an underestimation of the metadynamics-derived COO^–^···ACP proximities relative to those deduced by NMR on the Pser@HA and Pser16 specimens.

Besides rationalizing the distinction in bone-adhesive properties of Pser and Ser doped α-Ca_3_(PO_4_)_2_-based cements (Section 3.7), our findings settle some earlier suggestions/speculations about which functional groups are involved in the molecular binding of Ser and Pser at nanocrystalline HA.^90,107−109^ We stress that the binding modes discussed herein (Table 1 and Figure 5) are the *most probable* ones from an energetic viewpoint over a distribution of several distinct but stable modes. The nature of the comparatively weak adsorption of small biomolecules (such as amino acids and oligopeptides) at structurally disordered CaP surfaces must be analyzed/discussed in terms of “distributions” and/or “effective contacts”, as those encoded by dipolar second moments. The existence of a distribution of very similar binding modes is indeed mirrored in the broad and typically asymmetric ^13^C/^15^N MAS NMR peak shapes observed from each functional group which, except for the COO^–^ moiety of Ser16, remained unresolved. The ^13^C–^31^P correlation NMR spectrum of the latter revealed four ^13^COO^–^ resonances from groups with different proximities to ACP: the two COO^–^ environments closest to the inorganic phosphates were tentatively attributed to those solely involved in electrostatic COO^–^···Ca^2+^ interactions (≈184 ppm) and H-bonded COO^–^··· moieties (≈170 ppm). However, most of the COO^–^ groups bind by both interaction types, as mirrored in a resonance-continuum across the 181–172 ppm spectral region.

We conclude by highlighting the power of the herein-implemented *combination* of advanced solid-state NMR experiments with metadynamics simulations for an enhanced probing of the detailed biomolecular binding at structurally disordered CaP surfaces, which remains essentially untapped but is potentially very rewarding. Another ubiquitous tool exploited herein concerns the recently introduced Debye–Hückel-based analysis^48^ reviewed in the Supporting Information, which offers a straightforward decomposition of the net modeled biomolecular binding energy into its contributions from the various functional groups of the surface-immobilized molecule, as well as for quantifying each individual electrostatic/H-bond interaction energy.

## Supporting Information Available

Experimental solid-state NMR conditions; metadynamics simulation parameters, data analysis, and further discussion; synthesis and characterization of Pser*; discussion on the ^1^H NMR shift of water in ACP; cement batch compositions; further data on the modeled surface binding and relative dipolar second moments; best-fit ^31^P NMR parameters; additional solid-state NMR spectra; and TEM images (PDF)

## References

[ref1] Weiner S.; Wagner H. D. THE MATERIAL BONE: Structural-Mechanical Function Relations. Annu. Rev. Mater. Sci. 1998, 28, 271–298. 10.1146/annurev.matsci.28.1.271.

[ref2] Rey C.; Combes C.; Drouet C.; Sfihi H.; Barroug A. Physico-Chemical Properties of Nanocrystalline Apatites: Implications for Biominerals and Biomaterials. Mater. Sci. Eng. C 2007, 27, 198–205. 10.1016/j.msec.2006.05.015.

[ref3] Combes C.; Cazalbou S.; Rey C. Apatite Biominerals. Minerals 2016, 6, 34 10.3390/min6020034.

[ref4] Edén M. Structure and Formation of Amorphous Calcium Phosphate and its Role as Surface Layer of Nanocrystalline Apatite: Implications for Bone Mineralization. Materialia 2021, 17, 101107 10.1016/j.mtla.2021.101107.

[ref5] Gower L. B. Biomimetic Model Systems for Investigating the Amorphous Precursor Pathway and Its Role in Biomineralization. Chem. Rev. 2008, 108, 4551–4627. 10.1021/cr800443h. 19006398 PMC3652400

[ref6] Addadi L.; Weiner S. Interactions between Acidic Proteins and Crystals: Stereochemical Requirements in Biomineralization. Proc. Natl. Acad. Sci. U.S.A. 1985, 82, 4110–4114. 10.1073/pnas.82.12.4110. 3858868 PMC397944

[ref7] Hunter G. K. Interfacial Aspects of Biomineralization. Curr. Opin. Solid State Mater. Sci. 1996, 1, 430–435. 10.1016/s1359-0286(96)80036-2.

[ref8] Sharma V.; Srinivasan A.; Nikolajeff F.; Kumar S. Biomineralization Process in Hard Tissues: The Interaction Complexity within Protein and Inorganic Counterparts. Acta Biomater. 2021, 120, 20–37. 10.1016/j.actbio.2020.04.049. 32413577

[ref9] Boskey A. L. Biomineralization: Conflicts, Challenges, and Opportunities. J. Cell. Biochem. 1998, 72, 83–91. 10.1002/(sici)1097-4644(1998)72:30/31+<83::aid-jcb12>3.0.co;2-f. 29345818

[ref10] Boskey A. L.; Villarreal-Ramirez E. Intrinsically Disordered Proteins and Biomineralization. Matrix Biol. 2016, 52–54, 43–59. 10.1016/j.matbio.2016.01.007. PMC4875856 26807759

[ref11] George A.; Veis A. Phosphorylated Proteins and Control over Apatite Nucleation, Crystal Growth, and Inhibition. Chem. Rev. 2008, 108, 4670–4693. 10.1021/cr0782729. 18831570 PMC2748976

[ref12] Isobe T.; Nakamura S.; Nemoto R.; Senna M.; Sfihi H. Solid-State Double Nuclear Magnetic Resonance Study of the Local Structure of Calcium Phosphate Nanoparticles Synthesized by a Wet-Mechanochemical Reaction. J. Phys. Chem. B 2002, 106, 5169–5176. 10.1021/jp0138936.

[ref13] Jäger C.; Welzel T.; Meyer-Zaika W.; Epple M. A Solid-State NMR Investigation of the Structure of Nanocrystalline Hydroxyapatite. Magn. Reson. Chem. 2006, 44, 573–580. 10.1002/mrc.1774. 16395729

[ref14] Wang Y.; Von Euw S.; Fernandes F. M.; Cassaignon S.; Selmane M.; Laurent G.; Pehau-Arnaudet G.; Coelho C.; Bonhomme-Coury L.; Giraud-Guille M.-M.; et al. Water-Mediated Structuring of Bone Apatite. Nat. Mater. 2013, 12, 1144–1153. 10.1038/nmat3787. 24193662

[ref15] Von Euw S.; Wang Y.; Laurent G.; Drouet C.; Babonneau F.; Nassif N.; Azaïs T. Bone Mineral: New Insights into its Chemical Composition. Sci. Rep. 2019, 9, 8456 10.1038/s41598-019-44620-6. 31186433 PMC6560110

[ref16] Yasar O. F.; Liao W.-C.; Mathew R.; Yu Y.; Stevensson B.; Liu Y.; Shen Z.; Edén M. The Carbonate and Sodium Environments in Precipitated and Biomimetic Calcium Hydroxy-Carbonate Apatite Contrasted with Bone Mineral: Insights from Solid-State NMR. J. Phys. Chem. C 2021, 125, 10572–10592. 10.1021/acs.jpcc.0c11389.

[ref17] Eichert D.; Sfihi H.; Combes C.; Rey C. Specific Characterisitics of Wet Nanocrystalline Apatites: Consequences on Biomaterials and Bone Tissue. Key Eng. Mater. 2004, 254–256, 927–930. 10.4028/www.scientific.net/kem.254-256.927.

[ref18] Huq N. L.; Cross K. J.; Reynolds E. C. Molecular Modelling of a Multiphosphorylated Sequence Motif Bound to Hydroxyapatite Surfaces. J. Mol. Model. 2000, 6, 35–47. 10.1007/s0089400060035.

[ref19] Hunter G. K.; O’Young J.; Grohe B.; Karttunen M.; Goldberg H. A. The Flexible Polyelectrolyte Hypothesis of Protein–Biomineral Interaction. Langmuir 2010, 26, 18639–18646. 10.1021/la100401r. 20527831

[ref20] Azzopardi P. V.; O’Young J.; Lajoie G.; Karttunen M.; Goldberg H. A.; Hunter G. K. Roles of Electrostatics and Conformation in Protein-Crystal Interactions. PLoS One 2010, 5, e9330 10.1371/journal.pone.0009330. 20174473 PMC2824833

[ref21] Addison W. N.; Miller S. J.; Ramaswamy J.; Mansouri A.; Kohn D. H.; McKee M. D. Phosphorylation-Dependent Mineral-Type Specificity for Apatite-Binding Peptide Sequences. Biomaterials 2010, 31, 9422–9430. 10.1016/j.biomaterials.2010.08.064. 20943264 PMC2976791

[ref22] Jaeger C.; Groom N. S.; Bowe E. A.; Horner A.; Davies M. E.; Murray R. C.; Duer M. J. Investigation of the Nature of the Protein–Mineral Interface in Bone by Solid-State NMR. Chem. Mater. 2005, 17, 3059–3061. 10.1021/cm050492k.

[ref23] Wise E. R.; Maltsev S.; Davies M. E.; Duer M. J.; Jaeger C.; Loveridge N.; Murray R. C.; Reid D. G. The Organic–Mineral Interface in Bone Is Predominantly Polysaccharide. Chem. Mater. 2007, 19, 5055–5057. 10.1021/cm702054c.

[ref24] Hu Y.-Y.; Rawal A.; Schmidt-Rohr K. Strongly Bound Citrate Stabilizes the Apatite Nanocrystals in Bone. Proc. Natl. Acad. Sci. U.S.A. 2010, 107, 22425–22429. 10.1073/pnas.1009219107. 21127269 PMC3012505

[ref25] Nikel O.; Laurencin D.; Bonhomme C.; Sroga G. E.; Besdo S.; Lorenz A.; Vashishth D. Solid State NMR Investigation of Intact Human Bone Quality: Balancing Issues and Insight into the Structure at the Organic–Mineral Interface. J. Phys. Chem. C 2012, 116, 6320–6331. 10.1021/jp2125312. PMC3399594 22822414

[ref26] Nikel O.; Laurencin D.; McCallum S. A.; Gundberg C. M.; Vashishth D. NMR Investigation of the Role of Osteocalcin and Osteopontin at the Organic–Inorganic Interface in Bone. Langmuir 2013, 29, 13873–13882. 10.1021/la403203w. 24128197 PMC3901427

[ref27] Tseng Y.-H.; Mou Y.; Chen P.-H.; Tsai T. W. T.; Hsieh C.-I.; Mou C.-Y.; Chan J. C. C. Solid-State P-31 NMR Study of the Formation of Hydroxyapatite in the Presence of Glutaric Acid. Magn. Reson. Chem. 2008, 46, 330–334. 10.1002/mrc.2096. 18306172

[ref28] Achelhi K.; Masse S.; Laurent G.; Saoiabi A.; Laghzizil A.; Coradin T. Role of Carboxylate Chelating Agents on the Chemical, Structural and Textural Properties of Hydroxyapatite. Dalton Trans. 2010, 39, 10644–10651. 10.1039/c0dt00251h. 20886132

[ref29] Hu Y.-Y.; Liu X. P.; Ma X.; Rawal A.; Prozorov T.; Akinc M.; Mallapragada S. K.; Schmidt-Rohr K. Biomimetic Self-Assembling Copolymer-Hydroxyapatite Nanocomposites with the Nanocrystal Size Controlled by Citrate. Chem. Mater. 2011, 23, 2481–2490. 10.1021/cm200355n.

[ref30] Wu Y.-J.; Tsai W. T.; Huang S.-J.; Mou Y.; Lin C.-J.; Chan J. C. C. Hydrogen Bond Formation between Citrate and Phosphate Ions in Spherulites of Fluorapatite. Langmuir 2013, 29, 11681–11686. 10.1021/la402392b. 24007413

[ref31] Davies E.; Müller K. H.; Wong W. C.; Pickard C. J.; Reid D. G.; Skepper J. N.; Duer M. J. Citrate Bridges between Mineral Platelets in Bone. Proc. Natl. Acad. Sci. U.S.A. 2014, 111, E1354–E1363. 10.1073/pnas.1315080111. 24706850 PMC3986129

[ref32] Wang Z.; Xu Z.; Zhao W.; Sahai N. A Potential Mechanism for Amino Acid-Controlled Crystal Growth of Hydroxyapatite. J. Mater. Chem. B 2015, 3, 9157–9167. 10.1039/c5tb01036e. 32263130

[ref33] Goobes G.; Stayton P. S.; Drobny G. P. Solid State NMR Studies of Molecular Recognition at Protein-Mineral Interfaces. Prog. Nucl. Magn. Reson. Spectrosc. 2007, 50, 71–85. 10.1016/j.pnmrs.2006.11.002. 19768124 PMC2746069

[ref34] Chen P.-H.; Tseng Y.-H.; Mou Y.; Tsai Y.-L.; Guo S.-M.; Huang S.-J.; Yu S. S.-F.; Chan J. C. C. Adsorption of a Statherin Peptide Fragment on the Surface of Nanocrystallites of Hydroxyapatite. J. Am. Chem. Soc. 2008, 130, 2862–2868. 10.1021/ja076607y. 18266360

[ref35] Iline-Vul T.; Nanda R.; Mateos B.; Hazan S.; Matlahov I.; Perelshtein I.; Keinan-Adamsky K.; Althoff-Ospelt G.; Konrat R.; Goobes G. Osteopontin Regulates Biomimetic Calcium Phosphate Crystallization from Disordered Mineral Layers Covering Apatite Crystallites. Sci. Rep. 2020, 10, 15722 10.1038/s41598-020-72786-x. 32973201 PMC7518277

[ref36] Matlahov I.; Iline-Vul T.; Abayev M.; Lee E. M. Y.; Nadav-Tsubery M.; Keinan-Adamsky K.; Gray J. J.; Goobes G. Interfacial Mineral-Peptide Properties of a Mineral Binding Peptide from Osteonectin and Bone-like Apatite. Chem. Mater. 2015, 27, 5562–5569. 10.1021/acs.chemmater.5b01696.

[ref37] Iline-Vul T.; Matlahov I.; Grinblat J.; Keinan-Adamsky K.; Goobes G. Changes to the Disordered Phase and Apatite Crystallite Morphology during Mineralization by an Acidic Mineral Binding Peptide from Osteonectin. Biomacromolecules 2015, 16, 2656–2663. 10.1021/acs.biomac.5b00465. 26207448

[ref38] Iline-Vul T.; Kulpanovich A.; Nadav-Tsubery M.; Semionov A.; Keinan-Adamsky K.; Goobes G. How Does Osteocalcin Lacking γ-Glutamic Groups Affect Biomimetic Apatite Formation and What Can we Say About its Structure in Mineral-Bound Form?. J. Struct. Biol. 2019, 207, 104–114. 10.1016/j.jsb.2019.04.014. 31015050

[ref39] Gullion T.; Schaefer J. Rotational-Echo Double-Resonance NMR. J. Magn. Reson. 1989, 81, 196–200. 10.1016/0022-2364(89)90280-1. 22152360

[ref40] de Leeuw N. H.; Rabone J. A. L. Molecular Dynamics Simulations of the Interaction of Citric Acid with the Hydroxyapatite (0001) and (011̅0) Surfaces in an Aqueous Environment. CrystEngComm 2007, 9, 1178–1186. 10.1039/b710974a.

[ref41] Jiang W.; Pan H.; Cai Y.; Tao J.; Liu P.; Xu X.; Tang R. Atomic Force Microscopy Reveals Hydroxyapatite–Citrate Interfacial Structure at the Atomic Level. Langmuir 2008, 24, 12446–12451. 10.1021/la801720w. 18823133

[ref42] Xu Z.; Yang Y.; Wang Z.; Mkhonto D.; Shang C.; Liu Z.-P.; Cui Q.; Sahai N. Small Molecule-Mediated Control of Hydroxyapatite Growth: Free Energy Calculations Benchmarked to Density Functional Theory. J. Comput. Chem. 2014, 35, 70–81. 10.1002/jcc.23474. 24272540

[ref43] Wang Z.; Xu Z.; Zhao W.; Chen W.; Miyoshi T.; Sahai N. Isoexergonic Conformations of Surface-Bound Citrate Regulated Bioinspired Apatite Nanocrystal Growth. ACS Appl. Mater. Interfaces 2016, 8, 28116–28123. 10.1021/acsami.6b04822. 27593160

[ref44] Heinz H.; Ramezani-Dakhel H. Simulations of Inorganic–Bioorganic Interfaces to Discover New Materials: Insights, Comparisons to Experiment, Challenges, and Opportunities. Chem. Soc. Rev. 2016, 45, 412–448. 10.1039/c5cs00890e. 26750724

[ref45] Lin T.-J.; Heinz H. Accurate Force Field Parameters and pH Resolved Surface Models for Hydroxyapatite to Understand Structure, Mechanics, Hydration, and Biological Interfaces. J. Phys. Chem. C 2016, 120, 4975–4992. 10.1021/acs.jpcc.5b12504.

[ref46] Lin T.-J. Predicting Binding Affinities of Nitrogen-Containing Bisphosphonates on Hydroxyapatite Surface by Molecular Dynamics. Chem. Phys. Lett. 2019, 716, 83–92. 10.1016/j.cplett.2018.12.008.

[ref47] Luo M.; Gao Y.; Yang S.; Quan X.; Sun D.; Liang K.; Li J.; Zhou J. Computer Simulations of the Adsorption of an N-Terminal Peptide of Statherin, SN15, and its Mutants on Hydroxyapatite Surfaces. Phys. Chem. Chem. Phys. 2019, 21, 9342–9351. 10.1039/c9cp01638d. 30994664

[ref48] Stevensson B.; Edén M. Metadynamics Simulations of the pH-Dependent Adsorption of Phosphoserine and Citrate on Disordered Apatite Surfaces: What Interactions Govern the Molecular Binding?. J. Phys. Chem. B 2021, 125, 11987–12003. 10.1021/acs.jpcc.1c02325.34672586

[ref49] Heinz H.; Lin T.-J.; Kishore Mishra R.; Emami F. S. Thermodynamically Consistent Force Fields for the Assembly of Inorganic, Organic, and Biological Nanostructures: The INTERFACE Force Field. Langmuir 2013, 29, 1754–1765. 10.1021/la3038846. 23276161

[ref50] Holt C.; Timmins P. A.; Errington N.; Leaver J. A Core-Shell Model of Calcium Phosphate Nanoclusters Stabilized by β-Casein Phosphopeptides, Derived from Sedimentation Equilibrium and Small-Angle X-Ray and Neutron-Scattering Measurements. Eur. J. Biochem. 1998, 252, 73–78. 10.1046/j.1432-1327.1998.2520073.x. 9523714

[ref51] Holt C.; Sørensen E. S.; Clegg R. A. Role of Calcium Phosphate Nanoclusters in the Control of Calcification. FEBS J. 2009, 276, 2308–2323. 10.1111/j.1742-4658.2009.06958.x. 19292864

[ref52] De Sa Peixoto P.; Silva J. V. C.; Laurent G.; Schmutz M.; Thomas D.; Bouchoux A.; Gésan-Guiziou G. How High Concentrations of Proteins Stabilize the Amorphous State of Calcium Orthophosphate: A Solid-State Nuclear Magnetic Resonance (NMR) Study of the Casein Case. Langmuir 2017, 33, 1256–1264. 10.1021/acs.langmuir.6b04235. 28094949

[ref53] Hindmarsh J. P.; Watkinson P. Experimental Evidence for Previously Unclassified Calcium Phosphate Structures in the Casein Micelle. J. Dairy Sci. 2017, 100, 6938–6948. 10.3168/jds.2017-12623. 28690066

[ref54] Pujari-Palmer M.; Guo H.; Wenner D.; Autefage H.; Spicer C. D.; Stevens M. M.; Omar O.; Thomsen P.; Edén M.; Insley G.; et al. A Novel Class of Injectable Bioceramics that Glue Tissues and Biomaterials. Materials 2018, 11, 2492 10.3390/ma11122492. PMC6316977 30544596

[ref55] Kirillova A.; Kelly C.; von Windheim N.; Gall K. Bioinspired Mineral–Organic Bioresorbable Bone Adhesive. Adv. Healthcare Mater. 2018, 7, 1800467 10.1002/adhm.201800467. 29938916

[ref56] Kesseli F. P.; Lauer C. S.; Baker I.; Mirica K. A.; Van Citters D. W. Identification of a Calcium Phosphoserine Coordination Network in an Adhesive Organo–Apatitic Bone Cement System. Acta Biomater. 2020, 105, 280–289. 10.1016/j.actbio.2020.01.007. 31945507 PMC7134197

[ref57] Liu X.; Pujari-Palmer M.; Wenner D.; Procter P.; Insley G.; Engqvist H. Adhesive Cements That Bond Soft Tissue Ex Vivo. Materials 2019, 12, 2473 10.3390/ma12152473. PMC6695630 31382566

[ref58] Pujari-Palmer M.; Giró R.; Procter P.; Bojan A.; Insley G.; Engqvist H. Factors that Determine the Adhesive Strength in a Bioinspired Bone Tissue Adhesive. Chem. Eng. 2020, 4, 19 10.3390/chemengineering4010019.

[ref59] Spicer C. D.; Pujari-Palmer M.; Autefage H.; Insley G.; Procter P.; Engqvist H.; Stevens M. M. Synthesis of Phospho-Amino Acid Analogues as Tissue Adhesive Cement Additives. ACS Cent. Sci. 2020, 6, 226–231. 10.1021/acscentsci.9b01149. 32123740 PMC7047273

[ref60] Mathew R.; Pujari-Palmer M.; Guo H.; Yu Y.; Stevensson B.; Engqvist H.; Edén M. Solid-State NMR Rationalizes the Bone-Adhesive Properties of Serine- and Phosphoserine-Bearing Calcium Phosphate Cements by Unveiling Their Organic/Inorganic Interface. J. Phys. Chem. C 2020, 124, 21512–21531. 10.1021/acs.jpcc.0c06224.

[ref61] Barducci A.; Bussi G.; Parrinello M. Well-Tempered Metadynamics: A Smoothly Converging and Tunable Free-Energy Method. Phys. Rev. Lett. 2008, 100, 020603 10.1103/PhysRevLett.100.020603. 18232845

[ref62] Valsson O.; Tiwary P.; Parrinello M. Enhancing Important Fluctuations: Rare Events and Metadynamics from a Conceptual Viewpoint. Annu. Rev. Phys. Chem. 2016, 67, 159–184. 10.1146/annurev-physchem-040215-112229. 26980304

[ref63] Brunauer S.; Emmett P. H.; Teller E. Adsorption of Gases in Multimolecular Layers. J. Am. Chem. Soc. 1938, 60, 309–319. 10.1021/ja01269a023.

[ref64] Suga T.; Okabe N. Aqua(L-O-Serine Phosphato)Calcium(II). Acta Crystallogr. 1996, 52, 1894–1896. 10.1107/s0108270196002648.

[ref65] Mathew R.; Stevensson B.; Edén M. Refined Structures of O-Phospho-L-serine and Its Calcium Salt by New Multinuclear Solid-State NMR Crystallography Methods. J. Phys. Chem. B 2021, 125, 10985–11004. 10.1021/acs.jpcb.1c05587. 34553936 PMC8503883

[ref66] Kay M. I.; Young R. A.; Posner A. S. Crystal Structure of Hydroxyapatite. Nature 1964, 204, 1050–1052. 10.1038/2041050a0. 14243377

[ref67] Abraham M. J.; Murtola T.; Schulz R.; Páll S.; Smith J. C.; Hess B.; Lindahl E. GROMACS: High Performance Molecular Simulations Through Multi-Level Parallelism from Laptops to Supercomputers. SoftwareX 2015, 1–2, 19–25. 10.1016/j.softx.2015.06.001.

[ref68] Bjelkmar P.; Larsson P.; Cuendet M. A.; Hess B.; Lindahl E. Implementation of the CHARMM Force Field in GROMACS: Analysis of Protein Stability Effects from Correction Maps, Virtual Interaction Sites, and Water Models. J. Chem. Theory Comput. 2010, 6, 459–466. 10.1021/ct900549r. 26617301

[ref69] Jorgensen W. L.; Chandrasekhar J.; Madura J. D.; Impey R. W.; Klein M. L. Comparison of Simple Potential Functions for Simulating Liquid Water. J. Chem. Phys. 1983, 79, 926–935. 10.1063/1.445869.

[ref70] Boonstra S.; Onck P. R.; van der Giessen E. CHARMM TIP3P Water Model Suppresses Peptide Folding by Solvating the Unfolded State. J. Phys. Chem. B 2016, 120, 3692–3698. 10.1021/acs.jpcb.6b01316. 27031562

[ref71] Laio A.; Gervasio F. L. Metadynamics: a Method to Simulate Rare Events and Reconstruct the Free Energy in Biophysics, Chemistry and Material Science. Rep. Prog. Phys. 2008, 71, 126601 10.1088/0034-4885/71/12/126601.

[ref72] Valsson O.; Parrinello M. Variational Approach to Enhanced Sampling and Free Energy Calculations. Phys. Rev. Lett. 2014, 113, 090601 10.1103/physrevlett.113.090601. 25215968

[ref73] Tribello G. A.; Bonomi M.; Branduardi D.; Camilloni C.; Bussi G. PLUMED 2: New Feathers for an Old Bird. Comput. Phys. Commun. 2014, 185, 604–613. 10.1016/j.cpc.2013.09.018.

[ref74] Ben Osman M.; Diallo-Garcia S.; Herledan V.; Brouri D.; Yoshioka T.; Kubo J.; Millot Y.; Costentin G. Discrimination of Surface and Bulk Structure of Crystalline Hydroxyapatite Nanoparticles by NMR. J. Phys. Chem. C 2015, 119, 23008–23020. 10.1021/acs.jpcc.5b08732.

[ref75] Turner G. L.; Smith K. A.; Kirkpatrick R. J.; Oldfieldt E. Structure and Cation Effects on Phosphorus-31 NMR Chemical Shifts and Chemical-Shift Anisotropies of Orthophosphates. J. Magn. Reson. 1986, 70, 408–415. 10.1016/0022-2364(86)90129-0.

[ref76] Hartmann P.; Vogel J.; Schnabel B. The Influence of Short-Range Geometry on the ^31^P Chemical-Shift Tensor in Protonated Phosphates. J. Magn. Reson., Ser. A 1994, 111, 110–114. 10.1006/jmra.1994.1234.

[ref77] Lu B.-Q.; Garcia N. A.; Chevrier D. M.; Zhang P.; Raiteri P.; Gale J. D.; Gebauer D. Short-Range Structure of Amorphous Calcium Hydrogen Phosphate. Cryst. Growth Des. 2019, 19, 3030–3038. 10.1021/acs.cgd.9b00274.

[ref78] Dorozhkin S. V. Calcium Orthophosphate Cements for Biomedical Application. J. Mater. Sci. 2008, 43, 3028–3057. 10.1007/s10853-008-2527-z.

[ref79] Reinstorf A.; Ruhnow M.; Gelinsky M.; Pompe W.; Hempel U.; Wenzel K.-W.; Simon P. Phosphoserine—a Convenient Compound for Modification of Calcium Phosphate Bone Cement Collagen Composites. J. Mater. Sci.: Mater. Med. 2004, 15, 451–455. 10.1023/b:jmsm.0000021119.14870.3d. 15332616

[ref80] Brunner T. J.; Grass R. N.; Bohner M.; Stark W. J. Effect of Particle Size, Crystal Phase and Crystallinity on the Reactivity of Tricalcium Phosphate Cements for Bone Reconstruction. J. Mater. Chem. 2007, 17, 4072–4078. 10.1039/b707171j.

[ref81] Aue W. P.; Roufosse A. H.; Glimcher M. J.; Griffin R. G. Solid-State Phosphorus-31 Nuclear Magnetic Resonance Studies of Synthetic Solid Phases of Calcium Phosphate: Potential Models of Bone Mineral. Biochemistry 1984, 23, 6110–6114. 10.1021/bi00320a032. 6525349

[ref82] Yu Y.; Guo H.; Pujari-Palmer M.; Stevensson B.; Grins J.; Engqvist H.; Edén M. Advanced Solid-State ^1^H/^31^P NMR Characterization of Pyrophosphate-Doped Calcium Phosphate Cements for Biomedical Applications: The Structural Role of Pyrophosphate. Ceram. Int. 2019, 45, 20642–20655. 10.1016/j.ceramint.2019.07.047.

[ref83] Rothwell W. P.; Waugh J. S.; Yesinowski J. P. High-Resolution Variable-Temperature ^31^P NMR of Solid Calcium Phosphates. J. Am. Chem. Soc. 1980, 102, 2637–2643. 10.1021/ja00528a020.

[ref84] Tropp J.; Blumenthal N. C.; Waugh J. S. Phosphorus NMR Study of Solid Amorphous Calcium Phosphate. J. Am. Chem. Soc. 1983, 105, 22–26. 10.1021/ja00339a006.

[ref85] Yesinowski J. P.; Eckert H. Hydrogen Environments in Calcium Phosphates: ^1^H MAS NMR at High Spinning Speeds. J. Am. Chem. Soc. 1987, 109, 6274–6282. 10.1021/ja00255a009.

[ref86] Mathew R.; Turdean-Ionescu C.; Yu Y.; Stevensson B.; Izquierdo-Barba I.; García A.; Arcos D.; Vallet-Regí M.; Edén M. Proton Environments in Biomimetic Calcium Phosphates Formed from Mesoporous Bioactive CaO–SiO_2_–P_2_O_5_ Glasses in Vitro: Insights from Solid-State NMR. J. Phys. Chem. C 2017, 121, 13223–13238. 10.1021/acs.jpcc.7b03469. PMC5484558 28663772

[ref87] Gan Z. ^13^C/^14^N Heteronuclear Multiple-Quantum Correlation with Rotary Resonance and REDOR Dipolar Recoupling. J. Magn. Reson. 2007, 184, 39–43. 10.1016/j.jmr.2006.09.016. 17029884

[ref88] Hu B.; Trébosc J.; Amoureux J. P. Comparison of Several Hetero-Nuclear Dipolar Recoupling NMR Methods to be Used in MAS HMQC/HSQC. J. Magn. Reson. 2008, 192, 112–122. 10.1016/j.jmr.2008.02.004. 18299242

[ref89] Yasar O. F.; Liao W.-C.; Stevensson B.; Edén M. Structural Role and Spatial Distribution of Carbonate Ions in Amorphous Calcium Phosphate. J. Phys. Chem. C 2021, 125, 4675–4693. 10.1021/acs.jpcc.0c10355.

[ref90] Moreno E. C.; Kresak M.; Hay D. I. Adsorption of Molecules of Biological Interest onto Hydroxyapatite. Calcif. Tissue Int. 1984, 36, 48–59. 10.1007/bf02405293. 6423236

[ref91] Jack K. S.; Vizcarra T. G.; Trau M. Characterization and Surface Properties of Amino-Acid-Modified Carbonate-Containing Hydroxyapatite Particles. Langmuir 2007, 23, 12233–12242. 10.1021/la701848c. 17963411

[ref92] Jahromi M. T.; Yao G.; Cerruti M. The Importance of Amino Acid Interactions in the Crystallization of Hydroxyapatite. J. R. Soc. Interface 2013, 10, 20120906 10.1098/rsif.2012.0906. 23269851 PMC3565740

[ref93] Tavafoghi M.; Cerruti M. The Role of Amino Acids in Hydroxyapatite Mineralization. J. R. Soc. Interface 2016, 13, 20160462 10.1098/rsif.2016.0462. 27707904 PMC5095212

[ref94] Rimola A.; Corno M.; Zicovich-Wilson C. M.; Ugliengo P. Ab Initio Modeling of Protein/Biomaterial Interactions: Glycine Adsorption at Hydroxyapatite Surfaces. J. Am. Chem. Soc. 2008, 130, 16181–16183. 10.1021/ja806520d. 18989958

[ref95] Almora-Barrios N.; Austen K. F.; de Leeuw N. H. Density Functional Theory Study of the Binding of Glycine, Proline, and Hydroxyproline to the Hydroxyapatite (0001) and (011̅0) Surfaces. Langmuir 2009, 25, 5018–5025. 10.1021/la803842g. 19397352

[ref96] Gerothanassis I. P. Oxygen-17 NMR Spectroscopy: Basic Principles and Applications (Part I). Prog. Nucl. Magn. Reson. Spectrosc. 2010, 56, 95–197. 10.1016/j.pnmrs.2009.09.002. 20633350

[ref97] Bryce D. L. Calcium Binding Environments Probed by ^43^Ca NMR Spectroscopy. Dalton Trans. 2010, 39, 8593–8602. 10.1039/c0dt00416b. 20574585

[ref98] Laurencin D.; Smith M. E. Development of ^43^Ca Solid State NMR Spectroscopy as a Probe of Local Structure in Inorganic and Molecular Materials. Prog. Nucl. Magn. Reson. Spectrosc. 2013, 68, 1–40. 10.1016/j.pnmrs.2012.05.001. 23398971

[ref99] Sundaralingam M.; Putkey F. F. Molecular Structures of Amino Acids and Peptides. II. A Redetermination of the Crystal Structure of L-O-Serine Phosphate. A Very Short Phosphate-Carboxyl Hydrogen Bond. Acta Crystallogr. 1970, 26, 790–800. 10.1107/s0567740870003138. 5536180

[ref100] Potrzebowski M. J.; Assfeld X.; Ganicz K.; Olejniczak S.; Cartier A.; Gardiennet C.; Tekely P. An Experimental and Theoretical Study of the ^13^C and ^31^P Chemical Shielding Tensors in Solid O-Phosphorylated Amino Acids. J. Am. Chem. Soc. 2003, 125, 4223–4232. 10.1021/ja029840z. 12670244

[ref101] Van Vleck J. H. The Dipolar Broadening of Magnetic Resonance Lines in Crystals. Phys. Rev. 1948, 74, 1168–1183. 10.1103/physrev.74.1168.

[ref102] Bertmer M.; Züchner L.; Chan J. C. C.; Eckert H. Short and Medium Range Order in Sodium Aluminoborate Glasses: 2. Site Connectivities and Cation Distributions Studied by Rotational Echo Double Resonance NMR Spectroscopy. J. Phys. Chem. B 2000, 104, 6541–6553. 10.1021/jp9941918.

[ref103] Strojek W.; Kalwei M.; Eckert H. Dipolar NMR Strategies for Multispin Systems Involving Quadrupolar Nuclei: ^31^P{^23^Na} Rotational Echo Double Resonance (REDOR) of Crystalline Sodium Phosphates and Phosphate Glasses. J. Phys. Chem. B 2004, 108, 7061–7073. 10.1021/jp037041c.

[ref104] Eckert H.; Elbers S.; Epping J. D.; Janssen M.; Kalwei M.; Strojek W.; Voigt U. Dipolar Solid State NMR Approaches Towards Medium-Range Structure in Oxide Glasses. Top. Curr. Chem. 2005, 246, 195–233. 10.1007/b98651. 22160291

[ref105] Stevensson B.; Mathew R.; Yu Y.; Edén M. Two Heteronuclear Dipolar Results at the Price of One: Quantifying Na/P Contacts in Phosphosilicate Glasses and Biomimetic Hydroxy-Apatite. J. Magn. Reson. 2015, 251, 52–56. 10.1016/j.jmr.2014.12.002. 25557863

[ref106] Edén M. Update on ^27^Al NMR Studies of Aluminosilicate Glasses. Annu. Rep. NMR Spectrosc. 2020, 101, 285–410. 10.1016/bs.arnmr.2020.07.002.

[ref107] Benaziz L.; Barroug A.; Legrouri A.; Rey C.; Lebugle A. Adsorption of O-Phospho-L-Serine and L-Serine Onto Poorly Crystalline Apatite. J. Colloid Interface Sci. 2001, 238, 48–53. 10.1006/jcis.2001.7450. 11350135

[ref108] Spanos N.; Klepetsanis P. G.; Koutsoukos P. G. Model Studies on the Interaction of Amino Acids With Biominerals: The Effect of L-Serine at the Hydroxyapatite–Water Interface. J. Colloid Interface Sci. 2001, 236, 260–265. 10.1006/jcis.2000.7396. 11401372

[ref109] Spanos N.; Koutsoukos P. G. Model Studies of the Effect of Orthophospho-L-Serine on Biological Mineralization. Langmuir 2001, 17, 866–872. 10.1021/la0010166.

