# Supplementary material for: Nuclear Magnetic Resonance and Metadynamics Simulations Reveal the Atomistic Binding of l-Serine and O-Phospho-l-Serine at Disordered Calcium Phosphate Surfaces of Biocements
Source: Chem Mater. 2022 Sep 26;34(19):8815–30. doi: 10.1021/acs.chemmater.2c02112 (PMC9558313; doi:10.1021/acs.chemmater.2c02112)
Supplement: Supplementary file 1 — cm2c02112_si_001.pdf [file cm2c02112_si_001.pdf]

Supporting Information for

Nuclear Magnetic Resonance and Metadynamics Simulations Reveal the Atomistic Binding of L-Serine and *O*-Phospho-L-Serine at Disordered Calcium Phosphate Surfaces of Biocements

Renny Mathew[a], Baltzar Stevensson[a], Michael Pujari-Palmer[b], Christopher S. Wood[c], Phillip R. A. Chivers[c], Christopher D. Spicer[c,d], Hélène Autefage[c], Molly M. Stevens[c,e], Håkan Engqvist[b], and Mattias Edén[a,*]

[a]Department of Materials and Environmental Chemistry, Stockholm University, SE-106 91 Stockholm, Sweden.
[b]Applied Material Science, Department of Engineering, Uppsala University, SE-751 21 Uppsala, Sweden.
[c]Department of Medical Biochemistry and Biophysics, Karolinska Institute, 171 77 Stockholm, Sweden.
[d]Department of Chemistry, University of York, Heslington, York, YO10 5DD, United Kingdom.
[e]Department of Materials, Department of Bioengineering, and Institute of Biomedical Engineering, Imperial College London, London SW7 2AZ, United Kingdom

[*]Corresponding author. E-mail: *mattias.eden@mmk.su.se*

Contents

**1.**	**Section S1.**	Solid-state NMR Experimentation
**2.**	**Section S2.**	Metadynamics Simulation Procedures and Data Analyses
**3.**	**Section S3.**	Synthesis and Characterization of U-[^{13}C, ^{15}N] Pser
**4.**	**Section S4.**	The ^{1}H NMR Signature of Structural Water in ACP
**5.**	**Table S1.**	Cement Batch Compositions
**6.**	**Table S2.**	Partial Charges Used in the Metadynamics Simulations and Debye–Hückel Analyses
**7.**	**Table S3.**	Adsorption Energies for the Pser and Ser Binding at HA
**8.**	**Table S4.**	Number of Bonds Between Pser/Ser and the HA Surface
**9.**	**Table S5.**	Ratios of Experimental and Calculated/Modeled Dipolar Second Moments
**10.**	**Table S6.**	Best-Fit ^{31}P NMR Parameters From Spectra Deconvolutions
**11.**	**Fig. S1.**	^{31}P and ^{13}C CPMAS NMR of Pser/Pser*
**12.**	**Fig. S2.**	TEM Images of Pser@HA Nanoparticles
**13.**	**Fig. S3.**	^{13}C CPMAS NMR Spectra from Pser@HA Preparations with Different [Pser]
**14.**	**Fig. S4.**	^{31}P MAS NMR Spectra From Cement Precursors
**15.**	**Fig. S5.**	^{13}C{^{31}P} D-HMQC Projections and ^{13}C CPMAS NMR Spectra
**16.**	**Fig. S6.**	^{13}C{^{31}P} REDOR NMR Spectra.
**17.**	**Fig. S7.**	Synthesis Scheme of Pser From Ser
**18.**	**Fig. S8.**	Solution ^{1}H NMR Spectra of Pser/Pser*
**19.**	**Fig. S9.**	Solution ^{13}C and ^{31}P NMR Spectra of Pser/Pser*
**20.**	**References**	

S1 Solid-State NMR Experimentation

S1.1 $^{1}H/^{31}P$ Single-Pulse, and $^{1}H \rightarrow ^{31}P$ and $^{1}H \rightarrow ^{13}C$ CP MAS NMR

All 1D NMR experiments described below were acquired at $B_0 = 9.4$ T. Single-pulse ^{1}H and ^{31}P NMR spectra were recorded at $\nu_r = 14.00$ kHz, using 90° rf pulses operating at the ^{1}H and ^{31}P nutation frequencies $\nu_{\mathrm{H}} \approx \nu_{\mathrm{P}} \approx 85$ kHz. The ^{1}H NMR acquisitions involved 16–32 accumulated NMR-signal transients and 10–60 s relaxation delays (τ_{relax}), whereas 16 transients were recorded for the ^{31}P counterparts, using $\tau_{\mathrm{relax}} = 1.0$ h before starting each single-pulse NMR experiment. These long delays ensured quantitative ^{31}P NMR-signal intensities from all co-existing phases of the cement, including α-TCP, whose ^{31}P T_1 relaxation is very slow. SPINAL-64 proton decoupling[S1] at the ^{1}H nutation frequency $\nu_{\mathrm{H}} = 90$ kHz (5.4 μs pulses) was applied during the signal detection of all single-pulse ^{31}P MAS and $^{1}H \rightarrow ^{31}P$ CPMAS experiments. ^{1}H NMR spectra from the Pser, Ser, and CaPser reference samples were recorded at $\nu_r = 34.00$ kHz using 90° pulses at $\nu_{\mathrm{H}} = 102$ kHz, $\tau_{\mathrm{relax}} = 5$ s, and 16 co-added transients.

The $^{1}H \rightarrow ^{31}P$ CPMAS NMR experimentation ($\nu_r = 14.00$ kHz) utilized a contact interval $\tau_{\mathrm{CP}} \approx 1.0$ ms and the modified Hartmann-Hahn condition $\nu_{\mathrm{H}} = \nu_{\mathrm{P}} + \nu_r$, where ν_{P} was ramped linearly[S2] by ± 4 kHz around $\nu_{\mathrm{P}} = 40$ kHz. The ^{1}H 90° rf pulse operated at $\nu_{\mathrm{H}} = 54$ kHz. The relaxation delays and the number of transients were 3–4 s and 512–1024, respectively.

$^{1}H \rightarrow ^{13}C$ CPMAS NMR experiments ($\nu_r = 9.00$ kHz) used $\tau_{\mathrm{CP}} \approx 1.5$ ms with $\nu_{\mathrm{H}} = \nu_{\mathrm{P}} + \nu_r$ and the ^{13}C rf amplitude ramped by ± 4 kHz around $\nu_{\mathrm{C}} = 40$ kHz. The ^{1}H 90° rf pulse operated at $\nu_{\mathrm{H}} = 54$ kHz. Between 512–2048 transients were recorded (depending on the Ser/Pser content of the sample), employing 2–4 s relaxation delays and SPINAL-64 decoupling at $\nu_{\mathrm{H}} = 80$ kHz during the ^{13}C NMR-signal detection.

S1.2 Heteronuclear $^{1}H\{^{31}P\}$ and $^{13}C\{^{31}P\}$ D-HMQC 2D NMR Experiments

All 2D NMR acquisitions implemented the States-TPPI procedure[S3] to accomplish absorptive 2D NMR peaks with frequency-sign discrimination along the indirect dimension. Each number of t_1 increments stated below refers to that of *each* real/imaginary data-set of the hypercomplex protocol. The lowest contour levels employed for the 2D NMR spectra presented herein range between 2–5% of the maximum NMR intensity. Resonance offsets were minimized by positioning each radio-frequency (rf) carrier frequency at the mid of the NMR-signal region throughout. The notation S{I} implies that the S-spins are detected directly, which for the 2D NMR spectra implies that the I (S) resonances appear along the vertical (horizontal) dimension, respectively

$^{1}H\{^{31}P\}$ D-HMQC[S4,S5] NMR spectra were acquired at $B_0 = 9.4$ T and $\nu_r = 34.00$ kHz (2.5 mm rotors) from the Ser16, Pser16, and Pser@HA specimens, using the protocol shown in Fig. 1a of ref. S5. HMQC was generated by the $SR4_1^2 \equiv \{R4_1^2R4_1^{-2}\}_0\{R4_1^2R4_1^{-2}\}_{120}\{R4_1^2R4_1^{-2}\}_{240}$ scheme,[S6] where $\{\cdots\}_\phi$ denotes an overall phase-shift by ϕ in degrees. One completed $SR4_1^2$ sequence was applied to the ^{1}H spins, giving an excitation period of $\tau_{\mathrm{exc}} = 6\tau_r = 176$ μs, where $\tau_r = \nu_r^{-1}$ is the rotor period. ^{1}H nutation frequencies of $\nu_{\mathrm{H}} = 2\nu_r = 68.0$ kHz[S6] and $\nu_{\mathrm{H}} = 111$ kHz were employed for ^{1}H–^{31}P dipolar recoupling and for the 90° pulses that interconvert anti-phase magnetization and HMQC,[S4,S5] respectively. All 90°/180° ^{31}P rf pulses operated at $\nu_{\mathrm{P}} = 100$ kHz. The dwell time during the HMQC evolution was rotor-synchronized: $\Delta t_1 = 4\tau_r$. Typically, (20–56)×(1024–2048) $t_1 \times t_2$ time-points were recorded, with $\Delta t_2 = 7.3$ μs, 1.5 s relaxation delays, and 256–1024 transients/t_1-value.

$^{13}C\{^{31}P\}$ D-HMQC NMR spectra were recorded from the Ser16 and Pser16 samples at $B_0 = 14.1$ T and $\nu_r = 24.00$ kHz (3.2 mm rotors). The same HMQC scheme as for the $^{1}H\{^{31}P\}$ experiments was employed, except for that the initial ^{13}C magnetization was prepared by $^{1}H \rightarrow ^{13}C$

CP with the following parameters: ν_H = 64 kHz; ν_C = 40 kHz ramped by ±2 kHz; τ_CP = 1.0 ms for Pser16 and τ_CP = 1.5 ms for Ser16. The HMQC excitation and reconversion periods were $\tau_\mathrm{exc} = 42\tau_r$ = 1.75 ms for Pser16 and $\tau_\mathrm{exc} = 48\tau_r$ =2.00 ms for Ser16. $10(t_1)\times1024(t_2)$ time-points were recorded, with $\Delta t_1 = 4\tau_r$, Δt_2 = 7.3 μs, and 1.5 s relaxation delays, along with 8192 (Pser16) and 10240 (Ser16) transients/t_1-value, altogether giving the respective durations of 68 h and 85 h for the entire 2D NMR (States) acquisitions. SPINAL-64 decoupling at ν_H = 105 kHz (4.6 μs pulses) was applied during both t_1 and t_2 evolutions periods.

S1.3 Heteronuclear ^{15}N{^{31}P} and ^{13}C{^{31}P} REDOR NMR Experiments

S1.3.1 Background and Experimental conditions

^{13}C{^{31}P} and ^{15}N{^{31}P} REDOR NMR experiments were utilized to probe the spatial proximities between the S={^{13}C, ^{15}N} sites of the surface-bound Pser/Ser molecules and neighboring ^{31}P sites of phosphate groups at the HA/ACP surface. A heteronuclear S_j–$^{31}\mathrm{P}_k$ pair with a (short) internuclear distance distance $r^{jk}_\mathrm{S–P}$ interacts *via* the through-space *dipolar interaction*, whose strength is given by the *dipolar coupling constant* $b^{jk}_\mathrm{S–P}$,

$$b^{jk}_\mathrm{S–P} = \frac{-\mu_0}{8\pi^2}\hbar\gamma_\mathrm{S}\gamma_\mathrm{P}\left(r^{jk}_\mathrm{S–P}\right)^{-3} \quad [\text{unit of s}^{-1}\text{=Hz}], \tag{S1}$$

where γ_S and γ_P are the magnetogyric ratios of S and ^{31}P, respectively. Note that a *short* S_j–P_k interatomic distance is associated with a *large* dipolar coupling constant. The S{I} notation in the REDOR[S7] context implies detection of spin species S={^{13}C, ^{15}N}, whereas a train of rotor-synchronized 180° pulses are applied to spins I≡^{31}P to *recouple* ("restore") the otherwise MAS-averaged S–I interactions during a *recoupling* (or *dephasing*) period $\tau_\mathrm{rec} = 2n\tau_r$, with n = 1, 2, 3, ... The recoupled S–^{31}P dipolar interactions lead to a "*dephased*" (diminished) integrated S-spin NMR signal intensity, $S(\tau_\mathrm{rec})$, as compared to a REDOR "reference" experiment $S_0(\tau_\mathrm{rec})$, which constitutes a Hahn echo of total duration $2n\tau_r$ without recoupling pulses, but otherwise recorded under identical experimental conditions.[S7,S8] An example is given in Fig. **S6**.

^{13}C{^{31}P} and ^{15}N{^{31}P} REDOR NMR data were collected from the Ser16, Pser16, and Pser@HA samples for increasing dephasing periods at B_0 = 14.1 T and MAS rates of ν_r = 14.00 kHz for the ^{13}C{^{31}P} experiments and ν_r = 10.00 kHz for the ^{15}N{^{31}P} counterparts. Moreover, ^{15}N{^{31}P} REDOR NMR experiments were performed on the Pser* specimen, as well as ^{13}C{^{31}P} REDOR NMR on the polycrystalline powders of Pser (Flamma SpA) and CaPser.[S9] The ^{31}P 180° pulses for recoupling the heteronuclear ^{13}C–^{31}P and ^{15}N–^{31}P interactions were of durations 6.2 μs and 8.5 μs, respectively, and were cycled according to the XY8 scheme to minimize rf-pulse errors.[S10] The corresponding Hahn-echo 180° pulses were 7.8 μs (^{13}C) and 20 μs (^{15}N). The dephasing experiments started from ^{13}C or ^{15}N magnetization prepared by CP from protons, using contact periods of 1.5 ms for the experiments on Ser16 and Pser@HA, and 0.75 ms for Pser, CaPser and Pser16. Other cross-polarization conditions were as follows: ν_H = 60 kHz and ν_C = 46 kHz with ±2.3 kHz ramp for ^{1}H→^{13}C, and ν_H = 40 kHz and ν_N = 30 kHz with ±0.3 kHz ramp for ^{1}H→^{15}N. For each sample and τ_rec value, 3 independent NMR-data blocks were acquired, using τ_relax = 2 s and 512–2048 accumulated signal transients/block. All REDOR experiments employed SPINAL-64 ^{1}H decoupling at ν_H = 95 kHz during the dephasing and NMR-signal acquisition periods.

S1.3.2 Dipolar Second Moment Analysis

The normalized integrated S={^{13}C, ^{15}N} REDOR NMR-signal intensity,

$$\Delta S/S_0 \equiv [S_0(\tau_{\rm rec}) - S(\tau_{\rm rec})]/S_0(\tau_{\rm rec}),$$

is for "short" recoupling intervals, $M_2(\mathrm{S}_j\text{–}^{31}\mathrm{P})\tau_{\rm rec}^2 \ll 1$, proportional to the van Vleck *heteronuclear dipolar second moment*,[S11] M_2(S–P) (in units of s^{-2}=Hz^2), according to[S12–S16]

$$\Delta S/S_0 = \frac{16}{3} M_2(\mathrm{S}_j\text{–P})\tau_{\rm rec}^2, \text{ with } \mathrm{S}_j=\{^{15}\mathbf{N}\mathrm{H}_3^+ \ ^{13}\mathbf{C}\mathrm{OO}^-, \ ^{13}\mathbf{C}\mathrm{H}, \ ^{13}\mathbf{C}\mathrm{H}_2\}. \quad \text{(S2)}$$

Consequently, the initial NMR signal-dephasing depends parabolically on $\tau_{\rm rec}$, whereas $\Delta S/S_0$ approaches unity for large $\tau_{\rm rec}$-values because $S(\tau_{\rm rec}) \approx 0$, as we verified for each sample and ^{15}N{^{31}P} and ^{13}C{^{31}P} REDOR NMR experiment.

The $\{\tau_{\rm rec}, \Delta S/S_0\}$ data associated with each functional group was obtained by integrating the NMR peak areas, which for the cements often required spectra deconvolution by using the DMfit software[S17] to obtain each S and S_0 value of the partially overlapping aliphatic ^{13}C resonances. This also concerned the $^{13}\mathbf{C}\mathrm{OO}^-$ NMR-signal region of the Ser16 sample, which comprised several overlapping resonances (see Fig. **6**h and **7**c), whose complex NMR peakshape was deconvoluted into four ^{13}C NMR peaks at the chemical shifts $\delta_{\rm C} \approx$\{184, 177, 174.5, 166\} ppm. Their associated dipolar second moments were subsequently extracted from eq. S2, except for a minor NMR peak at $\delta_{\rm C} \approx$166 ppm, whose origin is unknown; it was omitted in the detailed analyses because these ^{13}C sites are sufficiently distant from ^{31}P sites at HA/ACP to yield an essentially negligible ^{13}C{^{31}P} REDOR NMR dephasing, as also concluded by the absence of this resonance in the ^{13}C{^{31}P} D-HMQC 2D NMR spectrum (Fig. **7**c).

Fitting the $\{\tau_{\rm rec}, \Delta S/S_0\}$ data set with $\Delta S/S_0 \lesssim 0.15$ to eq. S2 for each of the three independent ^{15}N{^{31}P} and ^{13}C{^{31}P} REDOR NMR experiments of each specimen, resulted in sets of estimated M_2(N–P) and \{M_2(CO–P), M_2(CH–P), M_2($\mathrm{CH_2}$–P)\} values for all ^{15}N/$^{13}\mathrm{C}_j$ sites. These second-moment estimates were used to calculate the average M_2(N–P) and \{$M_2(\mathrm{C}_j$–P)\} values and their accompanying $\pm1\sigma$ uncertainties listed in Table **2**. Note that all dipolar second moments are expressed in units of $\mathrm{kHz}^2 \equiv 1000\mathrm{s}^{-2}$, meaning that each M_2 value is a factor of $4\pi^2$ smaller than if it would be expressed in units of $\mathrm{rad}^2\mathrm{s}^{-2}$ (as employed, for instance in refs. S11–S14.)

The REDOR NMR-derived dipolar second moments may be contrasted with those calculated from the atom coordinates of a given relevant structure model,

$$M_2(\mathrm{S}_j\text{–}^{31}\mathrm{P}) = \frac{1}{5}\sum_{k=1}^{N_{\rm P}}(b_{\rm S\text{–}P}^{jk})^2 = \frac{\mu_0^2\hbar^2\gamma_{\rm S}^2\gamma_{\rm P}^2}{320\pi^4}\sum_{k}^{N_{\rm P}}(r_{\rm S\text{–}P}^{jk})^{-6}, \text{ with } \mathrm{S}_j=\{^{15}\mathbf{N}\mathrm{H}_3^+, \ ^{13}\mathbf{C}\mathrm{OO}^-, \ ^{13}\mathbf{C}\mathrm{H}, \ ^{13}\mathbf{C}\mathrm{H}_2\}, \quad \text{(S3)}$$

where $N_{\rm P}$ is the number of nearby $^{31}\mathrm{P}_k$ sites over a radius of 4.0 nm. Note that the overall slower dephasing dynamics under the ^{15}N–^{31}P dipolar interactions (Fig. **9**c) than those of ^{13}C–^{31}P (Fig. **8**) reflect the lower magnetogyric ratio of ^{15}N: $\gamma_{\rm N}/\gamma_{\rm C} = 0.40$. This implies that for equal ^{13}C–^{31}P and ^{15}N–^{31}P distances, the respective ratios of the dipolar coupling constants (eq. S1) and second moments (eq. S3) relate as $b_{\rm N\text{–}P}^{jk}/b_{\rm C\text{–}P}^{jk} = 0.40$ and $M_2(\mathrm{N\text{–}P})/M_2(\mathrm{C\text{–}P}) = 0.40^2 = 0.16$, respectively.

S1.3.3 Discussion on the REDOR-Derived Dipolar Second Moments

As commented in section **3.5**, the experimental M_2(N–P) and $M_2(\mathrm{C}_j$–P) data from the Ser16, Pser16, and Pser@HA specimens were used for validating the metadynamics-generated models, whereas the accuracy of our experimental \{$M_2(^{13}\mathrm{C}_j\text{–}^{31}\mathrm{P})$\} estimates were confirmed by the good agreement observed between the NMR-derived dipolar second moments obtained from the Pser/CaPser powders and those calculated from the crystal structures of Pser/CaPser.

Yet, dipolar second moments obtained from (in particular) structurally disordered systems by dipolar dephasing NMR techniques are in general lower than the counterparts calculated from diffraction-derived crystal structures.[S13–S16] The consistently lower M_2 estimates by NMR is attributed primarily to inhomogeneities in the rf field across the sample. However, to reduce these effects, we adopted the common approach to restrict the sample length between two Teflon "spacers" to the center 1/3 volume of the rotor. Given these precautions, the degree of underestimation of the $M_2(\mathrm{C}_j\text{–P})$ values of the Pser and CaPser samples (≈24%; Table **S5**) is larger than expected, for unknown reasons. Nonetheless, the *well-confined* $M_2^{\mathrm{NMR}}/M_2^{\mathrm{calc}}$ ratios at 0.76±0.01 for all dipolar second moments of the Pser and CaPser reference samples (except for $M_2(\mathrm{CO–P}) = 0.73$ of CaPser) is noteworthy. In principle, all NMR-derived dipolar second moments of Table **2** could be corrected by scaling each value by $0.76^{-1} = 1.32$, as is frequently utilized in the literature.[S13–S16] Yet, herein we avoided such corrections and Table **2** lists all "as-obtained" experimental/calculated dipolar second moments. Consequently, for a "perfect" metadynamics prediction, the value M_2^{model} calculated from its structure model should be 1.32 times that of M_2^{NMR}.

We stress that the same rotor was utilized for both $^{13}\mathrm{C}\{^{31}\mathrm{P}\}$ and $^{15}\mathrm{N}\{^{31}\mathrm{P}\}$ REDOR NMR experiments for each of the respective Ser16, Pser16, and Pser@HA samples, meaning that the impact from rf inhomogeneity should be identical in both experiments and thereby lead to the same correction factor $M_2^{\mathrm{NMR}}/M_2^{\mathrm{calc}}$ for both $\{M_2(\mathrm{C}_j\text{–P})\}$ and $M_2(\mathrm{N–P})$ values. Although there may still be minor variations *among* the samples due to different powder-packing efficiencies and minute differences in the precise sample confinements, the near-constant $M_2^{\mathrm{NMR}}/M_2^{\mathrm{calc}}$ ratios (Table **S5**) suggest them to be largely immaterial. The expected minor extent of these effects may be gauged by contrasting the $M_2^{\mathrm{NMR}}/M_2^{\mathrm{calc}} \approx 0.76$ result obtained from the $^{13}\mathrm{C}\{^{31}\mathrm{P}\}$ REDOR NMR experiments on the "Pser" powder with the *distinct* rotor/powder of the isotopically enriched Pser* specimen used for the $^{15}\mathrm{N}\{^{31}\mathrm{P}\}$ REDOR NMR experiments, which yielded $M_2^{\mathrm{NMR}}/M_2^{\mathrm{calc}} \approx 0.71$. Hence, the very low and essentially equal $M_2^{\mathrm{NMR}}(\mathrm{N–P})$ values from the two cements relative to the Pser@HA sample (section **3.6**) are very surprising and difficult to reconcile with another scenario than that the amino-group binding is weaker in the two cements than in the Pser@HA sample.

S2 Metadynamics Simulation Procedures and Data Analyses

S2.1 Simulated System

"HA slabs" with accompanying structurally disordered and pH-dependent surfaces were generated by starting from the monoclinic ($P2_1/b$) HA crystal modification,[S18] from which a $4 \times 2 \times 6$ supercell was obtained with equal lengths of 3.768 nm along both directions of the a=0.942 nm and b=1.884 nm unit-cell axes.[S18] The center of the crystal was subsequently cut into two pieces (see Fig. **1**a), whereupon the Ca^{2+}, and PO_4^{3-} ions were distributed equally among the two resulting surfaces, whereas all hydroxy surface groups were removed. The surface and lattice of the slab, representative for either the (100) or (001) hexagonal $P6_3/m$ HA types in contact with an aqueous solution of pH=4.5 or pH=7.4, was prepared according to the protocol of ref. S19: Randomly selected PO_4^{3-} sites were protonated to yield a net phosphate speciation dictated by the pH value of the solution and the $H_2PO_4^-/HPO_4^{2-}$ $\mathrm{p}K_\mathrm{a}$ values (Fig. **1**), accompanied by removal of Ca^{2+} ions at the surface to preserve charge balance. The resulting HA slab was subsequently converted into the $P6_3/m$ form[S20] by reversing the OH orientations throughout randomly selected c channels of the HA lattice.

The three-component system of the HA slab and a water phase with one Pser or Ser molecule was generated using the PACKMOL v17 program.[S21] The slab of bulk HA and its (100)/(001) surface was initially placed in a box of dimensions $\{l_x,\ l_y,\ l_z\} \approx \{3.77,\ 4.13,\ 7.78\}$ nm and $\{l_x,\ l_y,$

$l_z\} \approx \{3.77, 3.26, 10.16\}$ nm for the simulations involving (100) and (001), respectively; see Fig. **1**a. The box was then filled randomly with 2600 H_2O molecules along with one Ser or Pser molecule, for the latter together with one (pH=4.5) or two (pH=7.4) Na^+ cations for balancing the organic HPO_4^- and PO_4^{2-} moieties, respectively. The precise box dimensions were subsequently adjusted slightly by energy-optimization *via* MD simulations, as described in ref. S22.

The pH value in the Ser-based simulations match well the experimentally measured value of pH=7.1 for the Ser16 cement paste, whereas the value pH=4.5 accounts for both the experimentally relevant conditions of pH=5.3 and pH=3.85 for Pser@HA and Pser16, respectively; see Table **S1**. Notably, because Fig. **1** reveals that the phosphate speciation at the HA surface remain essentially invariant across the range $3.2 \leqslant \mathrm{pH} \leqslant 6.2$—for which $H_2PO_4^{2-}$ species accounts for >90% of all phosphate moieties of the outermost surface layer—the expected minor overestimation of the pH value of Pser16 (Table **S1**) becomes immaterial for the modeled results. Also note that for practical reasons, the simulations of Pser assumed *either* the HPO_4^- (pH=4.5) or the PO_4^{2-} (pH=7.4) protonation state of the organic phosphate moiety.[S22] This accounts accurately for the expected phosphate protonation in the Pser16 sample, whereas from the higher pH=5.3 of the aqueous solution surrounding the Pser@HA particles, we predict fractional populations 0.75:0.25 of the organic HPO_4^-:PO_4^{2-} moieties (Fig. **1**).

S2.2 Molecular Dynamics Simulations

The atomistic MD simulations involved NVT ensembles at T=37 °C, utilizing the GROMACS v2018.1 platform[S23] and the force fields specified in section **2.3**. The partial charges of the atoms are given in Table **S2**. The equations of motion were integrated by using the velocity Verlet integrator[S24] with a short time step of 0.9 fs to accurately account for the H-atom vibrations. Coulomb interactions were calculated with a smoothed particle-mesh Ewald summation[S25] of order 4 and a tolerance of 10^{-5}, using a 0.12 Fourier spacing and a 1.2 nm switch distance. All van der Waals interactions were truncated at 1.2 nm. The temperature was controlled by the velocity rescale thermostat[S26] with a 1 ps time constant.

S2.3 Metadynamics Simulations and Structure Analysis

The metadynamics computations[S27,S28] employed the VES protocol[S29] implemented in PLUMED2.4,[S30] using a well-tempered target distribution with a bias factor $\gamma = 5$, and 32 independent "walkers". The bias potential,

$$V(\mathbf{s}) = \sum_{j=0}^{128} \sum_{k=-128}^{128} \alpha_{jk} \cos\{2\pi j s_1/128\} \exp\{-\mathrm{i}2\pi k s_2/256\}, \tag{S4}$$

was parameterized by two collective variables, $\mathbf{s} = \{s_1,\ s_2\}$, where s_1 represents the distance between the center of the HA slab and the $\mathbf{C}OO^-$ and $\mathbf{P}O_4^-$ atom of the respective Ser and Pser molecule, while $s_2 = \tanh[(\log\{-E_{\mathrm{DH}}(\mathrm{O{-}Ca})\}+\alpha)/\alpha]$[S31,S32] depends on the electrostatic-interaction energy between the negatively charged O atoms of the phosphate (carboxy) group of Pser (Ser) and Ca^{2+} cations at the HA surface,[S22] where α=20 and E_{DH}(O–Ca) is defined by eq. S11. See ref. S22 for further details. The α_{jk} coefficients and the well-tempered target distribution $P(\mathbf{s})^{1/\gamma}$ (where $P(\mathbf{s})$ is the unbiased probability distribution) were calculated iteratively during the simulation by using the averaged stochastic gradient descent algorithm[S33] with a step size of 0.001. The $\{\alpha_{jk},\ P(\mathbf{s})\}$ values were updated every 0.9 ps.

The average value and its uncertainty of each structure-related observable (O) was calculated

by using the last 30 ns of each simulated trajectory of the 32 walkers,[S28,S34] each sampled every 90 ps and calculated out to $\gtrsim$40 ns. The *Helmholtz free energy* of the system was obtained from[S29]

$$F(\mathbf{s}) = -V(\mathbf{s}) - k_\mathrm{B}T \log\left\{P(\mathbf{s})^{1/\gamma}\right\} - k_\mathrm{B}T \log\left\{\int \mathrm{d}\mathbf{s} \exp\{-[F(\mathbf{s}) + V(\mathbf{s})]/(k_\mathrm{B}T)\}\right\} \tag{S5}$$

whereas all other simulated observables were recovered by "reweighting",[S28,S29,S35,S36]

$$\langle O(\mathbf{R})\rangle = \frac{\int \mathrm{d}t O(\mathbf{R}) \exp\{[V(\mathbf{s}(\mathbf{R}),t) - c(t)]/(k_\mathrm{B}T)\}}{\int \mathrm{d}t \exp\{[V(\mathbf{s}(\mathbf{R}),t) - c(t)]/(k_\mathrm{B}T)\}}, \tag{S6}$$

where $\mathbf{R}$ represents spatial coordinates, k_B is Boltzmann's constant, and the bias correction function, $c(t)$, was estimated by[S36]

$$c(t) = k_\mathrm{B}T \log\left\{\frac{\int \mathrm{d}\mathbf{s} \exp\{V(\mathbf{s},t)\gamma/[(\gamma-1)k_\mathrm{B}T]\}}{\int \mathrm{d}\mathbf{s} \exp\{V(\mathbf{s},t)/[(\gamma-1)k_\mathrm{B}T]\}}\right\}. \tag{S7}$$

To asses the adsorption strength of a molecule $S = \{\mathrm{Ser, Pser}\}$, we employed the difference in the Helmholtz free energies F_B and F_F between the *bound* (B) and *free* (F) forms of S, respectively, referred to as the Helmholtz free energy of adsorption, and given by

$$\Delta F_\mathrm{ads} = F_\mathrm{B} - F_\mathrm{F} = k_\mathrm{B}T \log\left\{\frac{P_\mathrm{B}}{P_\mathrm{F}}\frac{[S]}{[S]^\ominus}\right\}. \tag{S8}$$

Here $[S]$ is the concentration (mol/L) of the substrate in the solution (i.e., the "free state") employed in the simulation, $[S]^\ominus = 1$ mol/L is the standard concentration, and P_B and P_F are the probabilities of the respective bound and free states, which were calculated by

$$P_\mathcal{S} = \int_\mathcal{S} d\mathbf{s} \exp\{-F(\mathbf{s})/(k_\mathrm{B}T)\}/\int_\mathcal{S} d\mathbf{s}, \text{ with } \mathcal{S} = \{\mathrm{B,\ F}\}. \tag{S9}$$

ΔF_ads is related to the *internal* energy of adsorption, ΔU_ads, by the term $E^\ominus = -8.15$ kJ/mol:

$$\Delta U_\mathrm{ads} = \Delta F_\mathrm{ads} - E^\ominus. \tag{S10}$$

Note that the more negative the value of ΔF_ads and ΔU_ads, the stronger the surface-binding (adsorption).

S2.4 Debye-Hückel Analysis

Stevensson and Edén introduced a straightforward analysis protocol[S22] based on Debye-Hückel (DH) energies[S37,S38] for identifying the primary bonding types ("contact modes") and assessing the relative interaction-energy contributions among the functional groups $\{COO^-, NH_3^+, \ldots\}$ towards the molecular binding at an inorganic surface.

The Debye-Hückel pair-interaction energy, $E_\mathrm{DH}(A\text{–}B)$, is given by[S31,S37,S38]

$$E_\mathrm{DH}(A\text{–}B) = \kappa q_A q_B \sum_j \sum_k \left(r_{AB}^{jk}\right)^{-1}, \tag{S11}$$

and involves one positively/negatively charged species "A" of the adsorbed molecule and an oppositely-charged cation/anion species "B" of the HA surface, i.e., an atom/ion out of the $\{Ca^{2+}$, $H_2PO_4^{2-}$, HPO_4^{2-}, $PO_4^{3-}\}$ groups. Here r_{AB}^{jk} denotes the distance between two sites j and k of

atom/ion species A and B, respectively, which carry the partial charges q_A and q_B given in Table **S2**. The constant $\kappa = e^2 N_\mathrm{A}/(4\pi\epsilon_0\epsilon_\mathrm{r})$ depends on the elementary charge (e), Avogadro's constant (N_A), the relative permeability ($\epsilon_\mathrm{r} = 80$), and the permeability of free space (ϵ_0).

The sum in eq. S11 includes all distances within a cutoff radius ($r_{AB}^{jk} \leqslant r_\mathrm{c}$), taken as $r_\mathrm{c} = 360$ pm for all electrostatic (ion–ion) interactions and $r_\mathrm{c} = 225$ pm for all H bonds.[S22] For example, the net DH energy of E_DH(C**O**–**Ca**) is calculated by summing all O–Ca pairs stemming from the two O atoms of the carboxy group and all nearby Ca^{2+} ions, whereas the H-bond interaction energy E_DH(N**H**–P**O**$_4$) is the sum over all bonds encountered between the protons of the amino group and negatively charged O neighbors of inorganic phosphate moieties; see ref. S22 for details. Table **S3** lists the various Debye-Hückel energy contributions from each functional group of the Pser and Ser molecules when bound at the (100) or (001) HA surfaces for pH={4.5, 7.4}. The *coordination number*, $Z(A$–$B)$, represents the number of atoms B around A (i.e., the number of A–B pairs) and is defined according to[S22]

$$Z(A\text{–}B) = \sum_j \sum_k \left\{ \begin{array}{ll} 1 & \text{if } r_{AB}^{jk} \leqslant r_\mathrm{c} \\ 0 & \text{if } r_{AB}^{jk} > r_\mathrm{c} \end{array} \right. . \tag{S12}$$

The $Z(A$–$B)$ data obtained by applying eq. S12 to the metadynamics-derived data of the Pser/Ser adsorption are collected in Table **S4**.

The net Debye-Hückel energies associated with the binding of a molecule at a given surface is denoted $E_\mathrm{DH}^\mathrm{tot}$ and involves a summation over all pair-interactions of all functional groups of the molecule and their bonding partners B at the HA surface:

$$E_\mathrm{DH}^\mathrm{tot} = \sum_{A,B} E_\mathrm{DH}\,(A\text{–}B)\,. \tag{S13}$$

Notably, $E_\mathrm{DH}^\mathrm{tot}$ may be contrasted directly with the metadynamics-derived entity ΔU_ads obtained from eq. S8. As demonstrated in ref. S22, the Debye-Hückel energies approximates very well the modeled ΔU_ads data within a typical error below $\approx 10\%$, as may be verified from the ΔU_ads and $E_\mathrm{DH}^\mathrm{tot}$ results of Table **S3**. The feature $E_\mathrm{DH}^\mathrm{tot} \approx \Delta U_\mathrm{ads}$ ensures that the outcomes from the DH analysis is representative also for the modelled thermodynamic entities.

The relative contribution $[f(G)]$ from each functional group G of a surface-immobilized molecule to the net adsorption energy is straightforward to estimate from the expression

$$f(G) = E_\mathrm{DH}(G)/E_\mathrm{DH}^\mathrm{tot}, \tag{S14}$$

where $E_\mathrm{DH}(G)$ is readily obtained by summing all relevant $E_\mathrm{DH}(A$–$B)$ terms calculated from eq. S11. Given that $E_\mathrm{DH}(G) \approx 0$ for all functional groups without direct bonds to the HA surface (such as the aliphatic groups of Pser/Ser), the relationships $E_\mathrm{DH}(PO_4) + E_\mathrm{DH}(COO) + E_\mathrm{DH}(NH_3) = E_\mathrm{DH}^\mathrm{tot}$ and $E_\mathrm{DH}(COO) + E_\mathrm{DH}(NH_3) + E_\mathrm{DH}(OH) = E_\mathrm{DH}^\mathrm{tot}$ hold for Pser and Ser, respectively. The sets of $\{f(G)\}$ data for Ser and Pser are listed in Table **1**.

S2.5 Generation of Realistic HA/H_2O Interfaces For Modeling of Biomolecular Adsorption

An outstanding problem in computational modeling of biomolecular adsorption at nanocrystalline HA particles concerns how structural disorder is accounted for and how pH effects on the chemical speciation at the HA surface are emulated. First and foremost, the precise Ca^{2+}/phosphate speciations at a nanocrystalline HA-particle surface remains largely unknown, notably so its de-

tailed (distributions of) $H_nPO_4^{(3-n)-}$ protonation states. It is known from Fourier-Transform Infrared (FTIR) and solid-state NMR spectroscopy results that the surface of both bone-mineral and nanocrystalline HA particles generated *in vitro* under neutral or weakly alkaline conditions is dominated by protonated phosphate groups that are often attributed entirely to HPO_4^{2-} moieties.[S39–S41] Yet, as discussed further by Edén,[S42] the *assumption* that both PO_4^{3-} and $H_2PO_4^-$ surface species are absent altogether require further verification and unambiguous experimental proof, especially in view of the overall very good accordance between the herein metadynamics-derived models and the dipolar-based NMR experiments for scenarios when the HA-surface model (section **S2.1**) involve significant $H_2PO_4^-$ contributions.

Considering the currently incompletely known chemical compositions at surfaces of pristine ACP and/or nanocrystalline HA particles in contact with an aqueous solution, *surface-models used for computational simulations of biomolecular adsorption lack a well-defined reference.* While the HA surface-preparation protocol introduced by Lin et al.[S19] (and employed by us herein and in ref. S22) does not truly mimic a "fully amorphous" HA surface and also postulates that its $\{H_nPO_4^{(3-n)-}\}$ speciation matches that of an interfacing aqueous solution of phosphate anions at a given pH, it constitutes the hitherto sole attempt toward a reasonably realistic modeling of biomolecular adsorption at nanocrystalline synthetic/biogenic HA particles, where it has for long been highlighted[S43–S46] that computational modeling must consider the by-now well-established structural disorder of the "ACP surface layer" present at all nanocrystalline apatite particles; see ref. 42 for further comments. The recent HA surface-preparation model of ref. S19 is inarguably more realistic than the prevailing mainstream modeling by density functional theory (DFT) calculations or (*ab initio*) MD simulations that assumed strictly periodic HA-crystal surfaces interfacing an aqueous solution[S47–S55] (that could, *at best*, be representative for solutions with pH>14).

The direct constraints from $^{13}C\{^{31}P\}$ and $^{15}N\{^{31}P\}$ REDOR NMR experiments presented herein further corroborate the validity of the HA-surface preparation strategy of Lin *et al.*[S19] Moreover, additional support is provided by the successful prediction of the most stable citrate–HA binding mode from our recent metadynamics simulations,[S22] which accorded well with inferences from solid-state NMR experiments on bone samples[S56]—in stark contrast with conflicting predictions from similar computational modeling using an *ordered* HA-surface model generated by a straightforward crystal cleavage and strictly preserving the lattice periodicity.[S51]

We summarize by highlighting that despite significant challenges of a faithful modeling of biomolecular adsorption at the chemically and structurally complex systems of ACP and synthetic/biogenic nanocrystalline HA, the overall good agreement between the modeled and REDOR NMR-derived dipolar second moments of Table **2** is most gratifying and validate the relevance of the HA-surface preparation model of Lin *et al.*,[S19] as well as the precision of the predictions from our metadynamics simulations.

S3 Synthesis and Characterization of U-[^{13}C, ^{15}N] Pser

S3.1 Materials and Characterizations

All chemical reagents were used as supplied unless otherwise indicated. Trifluoroacetic acid (TFA; CF_3COOH; 99%), tetrazole (0.45 M in MeCN), and hydrogen peroxide (30% in H_2O) solutions were purchased from Sigma Aldrich. Di-*tert*-butyl dicarbonate (Boc_2O; 99%), *tert*-butyl *N*,*N'*-diisopropylcarbamimidate (95%), and di-*tert*-butyl *N*,*N*-diisopropylphosphoramidite (95%) were obtained from Fluorochem (UK). All solvents (analytical or HPLC grade), encompassing the anhydrous ones (Sigma-Aldrich), were used as supplied. Brine refers to a saturated aqueous solution of sodium chloride.

The synthesis of *O*-phospho-L-serine was achieved by the scheme shown in Fig. **S7**, which was adapted from phosphoramidate chemistry, which is commonly employed in solid-phase synthesis of DNA.[S57] Serine termini were protected with TFA sensitive protecting groups, enabling global deprotection in the final preparation (Fig. **S7**).[S58]

[U-$^{13}C/^{15}N$]- L-Serine enriched to 98% of both $^{13}C/^{15}N$ isotopes was purchased from CortecNet (France) and was used for preparing a sample of [U-$^{13}C/^{15}N$]-O-phospho-L-Serine (Pser*). Likewise, a Pser sample with all isotopes at their natural abundance levels was prepared from L-serine (Sigma Aldrich, 98.5%) by an identical synthetic route. Repeated lyophilization from HCl(aq) removed TFA, yielding Pser and Pser* as their HCl salts, Pser·HCl and Pser*·HCl, respectively.

Solution ^{1}H, ^{13}C, and ^{31}P NMR experiments were performed at $B_0 = 9.4$ T with a Bruker Ascend spectrometer that yielded the Larmor frequencies of $\{-400, -100, -162\}$ MHz for $\{^{1}H, ^{13}C, ^{31}P\}$, respectively. The NMR shifts were assigned from ^{1}H–^{1}H COSY, ^{1}H–^{13}C HSQC, and ^{13}C DEPT135 NMR experimentation.[S59] All reported chemical shifts are quoted with respect to the primary standards (section **2.2**) *via* the following solvents as internal standards: $CDCl_3$ at $\delta_C = 77.16$ ppm for ^{13}C, and $CDCl_3$ at $\delta_H = 7.26$ ppm or D_2O at $\delta_H = 4.79$ ppm for ^{1}H. *J*-coupling constants are reported in Hz along with the following splitting abbreviations: singlet (s); doublet (d); multiplet (m), and "br" for "broad" NMR peaks.

Thin layer chromatography (TLC) was performed using aluminium backed sheets coated with 60 F254 silica gel (Merck). The silica plates were inspected by using an UV lamp ($\lambda_{max} = 254$ nm or $\lambda_{max} = 365$ nm), and/or ninhydrin (0.2 w/v-% in ethanol). Flash column chromatography was carried out using a a high-purity silica gel with 0.60 nm pore size and 230–400 mesh-particle size (Fluorochem UK). Mobile phases are reported as % volume of the more polar solvent in the less polar solvent.

Low resolution mass spectra (LRMS) were recorded on an Agilent 6130 Quadrupole mass spectrometer using electrospray ionization (ESI), connected to an Agilent 1260 Infinity liquid chromatography set-up with an ACE3 C8 column (50×3.00 mm) running a gradient of 10-90% MeCN in water containing 0.1% TFA over 1.5 min at a flow rate of 1 mL/min. The UV intensities for LC detection were recorded at wavelengths of 200 nm, 254 nm, and 305 nm.

S3.2 Synthesis Protocol of [U-$^{13}C/^{15}N$]-*O*-phospho-L-serine

S3.2.1 Synthesis of (*tert*-butoxycarbonyl)-L-serine [Product 1]

Each synthesis of molecule **1** (Fig. **S7**), either with or without isotopic enrichment, was performed in two parallel batches of 0.8 g by dissolving either Ser* or Ser (0.8 g, 7.6 mmol, 1.0 equiv.) in 10 mL of 1 M NaOH(aq). The solution was cooled to 0 °C and stirred for 5 min, whereupon 10 mL of di-*tert*-butyl dicarbonate (Boc_2O, 2.0 g, 9.1 mmol, 1.2 equiv.) dissolved in dioxane was added dropwise. The mixture was warmed to room temperature and stirred for 8 h. The mixture was diluted with 50 mL of H_2O and the pH was adjusted to ≈3 by using 1 M $KHSO_4$(aq) (≈10 mL). The aqueous solution was then extracted three times with 70 mL of ethyl acetate. The extracts were combined and washed with brine (70 mL), dried over MgSO4, filtered, and then concentrated *in vacuo* to yield a clear oil (combined weight of 3.3 g). Both products **1** were subsequently used without further purification.

S3.2.2 Synthesis of *tert*-butyl N-(*tert*-butoxycarbonyl)-L-serinate [Product 2]

The synthesis of molecule **2** was performed in two parallel batches: compound **1** (1.7 g, 8.3 mMol, 1.0 eqv.) was dissolved in 50 mL anhydrous dichloromethane, and *tert*-butyl *N*,*N′*-diisopropylcarbamimidate (5.0 mL, 24.8 mmol, 3.0 equiv.) was added dropwise. The mixture

was stirred for 24 h at room temperature, whereupon hexane (100 mL) was added and the mixture was stirred for 10 min. The mixture was cooled to −20 °C and passed through a filter paper. The filtrate was then concentrated *in vacuo*. The residue was purified by flash column chromatography (30% ethyl acetate in n-hexane) to afford product **2** as a light-yellow oil (combined weight 2.3 g, 8.6 mmol, 55.3%).

NMR parameters ($CDCl_3$):

^1H *NMR* : N**H**[5.41 ppm, br, d, ^1J(NH) = 91.0 Hz, 1H]; C**H**[4.25 ppm (br, d, ^1J(CH) = 139.8 Hz, 1H]; C$\mathbf{H}_2$[3.89 ppm, br, d, ^1J(CH) = 146.5 Hz, 2H]; O**H**[2.31 ppm, br, s, 1H]; C(C$\mathbf{H}_3$)$_3$[1.48 ppm, s, 9H); C(C$\mathbf{H}_3$)$_3$[1.45 ppm, s, 9H].

^{13}C *NMR*: ester **C**O[169.87 ppm, d, ^1J(**C**O–**C**H) = 59.8 Hz]; **C**H$_2$[64.33 ppm, d, ^1J(**C**H–**C**H$_2$) = 37.3 Hz]; **C**H[56.46 ppm, ddd, ^1J(**C**H–**C**O) = 59.8 Hz, ^1J(**C**H–**C**H$_2$) = 37.4 Hz, ^1J(**C**H–**N**H) = 13.2 Hz]; C(**C**H$_3$)$_3$[28.45 ppm, s]; C(**C**H$_3$)$_3$[28.13 ppm, d, J = 1.5 Hz];

LRMS: $[M^+H]^+$, $^{13}C_3{}^{12}C_9H_{24}{}^{15}NO_5Na$, 288.1 (observed); 288.1545 (calculated).

S3.2.3 Synthesis of *tert*-butyl N-(*tert*-butoxycarbonyl)-O-(di-*tert*-butoxyphosphoryl)-L-serinate [Product 3]

Molecule **3** of Fig. **S7** was prepared in two parallel batches (1.1 g and 1.5 g) by first dissolving molecule **2** (1.1 g, 4.2 mmol, 1.0 equiv.) in a mixture of anhydrous tetrahydrofuran (50 mL) and tetrazole (0.45 M in MeCN, 18.7 mL, 8.4 mmol, 2.0 equiv.). After cooling the solution to 0 °C, di-*tert*-butyl *N*,*N*- diisopropylphosphoramidite (1.5 mL, 4.6 mmol, 1.1 equiv.) was added dropwise and the mixture stirred overnight at room temperature. Complete consumption of **2** was confirmed by TLC (30% ethyl acetate in n-hexane, stained with ninhydrin). The mixture was cooled to 0 °C and H_2O_2 (30% in H_2O, 0.4 mL, 3.9 mmol, 0.9 equiv.) was added dropwise. The solution was then stirred for 2 h. Consumption of the intermediate was confirmed by TLC before quenching the reaction by addition of 10 mL $Na_2S_2O_3$(aq) (5% w/v). The solvent was then removed *in vacuo* and the residue was redissolved in 50 mL of dichloromethane. The products were washed with H_2O (50 mL) and brine (50 mL), then dried over $MgSO_4$ and filtered, and subsequently concentrated *in vacuo*. The residue was purified twice by flash column chromatography (30% ethyl acetate in n-hexane), yielding **3** as an off-white solid (combined weight 2.0 g, 4.52 mmol, 52%).

NMR parameters ($CDCl_3$):

^1H *NMR*: N**H**[5.52 ppm, dd, ^1J(NH) = 92.0 Hz, ^3J(N**H**–C**H**) = 8.5 Hz, 1H]; C**H**/C$\mathbf{H}_2$[4.55–3.90 ppm, m, 3H]; C(C$\mathbf{H}_3$)$_3$[1.48 ppm, d, ^4J(C$\mathbf{H}_3$–**P**) = 0.5 Hz, 9H); C(C$\mathbf{H}_3$)$_3$[1.47 ppm, s, 9H]; C(C$\mathbf{H}_3$)$_3$[1.46 ppm, d, ^4J(C$\mathbf{H}_3$–**P**) = 0.5 Hz, 9H]; C(C$\mathbf{H}_3$)$_3$[1.44 ppm, s, 9H];

^{13}C *NMR*: **C**O[168.74 ppm, d, ^1J(**C**O–**C**H) = 61.0 Hz]; **C**H$_2$[67.19 ppm, dd, ^1J(**C**H$_2$–**C**H) = 40.0 Hz, ^2J(**C**H$_2$–**P**) = 5.5 Hz]; **C**H[54.56 ppm, dddd, ^1J(**C**H–**C**O) = 61.0 Hz, ^1J(**C**H–**C**H$_2$) = 40.0 Hz, ^3J(**C**H–**P**) = 13.6 Hz, ^2J(**C**H–**N**H) = 8.5 Hz]; C(**C**H$_3$)$_3$[29.93 ppm, d, ^4J(**C**H–**P**) = 1.9 Hz; C(**C**H$_3$)$_3$[29.98 ppm, d, ^4J(**C**H$_3$–**P**) = **1.9** Hz; C(**C**H$_3$)$_3$[28.46 ppm, s]; C(**C**H$_3$)$_3$[28.11 ppm, d, ^4J(**C**H–**C**H$_3$) = 1.1 Hz].

S3.2.4 Conversion of Product 3 Into *O*-phospho-L-serine

The synthesis was performed in two parallel batches of 1.5 g and 0.5 g. Compound **3** (1.5 g, 3.3 mmol, 1.0 equiv.) was cooled to 0 °C in an ice bath and 4.0 mL TFA was added slowly while stirring. The solution temperature was then increased to room temperature and stirred for 3 h. The solvent was removed *in vacuo* and 6.0 mL of HCl (2 M in diethyl ether) was added. The reaction was stirred for 5 min and the solvent was then removed *in vacuo*, whereupon HCl (2 M in H_2O, 8 mL) was added. The solution was filtered through glass filter paper and lyophilized. The crude product was redissolved in HCl (2 M in H_2O, 8 mL), filtered and lyophilized once more to

remove traces of trifluoroacetic acid. This yielded the HCl salt of either Pser or Pser* (product **4** of Fig. **S7**) as an off-white, hygroscopic powder (combined weight 0.930 g, 4.2 mmol, 95%).

NMR parameters for Pser (D_2O at pH 7.4):

^1H *NMR*: C**H**H′[4.25 ppm, ddd, ^2J(**H**–C**H**′) = 11.4 Hz, ^3J(**H**–**P**) = 5.1 Hz, ^3J(**H**–C**H**) = 3.4 Hz, 1H]; CH**H**′[4.19 ppm, ddd, ^2J(**H**′–C**H**) = 11.4 Hz, ^3J(**H**′–**P**) = 5.7 Hz, ^3J(**H**′–C**H**) = 5.9 Hz, 1H]; C**H**[3.99 ppm, ddd, ^3J(C**H**–CH**H**′) = 5.9 Hz, ^3J(C**H**–C**H**H′) = 3.4 Hz, ^4J(C**H**–**P**) = 1.2 Hz, 1H).

^{13}C *NMR:* **C**OOH [169.67 ppm, s]; **C**H_2[62.88 ppm, d, ^2J(**C**H_2–**P**) = 4.7 Hz]; **C**H[53.64 ppm, d, ^2J(**C**H–**P**) = 8.7 Hz].

^{31}P *NMR:* **P**O_4[−0.33 ppm, s].

LRMS: $[M^+H]^+$, $^{12}C_3H_9{}^{14}NO_6P$, 186.0 (observed); 186.0168 (calculated).

NMR parameters for Pser* (D_2O at pH 7.4):

^1H *NMR*: C**H**/C**H**$_2$[4.59–3.98 ppm, m, br, 3H].

^{13}C *NMR:* **C**OOH [169.22 ppm, d, ^1J(**C**O–**C**H) = 59.3 Hz]; **C**H_2[62.86 ppm, dd, ^1J(**C**H_2–**C**H) = 38.5 Hz, ^2J(**C**H_2–**P**) = 4.0 Hz]; **C**H[53.28 ppm, dddd, ^1J(**C**H–**C**O) = 59.3 Hz, ^1J(**C**H–**C**H_2) = 38.5 Hz, ^3J(C**H**–**P**) = 8.0 Hz, ^2J(C**H**–**N**H) = 8.0 Hz].

^{31}P *NMR:* **P**O_4[−0.37 ppm, dd, ^2J(**P**–CH_2) = 8.0 Hz, ^2J(**P**–**C**H) = 4.0 Hz]).

LRMS: $[M^+H]^+$, $^{13}C_3H_9{}^{15}NO_6P$, 190.0 (observed); 190.0238 (calculated).

S3.3 Solution-State NMR Features of Pser*/Pser

The ^{1}H, ^{13}C, and ^{31}P chemical shifts of Pser and Pser* accorded well with prior literature.[S60,S61] Besides expected differences in additional J-splitting patterns stemming from the presence of both $^{13}C/^{15}N$ isotopes (98%) in Pser*, the observed NMR spectra of the Pser/Pser* products were similar, overall confirming successful preparations. For instance, the ^{1}H NMR spectrum of Pser* shown in Fig. **S8** reveal additional splittings of the CH and CH_2 resonances from $^1J(^{13}C–^1H)$ interactions, whose respective magnitudes of ≈149 Hz and ≈155 Hz are consistent with ref. S60. The additional smaller splittings (1–2 Hz) of these ^{1}H NMR signals likely stem from $J(^{15}N–^1H)$ couplings,[S62,S63] but ^{1}H NMR-peak broadenings prevented their unambiguous assignment. Notably, no couplings of this magnitude were observed in the spectrum from Pser (Fig. **S8**), but ^3J(C**H**$_2$–**P**) coupling constants of 5.1 Hz and 5.7 Hz along with ^4J(C**H**–**P**) = 1.2 Hz are discernible, in agreement with previous results.[S64]

The {^{13}CO, ^{13}CH, $^{13}CH_2$} chemical shifts of {169.22, 62.86, 53.28} ppm (Pser*) and {169.67, 62.88, 53.64} ppm (Pser) observed in the presence of proton decoupling (Fig. **S9**a) accord well with those of ref. S60 (within experimental uncertainties). The 98% ^{13}C enrichment of Pser* renders its ^{13}C resonances split in doublets by the homonuclear $^1J(^{13}C–^{13}C)$ interactions (Fig. **S9**a), whose magnitudes ^1J(**C**O–**C**H) = 59.3 Hz and ^1J(**C**H–**C**H_2) = 38.5 Hz are similar to the corresponding values of 59.2 Hz and 36.4 Hz reported for [U-^{13}C]-enriched L-serine.[S65] (As anticipated, these large splittings are absent in ^{13}C NMR spectrum from Pser in Fig. **S9**a). The ^{13}CH NMR signal (δ_C = 53 ppm) from the CH group of Pser* reveals a complex multiplet (Fig. **S9**a), which besides the above-mentioned large splittings from $^1J(^{13}C–^1H)$ interactions involve two smaller splittings associated with J ≈8 Hz: one of them is attributed to ^3J(**C**H–**P**) interactions[S60] on the basis of the very similar splitting observed for the ^{13}CH resonances from Pser, whereas the other one—*not* observed from Pser—is assigned to the ^1J(**C**H–**N**) coupling between the ^{15}**N**H_3 and ^{13}**C**H sites, thereby confirming the presence of ^{15}N in the Pser* molecules. The value ^1J(**C**H–**N**) = 8.0 Hz, the "dddd" splitting pattern of the ^{13}CH signal, and the absence of further splittings from longer-range J interactions are all consistent with this interpretation.[S66]

The ^{31}P NMR spectra of Pser and Pser* are shown in Fig. **S9**b. The ^{31}P chemical shift ($\delta_P \approx -0.35$ ppm) is similar to those of other phosphorylated amino acids and phosphoseryl residues in peptides.[S61,S64] The $^3J(\mathbf{P}\text{–}\mathbf{C}\text{H}) = 8.0$ Hz and $^2J(\mathbf{P}\text{–}\mathbf{C}\text{H}_2) = 4.0$ Hz coupling constants also manifest in the ^{31}P NMR spectrum from Pser* (as anticipated), whereas the ^{31}P resonance from Pser remains as a singlet due to its absence of ^{13}C enrichment.

The successful Pser* preparation was further confirmed by LC-MS results, where the $[M^{+}H]^{+}$ peak (190.0) suggested a molecular formula of $^{13}C_3H_9{}^{15}NO_6P$ (calculated: 190.0238), whereas Ser revealed a peak at 186.0 from $^{12}C_3H_9{}^{14}NO_6P$ (calculated: 186.0168).

S4 The ^{1}H NMR Signature of Structural Water in ACP

Owing to the significantly diminished ^{1}H–^{31}P dipolar interactions of the mobile physisorbed H_2O molecules, their resonances are not detected in our ^{1}H{^{31}P} D-HMQC NMR experiments. In contrast, the correlation peak at $\{\delta_P, \delta_H\} \approx \{1.0, 7.2\}$ ppm observed from the Pser16 cement (Fig. **4**b) was previously attributed to structure-bound water molecules present in the ACP/Pser component.[S9] This inference was strengthened further by subsequent findings from ^{1}H{^{31}P} D-HMQC NMR of several pristine ACP[S67] and nanocrystalline HA[S68] samples as a *general* structural feature of "ACP". Further support is offered by a paper in press from Chan's group[S69] that appeared during the revision of the present paper: those authors also attributed ^{1}H resonances in the 6.4–6.8 ppm range to structure-bound water molecules in various ACP samples prepared under different conditions.[S69]

The overall minor structure-bound H_2O component in ACP out of the total proton reservoir in the present cement/Pser@HA specimens makes the $\delta_H \approx 7$ ppm resonance swamped by those of organic protons and physisorbed water in the single-pulse-acquired ^{1}H MAS NMR spectra of Fig. **3**, notwithstanding that the signal is clearly evident from similar experiments on pristine/bulk ACP phases.[S67] Yet, the ^{1}H{^{31}P} correlation involving structural water molecules is indeed also hinted by a 2D peak at $\{\delta_P, \delta_H\} \approx \{1.8, 7.2\}$ in the D-HMQC spectrum from Pser@HA in Fig. **4**c; its lower intensity is naturally rationalized from the comparatively lower amount of "ACP" present at the nanocrystalline HA particle-surface relative to that of pristine/bulk ACP or the ACP/Pser component of the Pser16 cement. The lower hydrophilicity of the Ser*N* particle-surface due to the absence of an organic phosphate group of Ser presumably renders the $\delta_H \approx 7$ ppm resonance undetected from Ser*N* cements (such as the present Ser16 sample; see Fig. **4**a) *but* those incorporating very low amounts of Ser; see for instance the ^{1}H–^{31}P NMR correlation results from a "Ser8" cement in ref. S9.

We conclude that the ^{1}H NMR peak with $\delta_H \approx 7$ ppm is a "universal fingerprint" of structure-bound water molecules in "ACP", encompassing (*i*) pristine ACP, (*ii*) the amorphous surface layer of nanocrystalline HA, as well as (*iii*) the organic/inorganic ACP/Pser and ACP/Ser components of CaP biocements. Moreover, while such structure-bound water molecules constitute an integral component of ACP phases, the precise protonation states of their nearby phosphate groups are partially dictated by the pH value of the solution from which "ACP" formed, which controls its ratio of protonated and non-protonated phosphate groups.[S9,S42,S67,S68] Consequently, $H_2O\cdots PO_4^{3-}$ motifs prevail in pristine ACP prepared under *alkaline* conditions, which is rich in H_2O and *non*-protonated phosphate groups, as reflected by correlated shift-pairs of $\{\delta_P, \delta_H\} = \{2.9, 6.9\}$ observed by ^{31}P{^{1}H} HETCOR NMR and $\{\delta_P, \delta_H\} = \{2.4, 7.2\}$ by ^{1}H{^{31}P} D-HMQC NMR in ref. 67 [1]

[1] As discussed in ref. S67, lower ^{31}P chemical shifts are observed in ^{1}H{^{31}P}HMQC experiments (here, by ≈0.5 ppm) relative to those of ^{31}P{^{1}H} HETCOR from the same sample, which we attribute to the active ^{1}H–^{1}H decoupling accomplished by the $SR4_1^2$ scheme[S6] used for the HMQC generation, which quenches proton-driven spin-diffusion

In contrast, the ACP surface layer of the present Pser@HA particles (prepared at pH=5.3)—as well as the ACP/Pser phases of Pser16—comprise lower structural water contents but significantly higher amounts of protonated phosphate moieties. Hence, these "ACP" structures involve primarily $H_2O\cdots HPO_4^{2-}$ and/or $H_2O\cdots H_2PO_4^{-}$ motifs, as reflected by the lower ^{31}P chemical shifts in the range 1.0–1.8 ppm observed in the HMQC NMR spectra of Fig. **4**b,c and in ref. S9.

from longer-range ^{1}H sites, thereby emphasizing the correlations from shorter-range ^{1}H–^{31}P pairs of acidic-phosphate moieties.

Tables

Table S1. Cement Batch Compositions.[a]

Sample	Ser/Pser (mol %)	Ser/Pser (wt %)	pH[b]
Ser8	8.0	3.0	7.30
Ser16	16.0	6.0	7.13
Pser8	8.0	5.0	4.26
Pser16	16.0	10.0	3.85
Pser30	30.0	20.0	3.32

[a]Mol % and wt % of $^{13}C/^{15}N$ enriched Ser* and Pser* precursors in the respective Ser$N$ and Pser$N$ cement batch, with the remaining constituting α-$Ca_3(PO_4)_2$. All samples were prepared with a total of 250 mg powder, to which 60 μL distilled water was added (L/P=0.24). All Pser$N$ cements were prepared from the Pser* powder, which comprised >95% of Pser·HCl (e.g., see Fig. **9**a and **S1**). All preparations accounted for the mass contribution from Cl.

[b]pH value ($\pm$0.02) of the cement paste prior to setting. Cements prepared from α-TCP and water alone featured pH=8.0. Note that a higher ratio L/P=0.40 mL/g was needed for enabling pH measurements (section **2.1**) relative to that of L/P=0.24 mL/g employed for the isotopically enriched cement preparations. Conservative pH calculations predict that the *actual* pH value for a given Ser$N$ and Pser$N$ cement may be $\leqslant$0.1 (Ser8/Ser16) and $\leqslant$0.25 *lower* (for Pser30), respectively, than that stated in the Table. Hence, this feature is not expected to have any significant bearings on the NMR or modeling results; see section **S2.1**.

Table S2. Partial Charges Used for the Metadynamics Simulations and Debye–Hückel Analyses.[a]

group	q_{O}	q_{OH}	q_{H}	$q_{\boldsymbol{X}}$
Ser (ref. S70)				
NH_3^+			0.330	−0.300
CH			0.100	0.210
CH_2			0.090	0.050
OH		−0.660	0.430	
COO^-	−0.670			0.340
Pser (ref. S71)				
NH_3^+			0.330	−0.300
CH			0.100	0.210
CH_2O	−0.620		0.090	−0.080
PO_3^{2-}	−0.900			1.100
HPO_3^-	−0.820	−0.680	0.340	1.500
COO^-	−0.670			0.340
HA (ref. S19)				
PO_4^{3-}	−0.800			1.000
(100)–HPO_4^{2-}	−0.783	−0.650	0.400	1.000
(001)–HPO_4^{2-}	−0.750	−0.650	0.400	1.000
(100)–$H_2PO_4^-$	−0.675	−0.650	0.400	1.000
(001)–$H_2PO_4^-$	−0.625	−0.650	0.400	1.000
OH^-		−1.100	0.200	
Ca^{2+}				1.500
Solution (ref. S72)				
H_2O		−0.834	0.417	
Na^+				1.000

[a]Partial charge q_E of atom type E, which may be present either at the organic molecule, at each (100) or (001) HA surface or lattice, or at water molecules. $\boldsymbol{X}$ in the rightmost column represents either of {C, N, P}.

Table S3. Adsorption Energies for the Pser and Ser Binding at HA (kJ/mol).[a]

	Pser				Ser			
Surface	(100)		(001)		(100)		(001)	
pH	4.5	7.4	4.5	7.4	4.5	7.4	4.5	7.4
$-\Delta F_{\mathrm{ads}}(\pm 5)$	40.7	66.4	33.1	49.6	36.6	37.7	22.0	23.3
$-\Delta U_{\mathrm{ads}}(\pm 5)$	32.5	58.3	24.9	41.5	28.4	29.6	13.9	15.2
$-E_{\mathrm{DH}}^{\mathrm{tot}}$	**33.9**	**57.8**	**21.6**	**41.0**	**27.3**	**29.7**	**14.6**	**14.7**
**Ca**	28.8	52.2	18.1	25.7	15.9	22.3	8.2	10.7
$\mathbf{H}_n\mathrm{PO}_4$	1.5	3.3	0.7	14.8	2.0	1.0	3.4	1.4
$\mathrm{H}_n\mathrm{P}\mathbf{O}_4$	3.5	2.4	2.8	0.5	9.5	6.4	3.0	2.6
P**O**[b]	**23.6**	**40.7**	**17.0**	**31.1**				
P**O**–**Ca**	22.4	38.4	16.9	16.7				
P**O**–$\mathbf{HPO_4}$				0.5				
P**O**–$\mathbf{H_2}\mathrm{P}\mathbf{O}_4$	1.2	2.3	0.2	13.9				
C**O**[c]	**6.8**	**14.8**	**1.8**	**9.4**	**15.6**	**22.1**	**11.5**	**11.8**
C**O**–**Ca**	6.4	13.8	1.3	9.0	13.7	21.2	8.2	10.5
C**O**–**H**PO_4				0.3				0.2
C**O**–$\mathbf{H}_2\mathrm{PO}_4$	0.3	1.0	0.6		1.9	0.8	3.3	1.1
N**H**[d]	**3.0**	**2.4**	**2.6**	**0.5**	**8.1**	**4.7**	**2.4**	**2.3**
N**H**–$\mathrm{P}\mathbf{O}_4$	2.5	1.9			7.8	2.1	0.2	
N**H**–$\mathrm{HP}\mathbf{O}_4$				0.5		2.1		1.5
N**H**–$\mathrm{H_2P}\mathbf{O}_4$	0.5	0.4	2.6		0.3	0.5	2.2	0.8
C**OH**[d]					**3.6**	**2.9**	**0.7**	**0.6**
C**O**H–**Ca**					2.2	1.1		0.2
CO**H**–$\mathrm{P}\mathbf{O}_4$					1.3	0.2		
C**OH**–$\mathbf{HPO_4}$						1.2		0.2
C**OH**–$\mathbf{H}_2\mathrm{P}\mathbf{O}_4$						0.4	0.7	
PO**H**[d]	**0.5**		**0.3**					
PO**H**–$\mathrm{P}\mathbf{O}_4$	0.3		0.2					
PO**H**–$\mathrm{H_2P}\mathbf{O}_4$	0.2							

[a]Helmholtz free energy (ΔF_{ads}; eq. S8) and internal energy (ΔU_{ads}; eq. S10) of adsorption, along with the total Debye-Hückel energy ($E_{\mathrm{DH}}^{\mathrm{tot}}$; eq. S13) and its net contribution from **Ca**, $\mathbf{H}_n\mathrm{PO}_4$, and $\mathrm{H}_n\mathrm{P}\mathbf{O}_4$ groups listed beneath, where the interacting A and B species are typeset in boldface. Empty entries involve energies with magnitudes < 0.2 kJ/mol. The uncertainties of all DH energies are $\pm 12\%$. The very good agreement $E_{\mathrm{DH}}^{\mathrm{tot}} \approx \Delta U_{\mathrm{ads}}$ justifies our DH-energy based analysis on the individual A–B interactions, $E_{\mathrm{DH}}(A\text{–}B)$ [eq. S11].

[b]Net DH energy of the organic phosphate group, which is the sum over the $\{E_{\mathrm{DH}}(\mathrm{P}\mathbf{O}\text{–}B)\}$ contributions from electrostatic/H-bond interaction energies listed beneath for each of $B = \{$**Ca**, **H**PO_4, $\mathbf{H}_2\mathrm{PO}_4\}$.

[c]Net DH energy of the carboxy group, which is the sum over the $\{E_{\mathrm{DH}}(\mathrm{C}\mathbf{O}\text{–}B)\}$ contributions from electrostatic/H-bond interactions listed beneath for each of $B = \{$**Ca**, $\mathbf{H}\mathrm{PO}_4$, $\mathbf{H}_2\mathrm{PO}_4\}$.

[d]Net DH energy of the amino group, the hydroxyl moiety of Ser, or the HPO_4^- group of Pser, along with their respective energy contributions listed beneath. Note that these groups may enter a "pocket" at the surface, which enables H bonds to atoms of inorganic phosphate groups located beneath the outermost surface layer.

Table S4. Number of Bonds Between Pser/Ser and the HA Surface.[a]

	Pser				Ser			
Surface	(100)		(001)		(100)		(001)	
pH	4.5	7.4	4.5	7.4	4.5	7.4	4.5	7.4
Z_{tot}	**5.80**	**8.53**	**4.22**	**7.45**	**6.76**	**6.20**	**3.94**	**3.25**
**Ca**	3.90	6.63	2.33	3.26	2.48	3.48	1.29	1.65
**H**$_n$PO$_4$	0.49	0.96	0.26	3.99	0.77	0.36	1.28	0.50
H$_n$P**O**$_4$	1.41	0.94	1.64	0.21	3.51	2.36	1.37	1.10
P**O**[b]	**3.26**	**5.07**	**2.19**	**5.73**				
P**O**–**Ca**	2.91	4.47	2.14	1.91				
P**O**–**H**PO$_4$				0.12				
P**O**–**H**$_2$PO$_4$	0.36	0.61	0.05	3.70				
C**O**[c]	**1.13**	**2.52**	**0.40**	**1.51**	**2.85**	**3.61**	**2.51**	**2.09**
C**O**–**Ca**	1.00	2.16	0.19	1.35	2.11	3.29	1.28	1.61
C**O**–**H**PO$_4$				0.11				0.09
C**O**–**H**$_2$PO$_4$	0.13	0.35	0.21	0.05	0.75	0.30	1.23	0.39
N**H**[d]	**1.15**	**0.94**	**1.58**	**0.21**	**3.12**	**1.82**	**1.16**	**1.01**
N**H**–P**O**$_4$	0.93	0.73			2.99	0.81	0.09	
N**H**–HP**O**$_4$		0.03		0.19		0.77		0.59
N**H**–H$_2$P**O**$_4$	0.22	0.19	1.58		0.13	0.24	1.06	0.38
C**OH**[d]					**0.79**	**0.77**	**0.27**	**0.15**
C**O**H–**Ca**					0.37	0.19	0.01	0.04
CO**H**–P**O**$_4$					0.37	0.06		
C**OH**–**HPO**$_4$						0.38		0.07
C**OH**–**H**$_2$P**O**$_4$						0.13	0.26	
PO**H**[d]	**0.25**		**0.06**					
PO**H**–P**O**$_4$	0.15		0.05					
PO**H**–H$_2$P**O**$_4$	0.11							

[a]Number of bonds, i.e., the coordination number $Z(A\text{–}B)$ associated with atom A of the Ser or Pser molecule and species B at the HA surface, where A and B are typeset in bold-face. Z_{tot} denotes the total number of electrostatic/H bond interactions, whose contributions from B ={**Ca**, **H**$_n$PO$_4$, H$_n$P**O**$_4$} are listed beneath. The uncertainty of each $Z(A\text{–}B)$ value is ±12%. Empty entries correspond to insignificant interactions (Table **S3**) that typically involve $Z(A\text{–}B) < 0.02$.

[b]Net coordination number of the phosphate group, which is the sum over {Z(P**O**–B)} contributions from the interactions listed beneath for each of B = {**Ca**, **H**PO$_4$, **H**$_2$PO$_4$}.

[c]Net coordination number of the carboxy group, which is the sum over {Z(C**O**–B)} contributions from the interactions listed beneath for each of B = {**Ca**, **H**PO$_4$, **H**$_2$PO$_4$}.

[d]Net coordination number of the amino group, the hydroxyl moiety of Ser, or the HPO_4^- group of Pser, along with their respective bond contributions listed beneath. Note that these groups may enter a "pocket" at the surface, which enables H bonds to atoms of inorganic phosphate groups located beneath the outermost surface layer.

Table S5. Ratios of Experimental and Calculated/Modeled Dipolar Second Moments.[a]

System	rM_2(N–P)	rM_2(CO–P)	rM_2(CH–P)	rM_2(CH_2–P)
Ser16	0.56	0.79	0.68	0.82
Pser16	0.54	0.95	0.71	0.78
Pser@HA	0.77	1.14	0.81	0.76
Pser	0.71	0.76	0.76	0.78
CaPser	–	0.76	0.73	0.77

[a]Ratio $rM_2 = M_2^{\text{exp}}/M_2^{\text{calc}}$ for the as-indicated M_2(N–P) and M_2(C–P) data listed in Table **2**.

Table S6. Best-Fit ^{31}P NMR Parameters From Spectra Deconvolutions.[a]

Sample/Component	δ_P (ppm)	fwhm (ppm)	fraction
**Pser@HA (Fig. 2a)**			
ACP	2.2	5.19	0.39
HA	3.0	1.64	0.61
**Pser@HA (Fig. 2b)**			
ACP	2.0	5.76	0.65
HA	3.0	1.65	0.35
**Ser8 (Fig. 2d)**			
ACP/Ser	2.3	5.07	0.69
HA	2.9	1.43	0.30
Peak* [b]	−0.3	0.56	0.01
**Ser16 (Fig. 2f)**			
ACP/Ser	2.4	5.14	0.82
HA	3.0	2.01	0.18
**Pser8 (Fig. 2h)**			
ACP/Pser	1.8	5.35	0.94
brushite	1.4	0.41	0.06
**Pser16 (Fig. 2j)**			
ACP/Pser	1.5	5.36	0.93
brushite	1.4	0.42	0.07
**Pser30 (Fig. 2l)**			
ACP/Pser	0.7	5.14	0.95
brushite	1.4	0.43	0.04
CaPser	−1.0	0.46	0.01

[a]Best-fit parameters obtained by deconvoluting the ^{1}H→^{31}P CPMAS NMR spectra of Fig. **2**, along with the single-pulse-derived spectrum of Pser@HA. Using software developed in our lab, each spectrum was deconvoluted into the as-indicated phases (also see Fig. **2**), whose associated values of the fractional populations, (average) ^{31}P chemical shifts (δ_P), and full width at half maximum (fwhm) height are specified. The data uncertainties of all narrow peaks (fwhm <1.7 ppm) are ±0.1 ppm for both δ_P and fwhm, whereas the broad ACP/Pser and ACP/Ser peaks have uncertainties of ±0.2 ppm for δ_P and ±0.25 ppm for the fwhm. The uncertainties of the fractional populations are ±0.02 for the ACP and HA components in the Pser@HA, Ser8, and Ser16 samples, whereas they are ±0.01 for all phases in the Pser$N$ specimens.

[b]Unknown but minute crystalline impurity phase marked by an asterisk in Fig. **2**d.

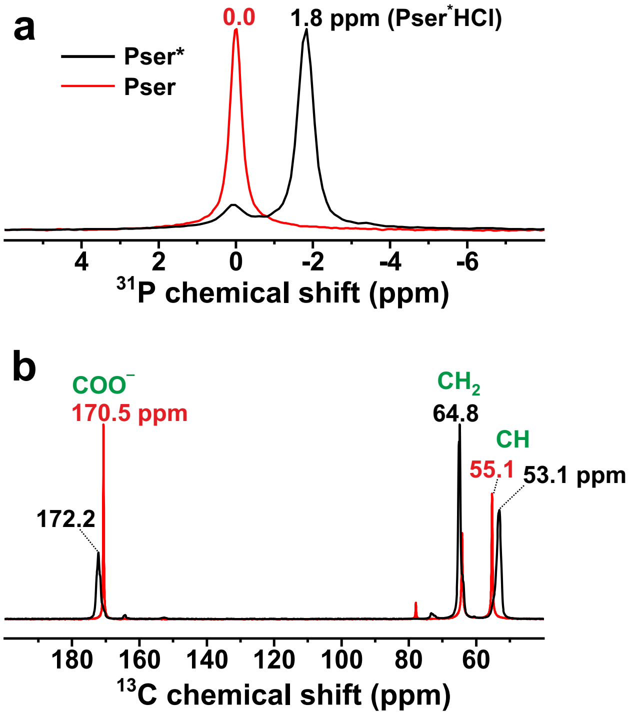

Fig. S1. (a) ^{1}H→^{13}P and (b) ^{1}H→^{13}C CPMAS NMR spectra (red traces) recorded from the herein synthesized Pser sample (98% ^{13}C/^{15}N enrichment, shown together with the corresponding NMR spectra from the well-crystalline Pser powder (Flamma SpA; ^{13}C at natural abundance); the latter spectra (black traces) are those presented in Figs. **6** and **S4**. Note that the "Pser*" powder consists predominantly by poorly ordered Pser*·HCl (whose crystal structure and NMR shifts data appears to be hitherto unpublished), along with a minor Pser* component. Hence, the NMR peak at $\delta_P = 1.8$ ppm in (a) is attributed to Pser*·HCl, while the minor peak at 0.0 ppm originates from the ^{13}C/^{15}N enriched Pser molecules. Likewise, the broad ^{13}C resonances in the black trace in (b) are attributed to Pser*·HCl: while the chemical shift of the $^{13}CH_2$ peak are nearly identical between Pser and Pser*·HCl, they differ by around 2 ppm among the ^{13}COOH and ^{13}CH sites. We stress that the use of the Pser*·HCl precursor had no practical bearings for the isotopically enriched Pser$N$ and Pser@HA specimens because the Cl^- anions remain inert, as corroborated by the similarity of the ^{31}P (Fig. **2**) and ^{13}C (Fig. **6**) NMR spectra from the Pser$N$ cements and those prepared previously from Pser (Flamma, SpA); see ref. S9. Note that the absence of a resonance at $\delta_P \approx 1.8$ ppm in the NMR spectra of Fig. **2** confirms that neither Pser@HA nor any Pser$N$ cement comprise unreacted Pser*·HCl.

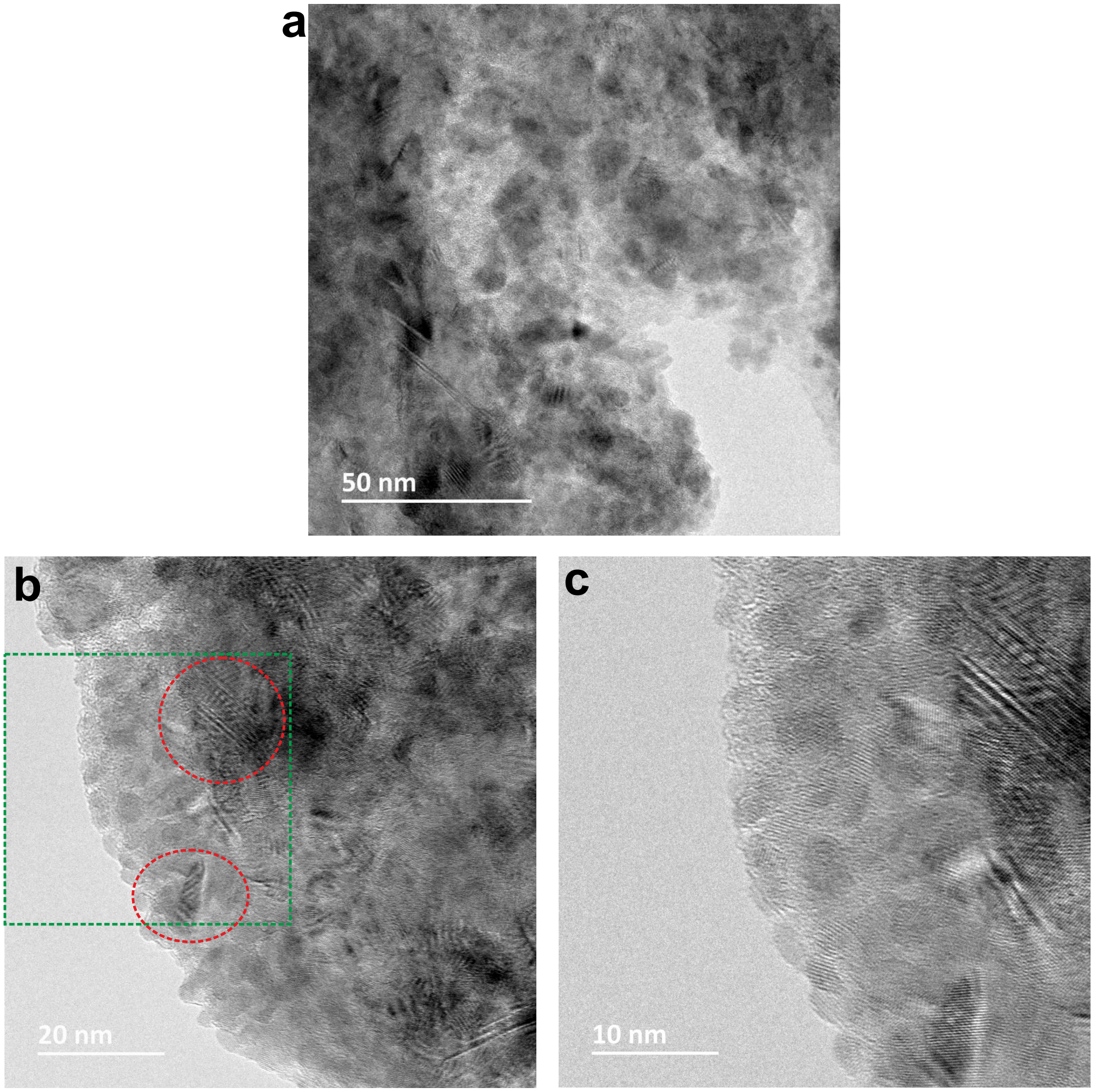

Fig. S2. Transmission electron microscopy (TEM) images of Pser@HA particles shown at different magnifications. Each "particle" consists of an agglomerate of alternating amorphous and crystalline domains that are fused together. The crystalline domain-sizes varied between a few nm up to around 10–20 nm. The red circles in (b) marks two such crystalline areas, which are more evident in the zoomed area shown in (c).
Experimental conditions: The Pser@HA powder was applied directly onto an carbon film supported by copper mesh (Lacy Carbon film supported on a 200 mesh grid, from TED Pella) under dry condition and examined by a JEM 2100F instrument (JEOL, Japan) operating at 200 kV. The images were recorded with a CCD camera connected to the Digital Micrograph program package from Gatan.

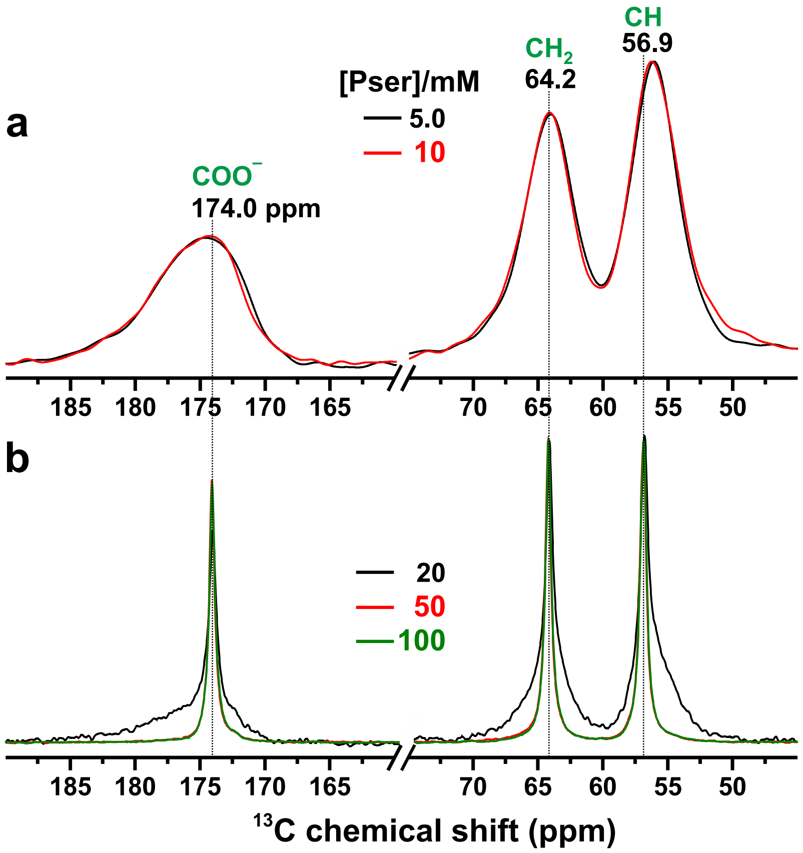

Fig. S3. $^1H \rightarrow ^{13}C$ CPMAS NMR spectra obtained at 9.4 T and 9.00 kHz MAS from Pser@HA specimens prepared as described in section **2.1**, but employing a Pser precursor with ^{13}C at natural abundance and various Pser concentrations of (a) 5.0 mM and 10.0 mM, and (b) {20, 50, 100} mM. The full width at half height (fwhm) of the ^{13}C NMR peaks are markers of surface-bound Pser molecules, where the preparations with low concentrations [Pser]$\leqslant$10 mM resulted in a complete Pser surface-immobilization and fwhm values of $\approx$7.6 ppm for the $^{13}COO^-$ resonance and $\approx$4.5 ppm for the aliphatic peaks. In contrast, the narrow ^{13}C NMR peaks (fwhm $\approx$ 0.6 ppm) stemming from crystalline CaPser in (b) (also see Fig. **6**a) dominated the samples prepared with [Pser]={50, 100} mM. The NMR spectrum recorded from the apatite sample obtained with [Pser]=20 mM comprise both narrow resonances from CaPser and broad signals from the surface-bound Pser molecules. Note that the Pser content varies among the samples and that all NMR spectra are presented with equal maximum amplitudes.

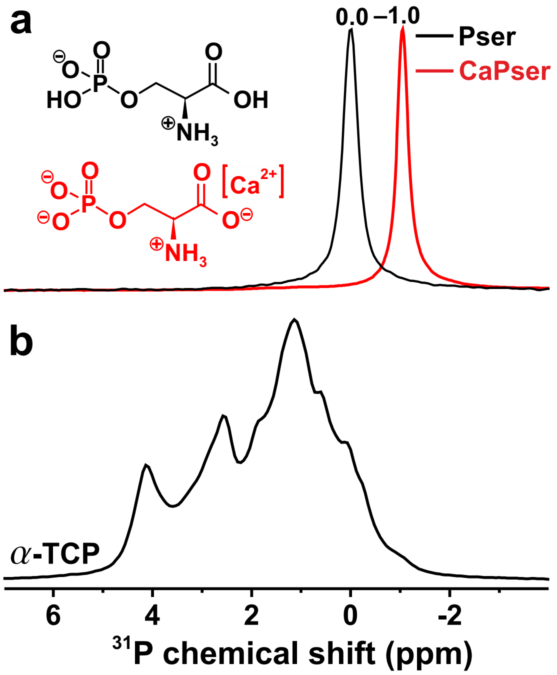

Fig. S4. ^{31}P MAS NMR spectra recorded by single pulses from (a, b) well-crystalline precursors of (a, b) *O*-phospho-L-serine (Pser) and Ca[*O*-phospho-L-serine]·H_2O (CaPser),[S9] and (b) α-$Ca_3(PO_4)_2$ (α-TCP). The spectra in (a) are reproduced from raw data presented in ref. S73.

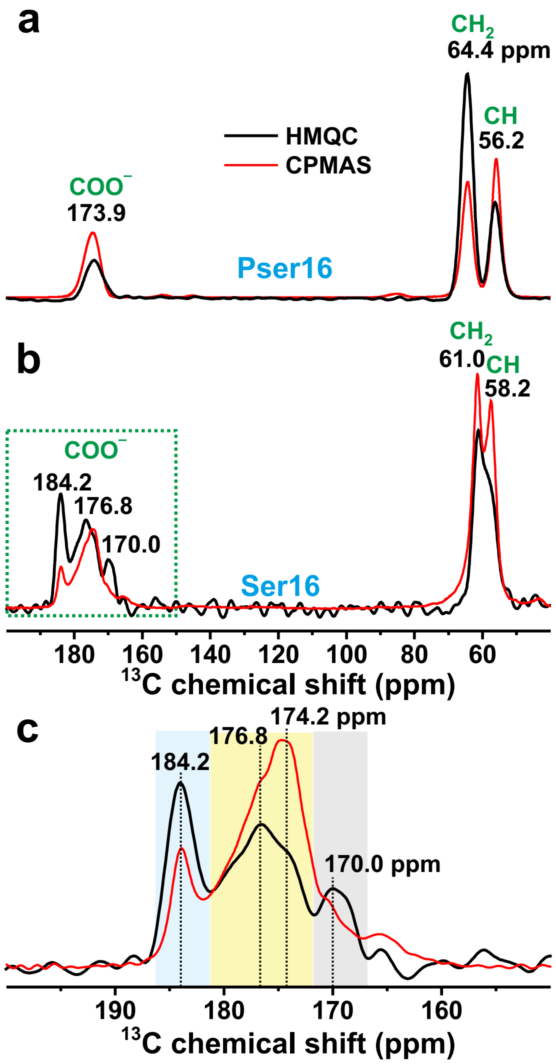

Fig. S5. Projections along the ^{13}C dimension of the ^{13}C{^{31}P} D-HMQC NMR spectra of Fig. **7** from the (a) Pser16 and (b) Ser16 specimens, normalized to a unity integrated spectral intensity, and shown together with their ^{1}H→^{13}C CPMAS NMR spectra (red traces). The spectra in (c) are zoomed across the ^{13}C shift-region of the carboxy groups. The HMQC projection as well as the MAS spectra were renormalized to a unity integrated intensity across the ppm-region displayed in (c).

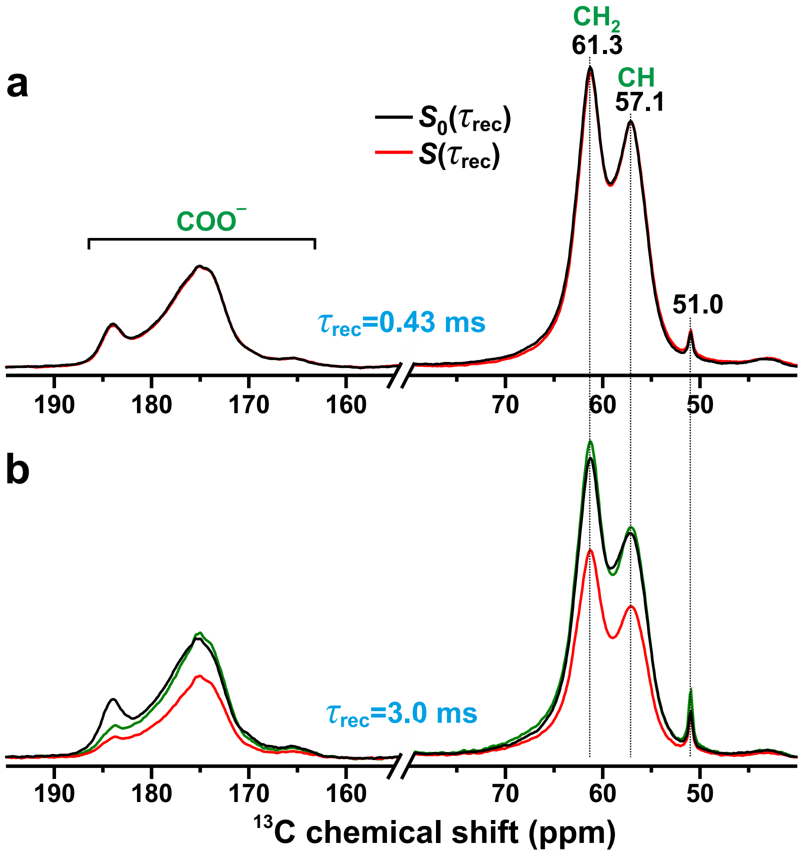

Fig. S6. ${}^{13}C\{{}^{31}P\}$ REDOR NMR spectra, $S(\tau_{\rm rec})$, and their "reference" counterparts $S_0(\tau_{\rm rec})$, obtained from the Ser16 cement and dipolar dephasing/decoupling periods of (a) $\tau_{\rm rec} = 0.429$ ms and (b) $\tau_{\rm rec} = 3.00$ ms. The green curve in (b) represents the REDOR NMR spectrum after normalization such that its integrated signal intensity matches that of the reference experiment [S_0(3.00 ms)]. The very narrow peak at 51 ppm stems from a minute but unknown impurity phase, which does not bind to ACP.

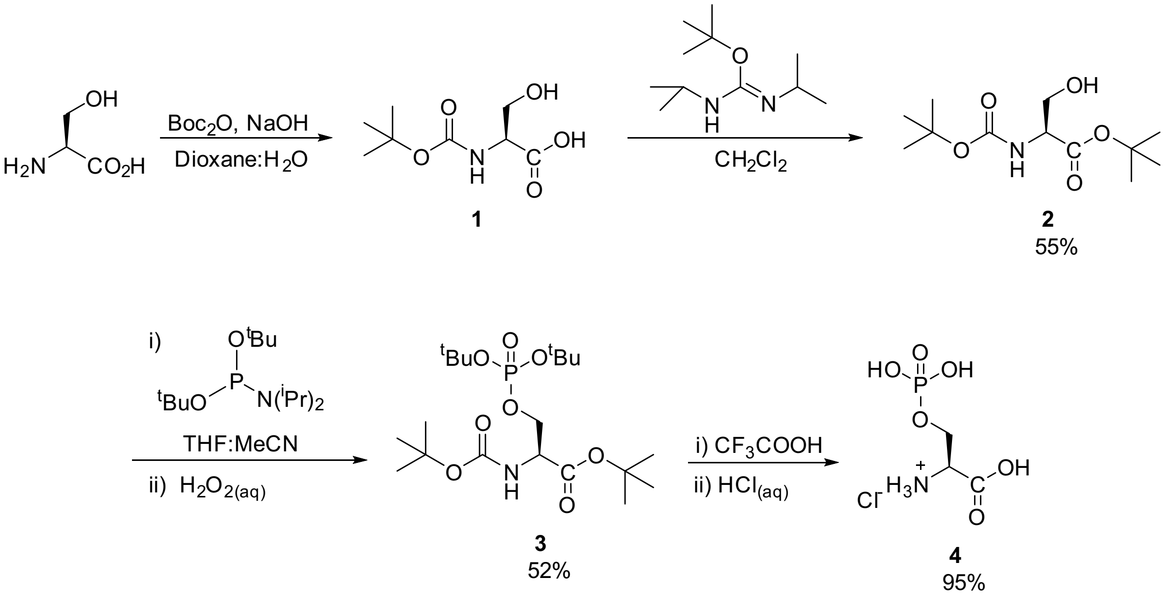

Fig. S7. Synthesis protocol of *O*-phospho-L-serine from L-serine that is either ${}^{13}C/{}^{15}N$ isotopically enriched (yielding Pser*) or having all isotopes at their natural abundance levels (yielding Pser).The percentages represent reaction yields.

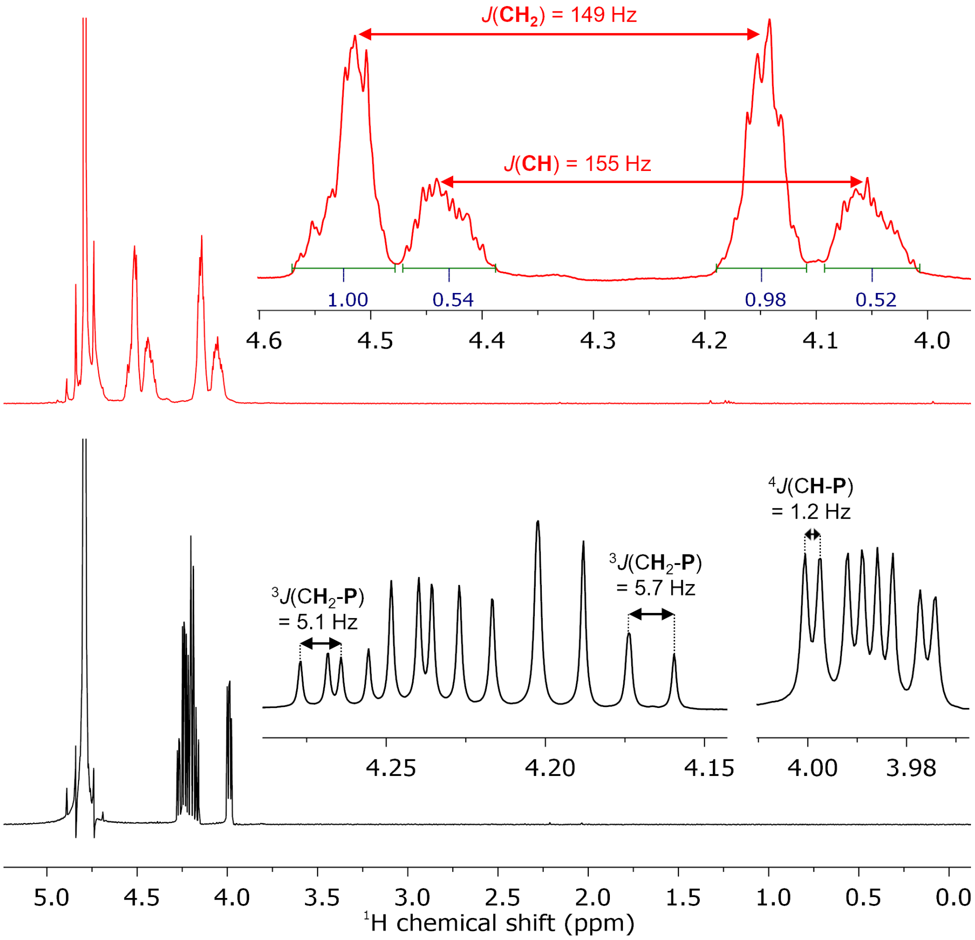

Fig. S8. Solution state ^{1}H NMR spectra recorded at 9.4 T from Pser* (red traces) and Pser (black traces) in D_2O buffered to pH=7.4 with a Gibco^{TM} PBS tablet. The spectra are normalized relative to the solvent peak at $\delta_H = 4.79$ ppm. The zoomed regions illustrate the splitting patterns from the as-indicated J couplings.

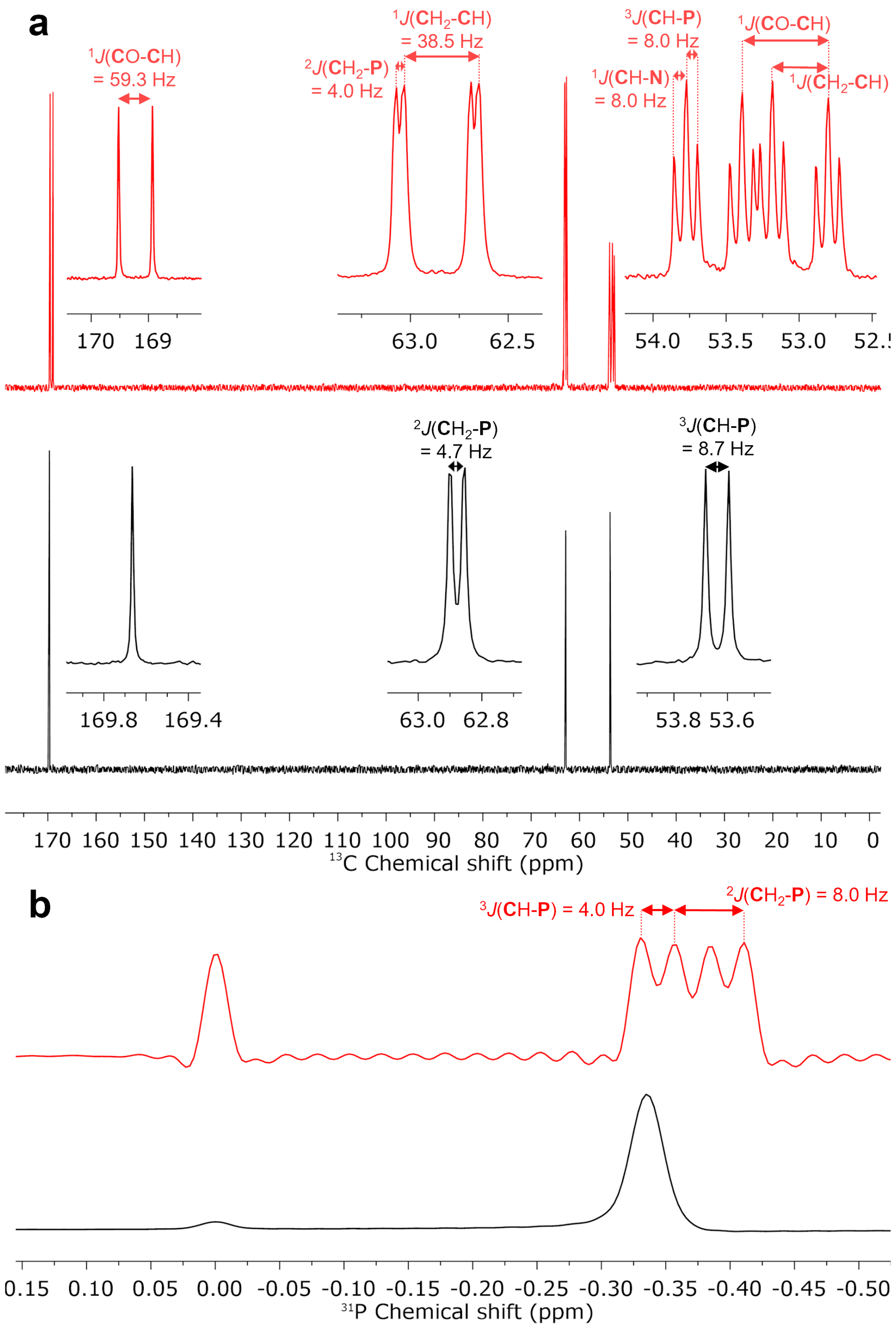

Fig. S9. Solution state (a) ^{13}C and (b) ^{31}P NMR spectra acquired at 9.4 T from Pser* (red traces) and Pser (black traces) in D_2O buffered to pH=7.4 with a Gibco^{TM} PBS tablet. The zoomed regions illustrate the splitting patterns from the as-indicated J couplings.

References

(S1) Fung, B. M.; Khitrin, A. K.; Ermolaev, K. An Improved Broadband Decoupling Sequence for Liquid Crystals and Solids. *J. Magn. Reson.* **2000**, *142*, 97–101.

(S2) Metz, G.; Wu, X. L.; Smith, S. O. Ramped-Amplitude Cross Polarization in Magic-Angle-Spinning NMR. *J. Magn. Reson., Ser. A* **1994**, *110*, 219–227.

(S3) Marion, D.; Ikura, M.; Tschudin, R.; Bax, A. Rapid Recording of 2D NMR Spectra without phase cycling. Application to the Study of Hydrogen Exchange in Proteins. *J. Magn. Reson.* **1989**, *85*, 393–399.

(S4) Gan, Z. $^{13}C/^{14}N$ Heteronuclear Multiple-Quantum Correlation with Rotary Resonance and REDOR Dipolar Recoupling. *J. Magn. Reson.* **2007**, *184*, 39–43.

(S5) Hu, B.; Trébosc, J.; Amoureux, J. P. Comparison of Several Hetero-Nuclear Dipolar Recoupling NMR Methods to be Used in MAS HMQC/HSQC. *J. Magn. Reson.* **2008**, *192*, 112–122.

(S6) Brinkmann, A.; Kentgens, A. P. M. Proton-Selective ^{17}O–^{1}H Distance Measurements in Fast Magic-Angle Spinning Solid-State NMR Spectroscopy for the Determination of Hydrogen Bond Lengths. *J. Am. Chem. Soc.* **2006**, *128*, 14758–14759.

(S7) Gullion, T.; Schaefer, J. Rotational-Echo Double-Resonance NMR. *J. Magn. Reson.* **1989**, *81*, 196–200.

(S8) Gullion, T. Measurement of Dipolar Interactions between Spin-1/2 and Quadrupolar Nuclei by Rotational-Echo, Adiabatic-Passage, Double-Resonance NMR. *Chem. Phys. Lett.* **1995**, *246*, 325–330.

(S9) Mathew, R.; Pujari-Palmer, M.; Guo, H.; Yu, Y.; Stevensson, B.; Engqvist, H.; Edén, M. Solid-State NMR Rationalizes the Bone-Adhesive Properties of Serine- and Phosphoserine-Bearing Calcium Phosphate Cements by Unveiling Their Organic/Inorganic Interface. *J. Phys. Chem. C* **2020**, *124*, 21512–21531.

(S10) Gullion, T.; Baker, D. B.; Conradi, M. S. New, Compensated Carr-Purcell Sequences. *J. Magn. Reson.* **1990**, *89*, 479–484.

(S11) Van Vleck, J. H. The Dipolar Broadening of Magnetic Resonance Lines in Crystals. *Phys. Rev.* **1948**, *74*, 1168–1183.

(S12) Bertmer, M.; Züchner, L.; Chan, J. C. C.; Eckert, H. Short and Medium Range Order in Sodium Aluminoborate Glasses: 2. Site Connectivities and Cation Distributions Studied by Rotational Echo Double Resonance NMR Spectroscopy. *J. Phys. Chem. B* **2000**, *104*, 6541–6553.

(S13) Strojek, W.; Kalwei, M.; Eckert, H. Dipolar NMR Strategies for Multispin Systems Involving Quadrupolar Nuclei: $^{31}P\{^{23}Na\}$ Rotational Echo Double Resonance (REDOR) of Crystalline Sodium Phosphates and Phosphate Glasses. *J. Phys. Chem. B* **2004**, *108*, 7061–7073.

(S14) Eckert, H.; Elbers, S.; Epping, J. D.; Janssen, M.; Kalwei, M.; Strojek, W.; Voigt, U. Dipolar Solid State NMR Approaches Towards Medium-Range Structure in Oxide Glasses. *Top. Curr. Chem.* **2005**, *246*, 195–233.

(S15) Stevensson, B.; Mathew, R.; Yu, Y.; Edén, M. Two Heteronuclear Dipolar Results at the Price of One: Quantifying Na/P Contacts in Phosphosilicate Glasses and Biomimetic Hydroxy-Apatite. *J. Magn. Reson.* **2015**, *251*, 52–56.

(S16) Edén, M. Update on ^{27}Al NMR Studies of Aluminosilicate Glasses. *Annu. Rep. NMR Spectrosc.* **2020**, *101*, 285–410.

(S17) Massiot, D.; Fayon, F.; Capron, M.; King, I.; Le Calvé, S.; Alonso, B.; Durand, J.-O.; Bujoli, B.; Gan, Z.; Hoatson, G. Modelling One- and Two-Dimensional Solid-State NMR Spectra. *Magn. Reson. Chem.* **2002**, *40*, 70–76.

(S18) Elliott, J. C.; Mackie, P. E.; Young, R. A. Monoclinic Hydroxyapatite. *Science* **1973**, *180*, 1055–1057.

(S19) Lin, T.-J.; Heinz, H. Accurate Force Field Parameters and pH Resolved Surface Models for Hydroxyapatite to Understand Structure, Mechanics, Hydration, and Biological Interfaces. *J. Phys. Chem. C* **2016**, *120*, 4975–4992.

(S20) Kay, M. I.; Young, R. A.; Posner, A. S. Crystal Structure of Hydroxyapatite. *Nature* **1964**, *204*, 1050–1052.

(S21) Martínez, L.; Andrade, R.; Birgin, E. G.; Martínez, J. M. PACKMOL: A Package for Building Initial Configurations for Molecular Dynamics Simulations. *J. Comput. Chem.* **2009**, *30*, 2157–2164.

(S22) Stevensson, B.; Edén, M. Metadynamics Simulations of the pH-Dependent Adsorption of Phosphoserine and Citrate on Disordered Apatite Surfaces: What Interactions Govern the Molecular Binding?. *J. Phys. Chem. B* **2021**, *125*, 11987–12003.

(S23) Abraham, M. J.; Murtola, T.; Schulz, R.; Páll, S.; Smith, J. C.; Hess, B.; Lindahl, E. GROMACS: High Performance Molecular Simulations Through Multi-Level Parallelism from Laptops to Supercomputers. *SoftwareX* **2015**, *1–2*, 19–25.

(S24) Allen, M. P.; Tildesley, D. J., *Computer Simulation of Liquids*, Clarendon Press, Oxford **1987**,.

(S25) Essmann, U.; Perera, L.; Berkowitz, M. L.; Darden, T.; Lee, H.; Pedersen, L. G. A Smooth Particle Mesh Ewald Method. *J. Chem. Phys.* **1995**, *103*, 8577–8593.

(S26) Bussi, G.; Donadio, D.; Parrinello, M. Canonical Sampling Through Velocity Rescaling. *J. Chem. Phys.* **2007**, *126*, 014101.

(S27) Barducci, A.; Bussi, G.; Parrinello, M. Well-Tempered Metadynamics: A Smoothly Converging and Tunable Free-Energy Method. *Phys. Rev. Lett.* **2008**, *100*, 020603.

(S28) Valsson, O.; Tiwary, P.; Parrinello, M. Enhancing Important Fluctuations: Rare Events and Metadynamics from a Conceptual Viewpoint. *Annu. Rev. Phys. Chem.* **2016**, *67*, 159–184.

(S29) Valsson, O.; Parrinello, M. Variational Approach to Enhanced Sampling and Free Energy Calculations. *Phys. Rev. Lett.* **2014**, *113*, 090601.

(S30) Tribello, G. A.; Bonomi, M.; Branduardi, D.; Camilloni, C.; Bussi, G. PLUMED 2: New Feathers for an Old Bird. *Comput. Phys. Comm.* **2014**, *185*, 604–613.

(S31) Do, T. N.; Carloni, P.; Varani, G.; Bussi, G. RNA/Peptide Binding Driven by Electrostatics—Insight from Bidirectional Pulling Simulations. *J. Chem. Theory Comput.* **2013**, *9*, 1720–1730.

(S32) Spitaleri, A.; Decherchi, S.; Cavalli, A.; Rocchia, W. Fast Dynamic Docking Guided by Adaptive Electrostatic Bias: The MD-Binding Approach. *J. Chem. Theory Comput.* **2018**, *14*, 1727–1736.

(S33) Bach, F.; Moulines, E. Non-Strongly-Convex Smooth Stochastic Approximation with Convergence Rate $O(1/n)$. in Advances in Neural Information Processing Systems eds. C. J. C. Burges, L. Bottou, M. Welling, Z. Ghahramani, and K. Q. Weinberger. *Curran Associates, Inc., Red Hook, NY* **2013**, *26*, 773–781.

(S34) Laio, A.; Gervasio, F. L. Metadynamics: a Method to Simulate Rare Events and Reconstruct the Free Energy in Biophysics, Chemistry and Material Science. *Rep. Prog. Phys.* **2008**, *71*, 126601.

(S35) Bonomi, M.; Barducci, A.; Parrinello, M. Reconstructing the Equilibrium Boltzmann Distribution from Well-Tempered Metadynamics. *J. Comp. Chem.* **2009**, *30*, 1615–1621.

(S36) Tiwary, P.; Parrinello, M. A Time-Independent Free Energy Estimator for Metadynamics. *J. Phys. Chem. B* **2015**, *119*, 736–742.

(S37) Debye, V. P.; Hückel, E. Zur Theorie der Elektrolyte. *Phys. Z.* **1923**, *24*, 185–206.

(S38) Wright, M. R., *An Introduction to Aqueous Electrolyte Solutions*, Wiley London, GB 1st edition **2007**,.

(S39) Maltsev, S.; Duer, M. J.; Murray, R. C.; Jaeger, C. A Solid-State NMR Comparison of the Mineral Structure in Bone from Diseased Joints in the Horse. *J. Mater. Sci.* **2007**, *42*, 8804–8810.

(S40) Wang, Y.; von Euw, S.; Fernandes, F. M.; Cassaignon, S.; Selmane, M.; Laurant, G.; Pehau-Arnaudet, G.; Coelho, C.; Bonhomme-Coury, L.; Giraud-Guille, M.-M.; Babonneau, F.; Azaïs, T.; Nassif, N. Water-Mediated Structuring of Bone Apatite. *Nature Mater.* **2013**, *12*, 1144–1153.

(S41) von Euw, S.; Wang, Y.; Laurent, G.; Drouet, C.; Babonneau, F.; Nassif, N.; Azaïs, T. Bone Mineral: New Insights into its Chemical Composition. *Sci. Rep.* **2019**, *9*, 8456.

(S42) Edén, M. Structure and Formation of Amorphous Calcium Phosphate and its Role as Surface Layer of Nanocrystalline Apatite: Implications for Bone Mineralization. *Materialia* **2021**, *17*, 101107.

(S43) Eichert, D.; Sfihi, H.; Combes, C.; Rey, C. Specific Characterisitics of Wet Nanocrystalline Apatites: Consequences on Biomaterials and Bone Tissue. *Key. Eng. Mater.* **2004**, *254–256*, 927–930.

(S44) Jäger, C.; Welzel, T.; Meyer-Zaika, W.; Epple, M. A Solid-State NMR Investigation of the Structure of Nanocrystalline Hydroxyapatite. *Magn. Reson. Chem.* **2006**, *44*, 573–580.

(S45) Rey, C.; Combes, C.; Drouet, C.; Sfihi, H.; Barroug, A. Physico-Chemical Properties of Nanocrystalline Apatites: Implications for Biominerals and Biomaterials. *Mater. Sci. Eng. C* **2007**, *27*, 198–205.

(S46) von Euw, S.; Ajili, W.; Chan-Chang, T.-H.-C.; Delices, A.; Laurent, G.; Babonneau, F.; Nassif, N.; Azaïs, T. Amorphous Surface Layer *versus* Transient Amorphous Precursor Phase in Bone – A Case Study Investigated by Solid-State NMR Spectroscopy. *Acta Biomater.* **2017**, *59*, 351–360.

(S47) Huq, N. L.; Cross, K. J.; Reynolds, E. C. Molecular Modelling of a Multiphosphorylated Sequence Motif Bound to Hydroxyapatite Surfaces. *J. Mol. Model.* **2000**, *6*, 35–47.

(S48) de Leeuw, N. H.; Rabone, J. A. L. Molecular Dynamics Simulations of the Interaction of Citric Acid with the Hydroxyapatite (0001) and ($01\bar{1}0$) Surfaces in an Aqueous Environment. *CrystEngComm* **2007**, *9*, 1178–1186.

(S49) Rimola, A.; Corno, M.; Zicovich-Wilson, C. M.; Ugliengo, P Ab Initio Modeling of Protein/Biomaterial Interactions: Glycine Adsorption at Hydroxyapatite Surfaces. *J. Am. Chem. Soc.* **2008**, *130*, 16181–16183.

(S50) Almora-Barrios, N.; Austen, K. F.; de Leeuw, N. H. Density Functional Theory Study of the Binding of Glycine, Proline, and Hydroxyproline to the Hydroxyapatite (0001) and ($01\bar{1}0$) Surfaces. *Langmuir* **2009**, *25*, 5018–5025.

(S51) Wang, Z.; Xu, Z.; Zhao, W.; Chen, W.; Miyoshi, T.; Sahai, N. Isoexergonic Conformations of Surface-Bound Citrate Regulated Bioinspired Apatite Nanocrystal Growth. *ACS Appl. Mater. Interfaces* **2016**, *8*, 28116–28123.

(S52) Corno, M.; Rimola, A.; Bolis, V.; Ugliengo, P. Hydroxyapatite as a Key Biomaterial: Quantum-Mechanical Simulation of its Surfaces in Interaction with Biomolecules. *Phys. Chem. Chem. Phys.* **2010**, *12*, 6309–6329.

(S53) Zhang, H.-p.; Lu, X.; Leng, Y.; Fang, L.; Qu, S.; Feng, B.; Weng, J.; Wang, J. Molecular Dynamics Simulations on the Interaction between Polymers and Hydroxyapatite with and without Coupling Agents. *Acta Biomater.* **2009**, *5*, 1169–1181.

(S54) Xu, Z.; Yang, Y.; Wang, Z.; Mkhonto, D.; Shang, C.; Liu, Z.-P.; Cui, Q.; Sahai, N. Small Molecule-Mediated Control of Hydroxyapatite Growth: Free Energy Calculations Benchmarked to Density Functional Theory. *J. Comput. Chem.* **2014**, *35*, 70–81.

(S55) Xu, Z.; Wei, Q.; Zhao, W.; Cui, Q.; Sahai, N. Essence of Small Molecule-Mediated Control of Hydroxyapatite Growth: Free Energy Calculations of Amino Acid Side Chain Analogues. *J. Phys. Chem. C* **2018**, *122*, 4372–4380.

(S56) Hu, Y.-Y.; A, Rawal; Schmidt-Rohr, K. Strongly Bound Citrate Stabilizes the Apatite Nanocrystals in Bone. *Proc. Natl. Acad. Sci. U.S.A.* **2010**, *107*, 22425–22429.

(S57) King, R. B.; Sadanani, N. D. Dialkylaminophosphines. *J. Chem. Soc., Chem. Commun.* **1984**, *1984*, 955–956.

(S58) Nakagawa, Y.; Saitou, A.; Aoyagi, T.; Naito, M.; Ebara, M. Apoptotic Cell Membrane-Inspired Polymer for Immunosuppression. *ACS Macro Lett.* **2017**, *6*, 1020–1024.

(S59) Ernst, R. R.; Bodenhausen, G.; Wokaun, A., *Principles of Nuclear Magnetic Resonance in One and Two Dimensions*, Clarendon Press Oxford **1987**,.

(S60) Pogliani, L.; Ziessow, D.; Krüger, C. Conformational Study of Phosphoserine in Aqueous Solution. I—^{13}C N.m.r. Results. *Org. Magn. Reson.* **1977**, *9*, 504–507.

(S61) McIntosh, L. P.; Kang, H.-S.; Okon, M.; Nelson, M. L.; Graves, B. J.; Brutscher, B. Detection and Assignment of Phosphoserine and Phosphothreonine Residues by ^{13}C–^{31}P Spin-Echo Difference NMR Spectroscopy. *J. Biomol. NMR* **2009**, *43*, 31–47.

(S62) Boyd, D. R.; Stubbs, M. E.; Thompson, N. J.; Yeh, H. J. C.; Jerina, D. M.; Wasylishen, R. E. Nitrogen-15 Coupling Constants: Geminal Coupling, $^2J(^{15}\mathrm{NH})$, in *cis*- and *trans*-Aldonitrones and Vicinal Coupling, $^3J(^{15}\mathrm{NH})$, in *cis*- and *trans*-Ketimines, Oxaziridines and Nitrones. *Org. Magn. Reson.* **1980**, *14*, 528–533.

(S63) Deev, S. L.; Khalymbadzha, I. A.; Shestakova, T. S.; Charushin, V. N.; Chupakhin, O. N. ^{15}N Labeling and Analysis of ^{13}C–^{15}N and ^{1}H–^{15}N Couplings in Studies of the Structures and Chemical Transformations of Nitrogen Heterocycles. *RSC Adv.* **2019**, *9*, 26856–26879.

(S64) Brauer, M.; Sykes, B. D. Phosphorus-31 Nuclear Magnetic Resonance Studies of Phosphorylated Proteins. *Meth. Enzymol.* **1984**, *107*, 36–81.

(S65) Sogn, J. A.; Craig, L. C.; Gibbons, W. A. ^{13}C–^{13}C Coupling Constants in a Series of ^{13}C-Enriched Amino Acids. *J. Am. Chem. Soc.* **1974**, *96*, 4694–4696.

(S66) Wasylishen, R. E. ^{15}N–^{13}C Spin–Spin Coupling Constants in some Aniline Derivatives. *Can. J. Chem.* **1976**, *54*, 833–839.

(S67) Yasar, O. F.; Liao, W.-C.; Stevensson, B.; Edén, M. Structural Role and Spatial Distribution of Carbonate Ions in Amorphous Calcium Phosphate. *J. Phys. Chem. C* **2021**, *125*, 4675–4693.

(S68) Yasar, O. F.; Liao, W.-C.; Mathew, R.; Yu, Y.; Stevensson, B.; Liu, Y.; Shen, Z.; Edén, M. The Carbonate and Sodium Environments in Precipitated and Biomimetic Calcium Hydroxy-Carbonate Apatite Contrasted with Bone Mineral: Insights from Solid-State NMR. *J. Phys. Chem. C* **2021**, *125*, 10572–10592.

(S69) Li, S.-L.; Wang, L.-H.; Huang, S.-J.; Chan, J. C. C. Hydrogen Phosphates Play a Critical Structrual Role in Amorphous Calcium Phosphates. *Chem. Comm.* **2022**, *in press*.

(S70) MacKerell Jr., A. D.; Bashford, D.; Bellott, M.; Dunbrack Jr., R. L.; Evanseck, J. D.; Field, M. J.; Fischer, S.; Gao, J.; Guo, H.; Ha, S.; Joseph-McCarthy, D.; Kuchnir, L.; Kuczera, K.; Lau, F. T. K.; Mattos, C.; Michnick, S.; Ngo, T.; Nguyen, D. T.; Prodhom, B.; Reiher, W. E.; Roux, B.; Schlenkrich, M.; Smith, J. C.; Stote, R.; Straub, J.; Watanabe, M.; Wiórkiewicz-Kuczera, J.; Yin, D.; Karplus, M. All-Atom Empirical Potential for Molecular Modeling and Dynamics Studies of Proteins. *J. Phys. Chem. B* **1998**, *102*, 3586–3616.

(S71) Feng, M.-H.; Philippopoulos, M.; MacKerell, Jr., A. D.; Lim, C. Structural Characterization of the Phosphotyrosine Binding Region of a High-Affinity SH2 Domain-Phosphopeptide Complex by Molecular Dynamics Simulation and Chemical Shift Calculations. *J. Am. Chem. Soc.* **1996**, *118*, 11265.

(S72) Jorgensen, W. L.; Chandrasekhar, J.; Madura, J. D.; Impey, R. W.; Klein, M. L. Comparison of Simple Potential Functions for Simulating Liquid Water. *J. Chem. Phys.* **1983**, *79*, 926–935.

(S73) Mathew, R.; Stevensson, B.; Edén, M Refined Structures of *O*-Phospho-L-serine and Its Calcium Salt by New Multinuclear Solid-State NMR Crystallography Methods. *J. Phys. Chem. B* **2021**, *125*, 10985–11004.
